# Low‐Dimensional MOF Nanoarchitectonics: Progress in MOF‐2D Material Hybrid Architectures for Energy Conversion and Storage

**DOI:** 10.1002/adma.202521053

**Published:** 2026-03-09

**Authors:** Prashant Dubey, Norman C.‐R. Chen, Xiangyang Liu, Yongqi Yin, Keisuke Shirasaki, Kevin C.‐W. Wu, Yingji Zhao, Yusuke Yamauchi

**Affiliations:** ^1^ Department of Materials Process Engineering Graduate School of Engineering Nagoya University Nagoya Japan; ^2^ Molecular Science and Technology Program Taiwan International Graduate Program Academia Sinica Taipei Taiwan; ^3^ International Graduate Program of Molecular Science and Technology (NTU‐MST) National Taiwan University Taipei Taiwan; ^4^ Key Laboratory for Photonic and Electronic Bandgap Materials Ministry of Education School of Physics and Electronic Engineering Harbin Normal University Harbin China; ^5^ Department of Chemical Engineering and Materials Science Yuan Ze University Chung‐Li Taoyuan Taiwan; ^6^ Department of Chemical Engineering Chung Yuan Christian University Taoyuan City Taiwan; ^7^ Australian Institute for Bioengineering and Nanotechnology (AIBN) The University of Queensland Brisbane Queensland Australia; ^8^ Department of Convergent Biotechnology & Advanced Materials Science Kyung Hee University Gyeonggi‐do Seoul South Korea

**Keywords:** electrochemistry, energy storage, interface engineering, MOF‐2D hybrid, multifunctional hybrid materials

## Abstract

The integration of metal‐organic frameworks (MOFs) and two‐dimensional (2D) materials is a powerful and rapidly advancing strategy for creating multifunctional hybrid materials. Unlocking their full potential requires overcoming the intrinsic limitations of each component, specifically the poor electrical conductivity of MOFs and the restacking of 2D nanosheets. This review provides a systematic overview of the pivotal role of dimensional interface engineering in addressing this challenge. A systematic analysis of synthesis methodologies is presented, including direct growth, encapsulation, layer‐by‐layer assembly, and MOF‐derived transformations, correlating architectural control with the fundamental structure–property relationships that govern mass transport, electronic coupling, and defect chemistry. The remarkable impact of these engineered hybrids is then highlighted across key applications in high‐performance electrocatalysis for crucial energy conversion reactions and in advanced energy storage systems such as batteries and supercapacitors. A central theme is that the deliberate manipulation of the interface is the critical determinant for unlocking synergistic enhancements in charge and mass transport, structural stability, and redox activity. Finally, this review concludes by critically assessing persistent challenges in scalability, stability, and atomic‐level precision, while outlining the future opportunities poised to propel MOF‐2D hybrids from laboratory innovations to transformative technologies.

## Graphical abstract

This review highlights the integration of metal‐organic frameworks (MOFs) and two‐dimensional (2D) materials through dimensional interface engineering. By addressing intrinsic limitations like poor conductivity and agglomeration, these hybrid architectures optimize interfacial charge and mass transport. The resulting synergistic effects drive superior performance in critical energy applications, including high‐efficiency electrocatalysis and advanced energy storage systems.

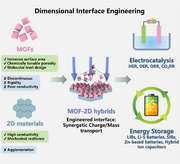

## 1 Introduction

The design of synergistic heterostructures by integrating materials with disparate, yet complementary properties represents a paramount strategy in modern materials science. A compelling manifestation of this approach lies at the convergence of metal‐organic frameworks (MOFs) and two‐dimensional (2D) materials [1, 2]. MOFs, celebrated for their immense surface areas and chemically tunable porosity [3, 4, 5], offer an unparalleled platform for molecular‐level design [6, 7, 8, 9, 10] but are frequently hampered by their insulating nature and limited stability, which constrains their utility in charge‐transport‐dependent applications [11, 12, 13, 14]. Conversely, 2D materials, such as graphene and MXenes, provide exceptional electrical conductivity and mechanical resilience but suffer from a propensity to agglomerate, a phenomenon that drastically curtails their accessible surface area and functional sites [15, 16, 17]. The hybridization of these two material classes thus presents a powerful solution, creating integrated systems that circumvent these intrinsic trade‐offs [18, 19].

At the heart of these hybrid materials lies the dimensional interface; the critical nexus where the crystalline, porous framework of the MOF meets the atomically thin, conductive 2D sheet. This interface is far from a passive boundary; it is an active zone where fundamental electronic and chemical processes are dictated. Engineering this interface, through the precise control of interactions ranging from non‐covalent (van der Waals, *π*–*π* stacking) to robust covalent or coordination bonding, allows for the deliberate modulation of charge transfer kinetics, the creation of unique active sites, and the optimization of mass transport pathways [12, 20, 21]. The rational design of this interfacial region is therefore the key determinant of the hybrid's ultimate performance, transforming simple composites into highly efficient, functional materials.

It is worth noting that while intrinsically conductive 2D MOFs have recently garnered significant attention for their combined porosity and electrical transport properties [22, 23], the construction of MOF‐2D material hybrids remains scientifically critical and practically advantageous. First, the electrical conductivity of graphene or MXenes (typically > 10^4^ S cm^−1^) far surpasses that of most semiconducting MOFs [24, 25], ensuring superior charge transfer kinetics for high‐rate energy applications. Second, conductive MOFs are often limited by complex synthetic demands and a restricted library of ligands. In contrast, hybridization strategies offer a universal platform to impart conductivity to the vast majority of electrically insulating yet catalytically active MOFs (e.g., ultra‐high surface area Zr‐based MOFs or ZIFs), thereby breaking the limitation of material selection [12]. Furthermore, the 2D backbone serves as a robust mechanical support, enhancing the structural integrity and chemical stability of MOFs that are otherwise prone to collapse during long‐term electrochemical cycling [26]. Thus, MOF‐2D hybrids provide a unique synergistic pathway to simultaneously maximize porosity, conductivity, and stability, surpassing what is achievable by single‐component materials.

The evolution of synthesis has enabled a transition from rudimentary physical mixtures to sophisticated, highly ordered architectures. This includes the growth of MOFs as conformal coatings on 2D nanosheets (core‐shell), the intercalation of 2D materials within MOF superstructures, or the layer‐by‐layer assembly of the two components. The pinnacle of this architectural control is realized in the use of ultrathin 2D MOF nanosheets as fundamental building blocks. This advanced strategy facilitates the fabrication of all‐2D heterostructures with atomically defined interfaces, maximizing the density of synergistic sites while minimizing diffusion lengths for both charge and mass. Such integrated systems represent the frontier of MOF‐2D hybrid design, promising unprecedented performance enhancements [27, 28, 29, 30].

**FIGURE 1:**
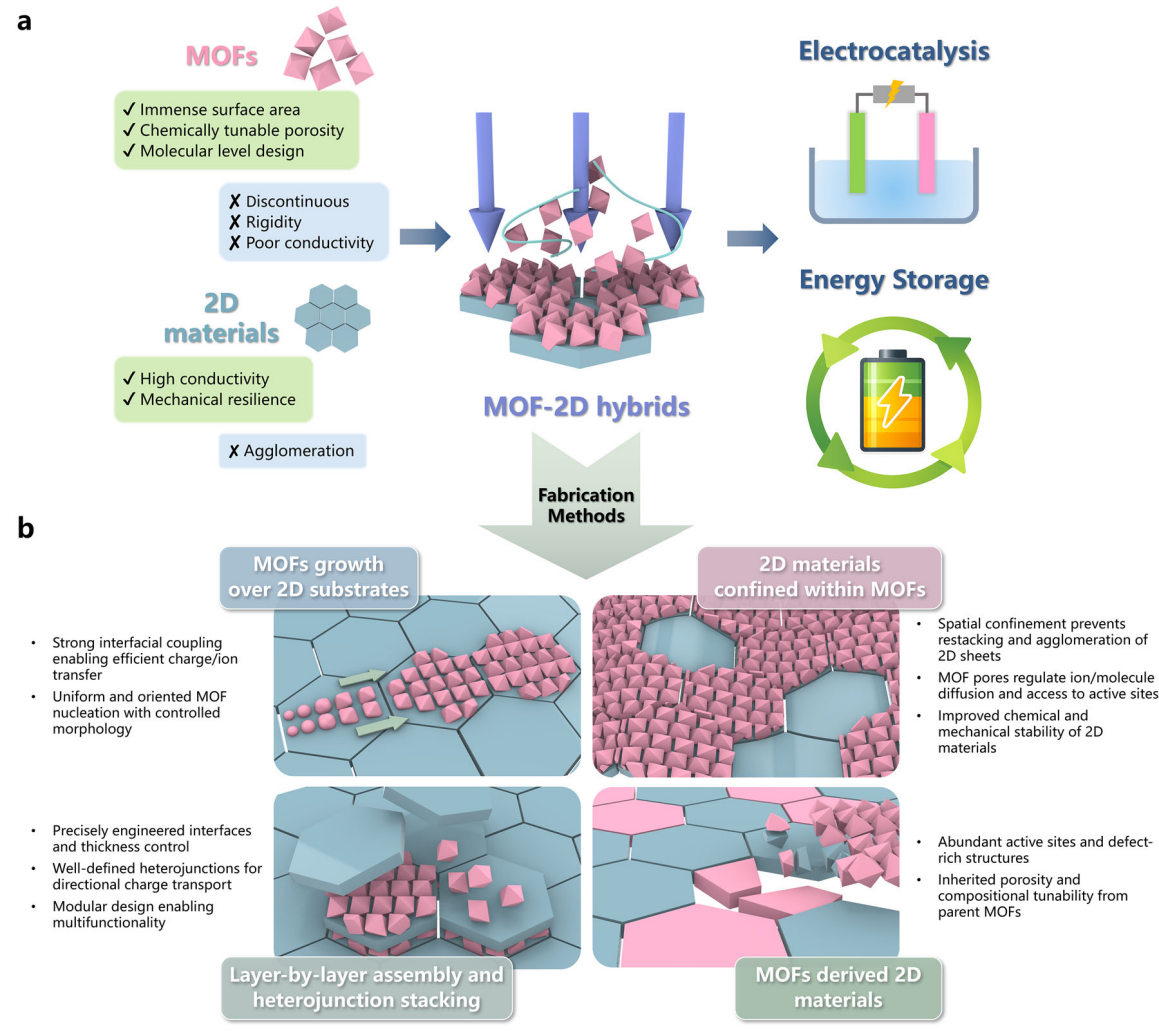
Schematic illustration of the (a) concept of MOFs and 2D materials hybrid and (b) fabrication methods of different types of MOFs and 2D materials hybrid architectures.

Several review articles have focused on hybrid materials formed by MOFs combined with specific classes of 2D materials, particularly graphene and MXenes [12, 20, 26, 29, 31, 32, 33, 34]; however, review articles addressing MOF hybrids across the full spectrum of 2D materials remain scarce. To the best of our knowledge, a review article published in 2016 by the Zhang group addressed MOF‐2D material hybrids [27], primarily discussing their synthesis strategies and applications in sensing, storage, catalysis, and separation. Nevertheless, this review was relatively brief and is now dated, with limited emphasis on their potential in energy storage and electrocatalysis. More recently, a review article published in 2025 highlighted the integration of MOFs with 2D materials for flame‐retardant and sensing applications [35]. The present review article discusses the recent advancements in the rapidly expanding field of MOF and 2D materials hybrids, as well as the critical significance of architectural design and synthesis methodologies. The central scope of this review is to provide a unified and critical analysis of the recent breakthroughs in the field of MOF‐2D hybrid materials, with a deliberate focus on the pivotal role of dimensional interface engineering [36]. We systematically examine advanced synthesis methodologies that grant precise control over the hybrid architecture and interfacial properties (Figure 1a). We then highlight the remarkable impact of these tailored materials on pressing challenges in energy storage, including metal‐ion batteries and supercapacitors, and in electrocatalysis, focusing on the oxygen evolution (OER), hydrogen evolution (HER), and oxygen reduction (ORR) reactions. Finally, we conclude by addressing the persistent challenges and outlining the future perspectives that will shape the next generation of these extraordinary materials.

## 2 Classification of MOFs and 2D Materials Hybrid Architectures

**FIGURE 2:**
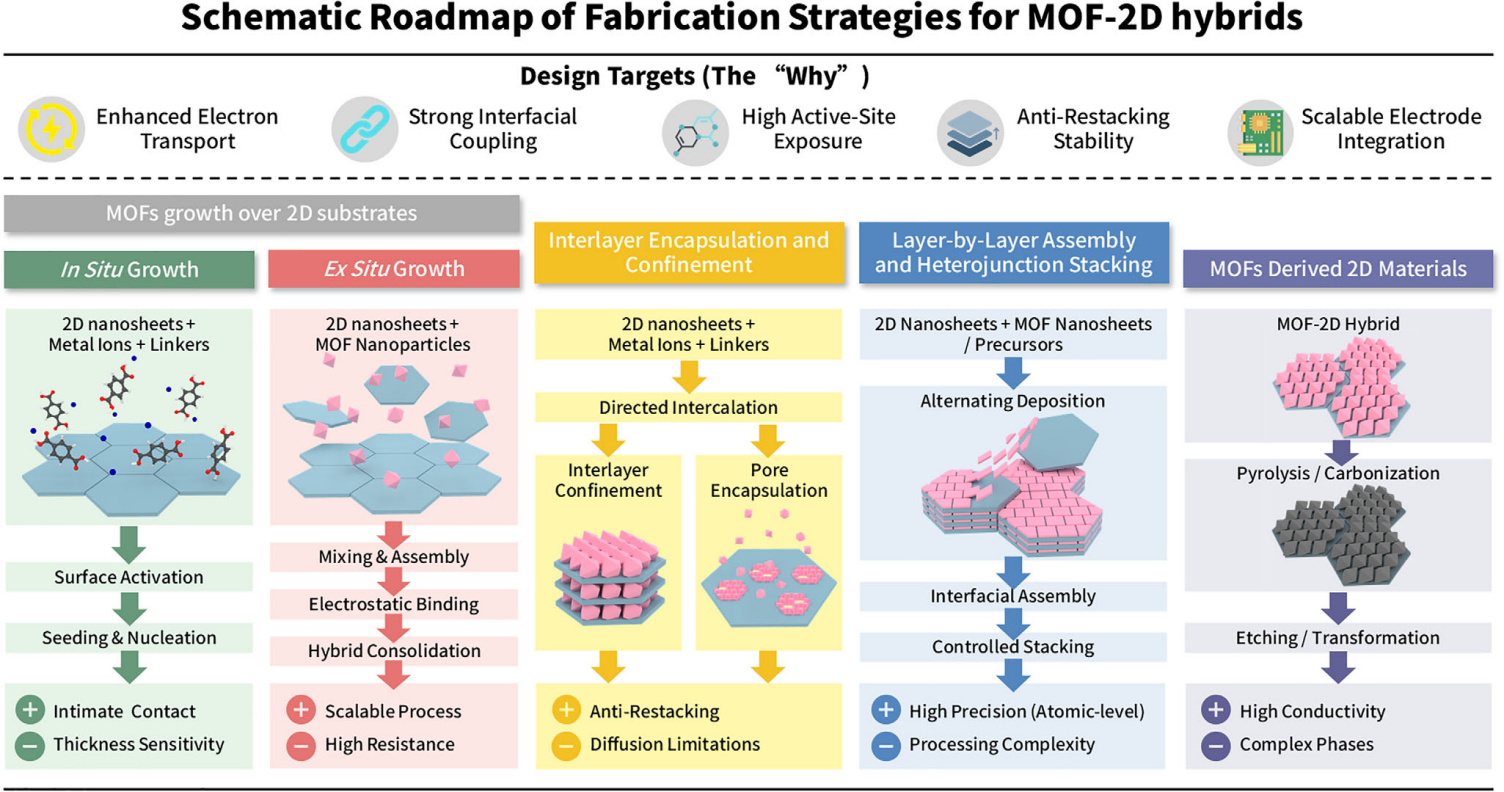
Schematic roadmap of fabrication strategies for MOF‐2D hybrids.

The integration of MOFs and 2D materials has given rise to a diverse landscape of hybrid architectures, each with unique structural features and intriguing properties. The classification of these hybrids is essential for understanding the underlying mechanisms that govern their formation, stability, and functionality, as well as for guiding the rational design of next‐generation materials. Broadly, MOF‐2D material hybrids can be categorized based on the spatial arrangement and interaction of their components, including MOF growth over 2D substrates, 2D materials confined within MOFs (or MOFs encapsulated 2D materials), layer‐by‐layer assembly and heterojunction stacking, and MOF‐derived 2D materials (Figure 1b). To provide a clearer overview of the material preparation schemes, we further summarize the fabrication methods, design targets, and their associated advantages and limitations in Figure 2.

### 2.1 MOFs Growth Over 2D Substrates

The direct growth of MOFs on 2D materials represents a basic approach in the engineering of hybrid architecture, enabling synergistic integration of the unique properties of both materials. This strategy is pivotal for multiple applications, as it leverages the high surface area, tunable porosity, and chemical functionality of MOFs along with the exceptional conductivity, mechanical strength, and processability of 2D materials such as graphene‐based materials, MXenes, and transition metal dichalcogenides (TMDs). The two principal methodologies for achieving this integration are in situ and ex situ growth, each offering distinct advantages and challenges regarding interfacial contact, crystallinity, and scalability.

#### 2.1.1 In Situ Growth

In situ growth involves the direct nucleation and crystallization of MOFs from precursor solutions on the surface of 2D materials. This process typically takes place under solvothermal, hydrothermal, or ambient conditions, where the 2D substrate is immersed in a solution comprising metal ions and organic linkers. The surface chemistry of the 2D material plays a crucial role in directing the nucleation and subsequent growth of the MOF. Functional groups such as carboxyl, hydroxyl, or amine moieties on graphene oxide (GO) or MXene surfaces can act as anchoring sites for metal ions, facilitating heterogeneous nucleation and promoting strong interfacial adhesion. A prominent example is the first in situ growth of MOF‐5 over GO by Petit and coworkers in 2009 [37]. Here, the secondary building unit, tetrahedral ZnO_4_ of the MOF can form hydrogen bonds with the hydroxyl groups present on the GO surface, serving as nucleation sites for the growth of MOF‐5 crystals on the GO sheets. Furthermore, in situ growth of Ni‐MOF on graphene‐like materials, as reported by Liu et al. [38], where the presence of the 2D substrate influenced the morphology and crystallinity of the resulting hybrid, yielding improved electrochemical performance. Similarly, several MOFs have been successfully grown on GO, resulting in composites with enhanced adsorption and separation properties. The in situ approach often leads to uniform MOF coverage, effective interfacial interactions, and the possibility of controlling crystal orientation, which are critical for optimizing charge and mass transfer in hybrid systems [12].

**FIGURE 3:**
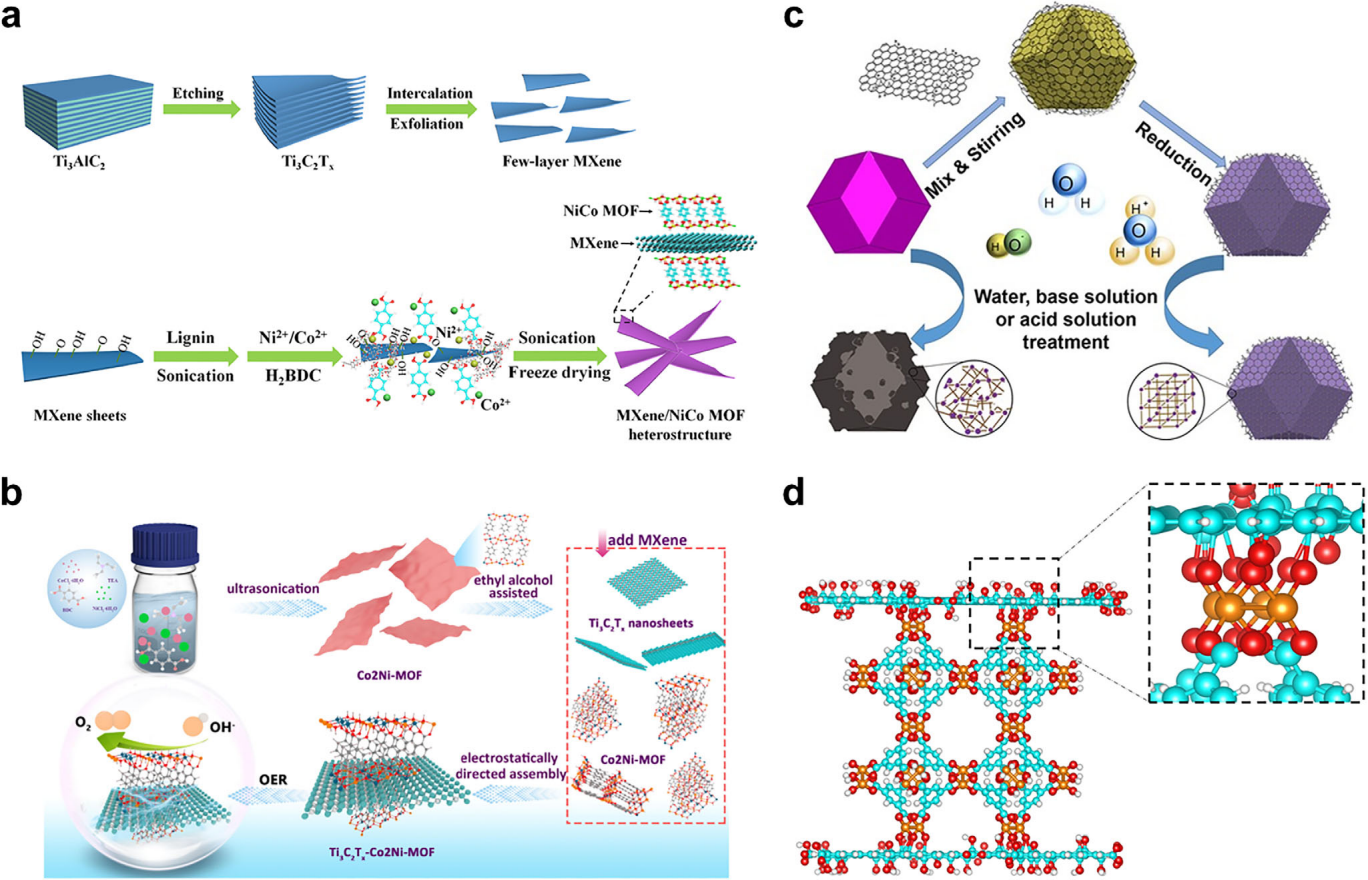
(a) The fabrication process of the few‐layer Ti_3_C_2_T_x_ sheets and Ti_3_C_2_T_x_/NiCo MOF heterostructure. Reproduced with permission [39]. Copyright 2022, Elsevier. (b) Schematic illustration of the synthesis process of Co_2_Ni‐MOF@MXene. Reproduced with permission [47]. Copyright 2023, Elsevier. (c) Schematic illustration of the reduced graphene oxide coating on the surface of UiO‐66‐NH_2_ for the stability enhancement. Reproduced with permission [42]. Copyright 2017, Wiley‐VCH. (d) The sandwich structure of the Cu‐BTC/GO composite model. Reproduced with permission [49]. Copyright 2025, Springer Nature.

The in situ growth method is a prominent strategy for cultivating MOFs on 2D substrates like MXenes. The abundant surface terminations on MXene sheets, such as ─O, ─OH, and ─F, serve as ideal nucleation sites, facilitating the uniform growth of MOF nanosheets. For instance, Jin et al. successfully engineered a hierarchical Ti_3_C_2_T_x_/NiCo‐MOF heterostructure by leveraging this approach (Figure 3a). The key to this synthesis is the formation of stable chemical bonds between the carboxylate linkers of the MOF precursors and the hydroxyl groups on the Ti_3_C_2_T_x_ surface. This creates a robust and well‐anchored interface, which is crucial for preventing the delamination of the MOF layer during electrochemical cycling [39].

Furthermore, nucleation control during in situ MOF growth on 2D substrates is critical to achieving the desired morphology and interfacial properties. Surface chemistry of the 2D material native functional groups on GO (carboxylates, hydroxyls) or termination groups on MXenes (─O, ─OH, ─F) can act as hetero‐nucleation sites that dramatically lower the energy barrier for MOF formation on the surface, fostering conformal coverage and oriented crystallization. For instance, Gong et al. [40] explicitly report that F^−^ and O^−^ surface functional groups on the MXene provide numerous sites for preferential nucleation and growth of the MOF, facilitating in situ MOF deposition without aggregation. Similarly, the surface functional groups (C─O, C═O) of graphene‐based materials can direct the nucleation and oriented growth of MOF crystals, enabling highly ordered sheet‐like architectures [37, 41]. Synthesis factors, such as precursor supersaturation, solvent polarity, temperature, mixing order, and the usage of modulators (monocarboxylic acids like acetic or formic acid), are critical variables. Low supersaturation and the presence of modulators often decrease nucleation and promote fewer, bigger, and more directed crystallites, whereas high supersaturation results in fast nucleation, generating many tiny nuclei and perhaps discontinuous growth. The use of modulators, such as monocarboxylic acids or surfactants, can suppress secondary nucleation and favor anisotropic growth, enabling the formation of oriented MOF nanosheets or thin films. For example, the addition of acetic acid as a modulator during the in situ growth of UiO‐66‐NH_2_ or ZIF‐8 over GO led to the formation of thin, uniform MOF layers with controlled thickness and orientation (Figure 2c), which are desirable for membrane and sensing applications [42].

Additional strategies include physical confinement and templating. Interlayer spaces in stacked GO or pillared 2D assemblies bias nucleation into ultrathin MOF morphologies, and soft templates (surfactants, polymer) can influence vertical growth or produce arrays of MOF nanosheets aligned normal to the 2D substrate [43]. Control of nucleation is particularly important when the goal is to obtain ultrathin MOF films or highly oriented films (for directional ion transport or anisotropic catalytic activity). These mechanistic insights have practical implications. For instance, the ability to engineer a thin MOF coating on a conductive 2D support can simultaneously provide molecular sieving and high electrical conductivity, a combination that is highly prized for electrochemical sensors and selective electrocatalysis.

In situ growth offers key advantages, including uniform MOF coverage, strong interfacial adhesion, tunable crystal orientation, and direct control over heterogeneous nucleation at the MOF‐2D interface, which collectively enhance charge and mass transport in MOF‐2D material hybrids. Despite their effectiveness, nucleation‐control strategies are highly sensitive to surface chemistry and synthesis parameters, and insufficient control can compromise reproducibility, resulting in nonuniform coverage, excessive MOF loading, pore blockage, or unintended bulk MOF growth, while harsh solvothermal conditions may further degrade sensitive 2D materials [44]. Moreover, excessive use of modulators or surfactants may block active sites or introduce impurities, which can impede mass and charge transport. These drawbacks can be mitigated by regulating precursor concentration, modulator dosage, growth time, and surface functionalization of the 2D material, as well as by employing mild or room‐temperature synthesis routes to achieve well‐defined, stable hybrid architectures with controlled orientation.

#### 2.1.2 Ex Situ Growth

Ex situ growth, in contrast, involves the independent synthesis of MOF crystals, which are subsequently deposited or assembled onto pre‐formed 2D substrates. This approach offers greater flexibility in selecting and combining different MOFs and 2D materials, as it decouples the synthesis conditions for each component. Ex situ methods include physical mixing, or post‐synthetic modification, where the MOF and 2D material are brought into contact through solution processing, filtration, or mechanical pressing [45].

While ex situ growth can result in weaker interfacial interactions compared to in situ methods, it is advantageous for preserving the intrinsic crystallinity, porosity, surface chemistry, and electronic properties of both the MOF and the 2D substrate. This is particularly beneficial for developing multifunctional systems where each component contributes a distinct role, such as adsorption, conductivity, or catalytic activity. For example, the fabrication of MIL‐101/GO hybrid by ex situ mixing has yielded materials with improved water adsorption capacity [46], owing to the synergistic effect of the high surface area of MIL‐101 and the conductive network of GO. Similarly, Co_2_Ni‐MOF/MXene hybrid was synthesized via an ex situ method using electrostatic interactions between the parent material for an oriented assembly (Figure 3b) [47]. Additionally, ex situ assembly allows for the integration of functional additives, such as metal nanoparticles or polymers, to further tailor the properties of the hybrid.

Despite these advantages, ex situ growth can lead to weak interfacial adhesion, nonuniform dispersion of MOF particles, and limited charge or mass transfer across the MOF‐2D material interface [21]. These challenges can be mitigated through surface functionalization of either component, the use of coupling agents or binders to enhance interfacial compatibility, and post‐assembly treatments such as mild thermal annealing or solvent‐assisted infiltration. Such strategies improve interfacial contact, structural uniformity, and overall performance, making ex situ growth a practical and scalable route for engineering MOF‐2D material hybrids tailored to specific applications. The choice between in situ and ex situ routes, therefore, represents a fundamental trade‐off between interfacial quality and synthetic flexibility. In situ growth typically affords stronger interfacial coupling owing to direct nucleation of the MOF on the 2D substrate. In contrast, ex situ methods offer greater freedom in material selection, processing conditions, and architectural design, while preserving the intrinsic properties of each component and enabling scalable fabrication. Consequently, the optimal strategy depends on the targeted application, with in situ approaches favored for interface‐dominated functionalities and ex situ routes better suited for modular, multicomponent, and large‐scale hybrid systems.

### 2.2 2D Materials Confined within MOFs (MOFs Encapsulated 2D Materials)

The encapsulation or confinement of 2D materials within MOFs has emerged as a powerful strategy to engineer hybrid architectures that combine the advantages of both materials while overcoming their individual limitations. In such systems, 2D materials are either intercalated between MOF layers or encapsulated within the MOF's porous matrix, resulting in unique microenvironments and enhanced physicochemical properties. This approach is particularly attractive for applications in electrocatalysis and energy storage, where the synergy between the high surface area and tunable porosity of MOFs and the exceptional electronic, mechanical, and chemical features of 2D materials can be fully exploited.

#### 2.2.1 Interlayer Encapsulation and Confinement

Interlayer encapsulation involves the insertion of 2D nanosheets such as GO, TMDs, or MXenes between the layers of a MOF, or the growth of MOFs around pre‐formed 2D materials. This process creates hybrid structures where the 2D material is confined within the MOF matrix, resulting in effective interfacial contact and the potential for novel functionality. The confined 2D material can act as a conductive bridge, mechanical reinforcement, or a platform for additional functionalization, while the MOF provides a protective and tunable environment that can stabilize reactive species or enhance selectivity. This design leverages the molecular selectivity and protective environment of the MOF, and its porous framework can prevent aggregation and oxidative degradation of 2D nanosheets, localize reactants around active 2D sites, and impose steric gating that enhances selectivity for size or shape‐sensitive reactions. The host‐guest interaction can also alter the electronic structure of the encapsulated 2D material via charge transfer or by modulating dielectric screening, producing changes in optical absorption, catalytic activity, or electron mobility that are not present in the physical mixture of components.

Encapsulation strategies often begin with dispersion of exfoliated 2D sheets in ligand/metal precursor solutions, followed by controlled MOF nucleation; alternatively, preformed MOF shells can be grown around 2D templates via stepwise ligand addition to produce uniform core‐shell geometries. Numerous studies have highlighted the potential of MOF‐encapsulated 2D material hybrids. For example, Qiao et al. [48] reported the encapsulation of ultrathin MoS_2_ nanosheets within a MOF‐199 matrix, resulting in a composite with enhanced electrocatalytic activity for HER. The MOF not only prevented the restacking of MoS_2_ but also provided a porous environment that facilitated mass transport and increased the accessibility of active sites. Another notable example is the development of MOF/GO membranes for water purification and gas separation [49]. The intercalation of GO into the MOF layers resulted in lamellar structures with tunable interlayer spacing, enabling fine control over molecule sieving and transport (Figure 3d). These membranes demonstrated high flux, selectivity, and mechanical strength, outperforming conventional polymer‐based membranes in several separation processes.

Although encapsulation and confinement strategies offer notable advantages, they also present several challenges. Restricted pore accessibility and diffusion limitations may arise when 2D materials partially block MOF channels, while nonuniform encapsulation or excessive MOF growth can hinder active‐site exposure and electron transport. Interfacial instability under electrochemical or chemical environments and difficulties in achieving scalable, reproducible architectures further limit practical application. These issues can be mitigated by precise control of MOF nucleation kinetics, pore size, and linker engineering to preserve mass transport, and surface functionalization to strengthen host‐guest interactions. In addition, modular or stepwise assembly and mild post‐synthetic treatments can improve structural uniformity, stability, and scalability of encapsulated MOF‐2D material hybrids.

### 2.3 Layer‐by‐Layer Assembly and Heterojunction Stacking

The layer‐by‐layer assembly and heterojunction stacking of MOFs and 2D materials have emerged as highly versatile and controllable strategies for fabricating hybrid architectures with precise structural and functional properties. These methods allow for the rational design of interfaces, enabling the integration of complementary functionalities from both MOFs and 2D materials, and offer significant advantages in terms of alignment, exfoliation, and defect minimization.

#### 2.3.1 Layer‐by‐Layer Assembly

It is a bottom‐up approach wherein alternating layers of MOF nanosheets and other functional materials, such as 2D nanosheets, are sequentially deposited onto a substrate. This technique exploits various interactions, including ionic, hydrogen, and covalent bonding, to achieve strong interfacial adhesion and uniform stacking. In this approach, MOFs are sequentially grown on graphene or its derivatives by alternately depositing metal ions and organic linkers [50]. Typically, GO serves as the substrate, with the assembly carried out stepwise, either starting with the metal source or the linker. The process can be done entirely in one pot or through intermediate extraction, but it usually begins with reacting a graphene dispersion with a linker followed by the addition of a metal salt. This approach results in a highly ordered, often defect‐free, multilayered structure with tunable thickness, composition, and orientation.

A notable example is the successful layer‐by‐layer assembly of ZIF67‐L (a leaf‐like zeolitic imidazolate framework nanosheet) with poly(acrylic acid) (PAA) [51]. In this work, ZIF67‐L nanosheets were first functionalized with polyethylenimine (PEI) to impart a positive charge, enabling ionic bonding with the negatively charged PAA. The assembly process led to polymer composites with highly aligned exfoliated MOF nanosheets. The X‐ray diffraction (XRD) patterns revealed in‐plane orientation of ZIF‐67, indicating a high degree of alignment which is considered to be a hallmark of the layer‐by‐layer technique. It has also been applied to fabricate membranes and films from 2D nanosheets with matching physical properties [52].

**FIGURE 4:**
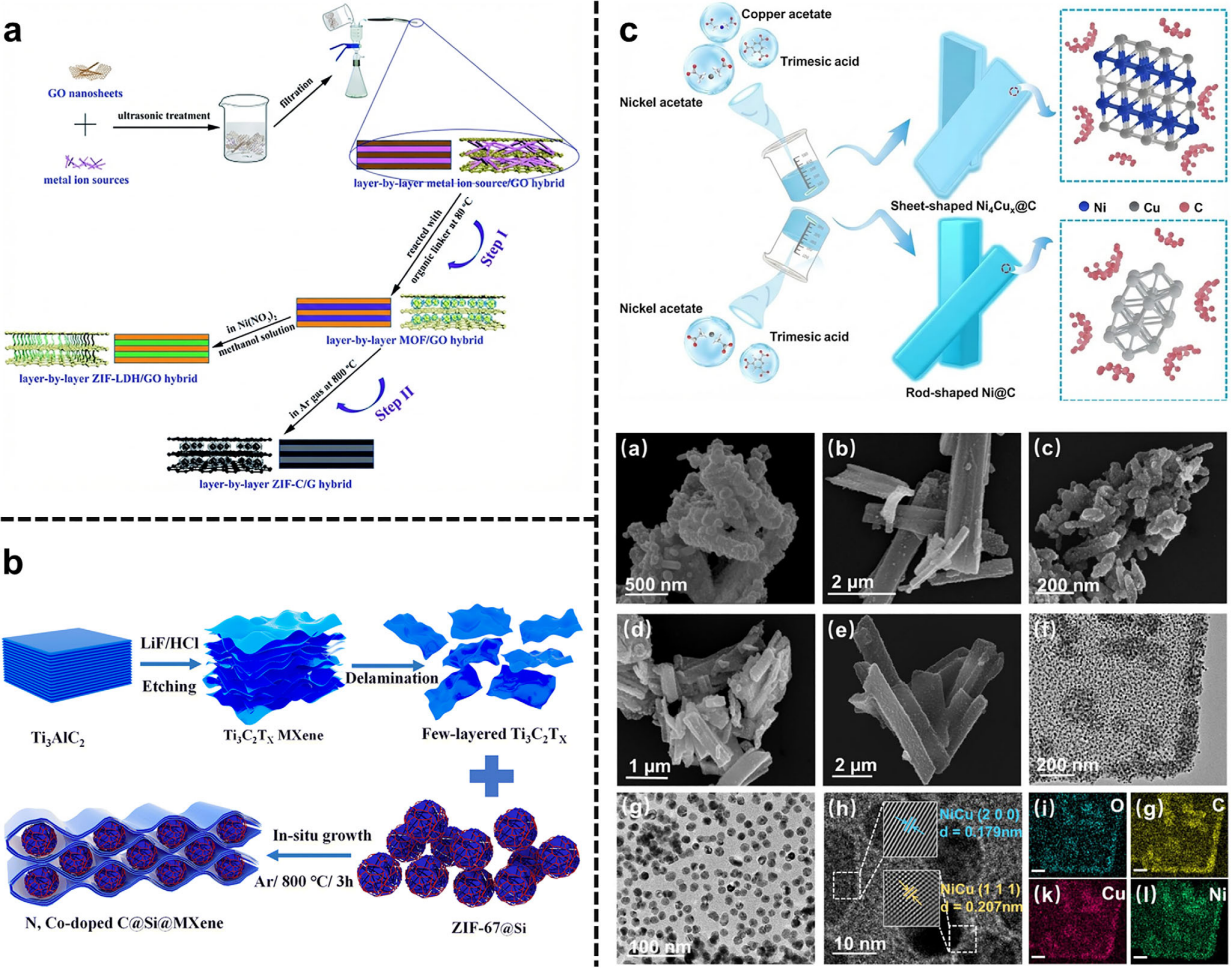
(a) Schematic representation of the synthesis process to layer‐by‐layer MOF/GO hybrid films and their derived supercapacitor electrode materials. Reproduced with permission [50]. Copyright 2017, The Royal Society of Chemistry. (b) Schematic illustration of the preparation of N, Co‐doped C@Si@MXene. Reproduced with permission [66]. Copyright 2024, The Royal Society of Chemistry. (c) Schematic illustration of the synthesis process and morphological characterization of the Ni_4_Cu_x_@C nanosheets. Reproduced with permission [67]. Copyright 2024, Elsevier.

Layer‐by‐layer assembly is not limited to MOF‐polymer and 2D/2D heterostructure systems. Layer‐by‐layer assembly has also been applied in developing hybrid electrodes for energy storage, where MOF‐199 was sequentially deposited onto GO through the layer‐by‐layer process [53], yielding a hybrid anode material for lithium‐ion batteries. The resulting composite exhibited high porosity, low charge resistance, and a stable cyclic performance, demonstrating the potential of layer‐by‐layer‐assembled MOF‐2D material hybrids in electrochemical applications. Furthermore, Yu et al. demonstrated a general layer‐by‐layer strategy for synthesizing graphene@MOF hybrids using ZIF‐67, HKUST‐1, and ZIF‐8 (Figure 4a) [50].

#### 2.3.2 Heterojunction Stacking

The heterojunction stacking strategy provides an effective route to integrate MOFs with 2D materials for advanced functional applications. In this approach, layered MOFs are directly stacked onto 2D substrates such as graphene, TMDs, or MXenes, forming interfacial junctions. Unlike uniform coatings obtained by layer‐by‐layer assembly, heterojunction stacking emphasizes the creation of strong interfacial coupling, where synergistic effects between the porous, redox‐active MOFs and the conductive 2D layers significantly enhance charge transfer, ion transport, and structural stability. Such interfacial engineering not only optimizes electrical conductivity but also provides abundant active sites accessible through both the MOF's intrinsic porosity and the high surface area of 2D sheets. It extends the layer‐by‐layer concept to the integration of different 2D materials or the combination of MOFs with other layered structures to form multi‐component heterostructures. These heterojunctions can be engineered to create unique electronic, optical, or catalytic properties by exploiting interfacial charge transfer, band alignment, or synergistic effects. For instance, stacking MOF nanosheets with 2D TMDs or black phosphorus can yield heterostructures with enhanced photocatalytic or electrocatalytic activity, as the interface promotes efficient separation and transport of charge carriers [35].

Layer‐by‐layer assembly and heterojunction stacking provide powerful platforms for the development of MOF‐2D material hybrid architectures with tailored properties. These methods enable precise control over structure and composition, facilitating the development of advanced materials for separation, catalysis, energy storage, and sensing applications. Despite their advantages, challenges remain in achieving perfect alignment, minimizing defects, and scaling up. Imperfections in nanosheet alignment can lead to defects such as mud‐crack‐like patterns, which may affect membrane performance [12]. These limitations can be addressed by enhancing interfacial interactions via surface functionalization or linker engineering to promote robust and uniform adhesion between layers. Moreover, the use of controlled assembly methods, including vacuum‐assisted filtration, electrophoretic deposition, and shear‐driven alignment, can improve nanosheet orientation and packing. Scalable solution‐based or roll‐to‐roll fabrication approaches further provide viable routes toward large‐area architectures with reduced defect densities.

### 2.4 MOFs Derived 2D Materials

The transformation of MOFs into 2D materials represents a cutting‐edge direction in materials chemistry, offering a route to synthesize ultrathin, highly functional nanosheets with properties inherited from and surpassing those of their MOF precursors [54, 55]. A distinct and rapidly growing route to MOF‐2D materials synergy is to convert MOFs into functional 2D materials by thermal or chemical transformation. MOFs serve as excellent molecular precursors because their metal nodes and organic ligands are already atomically dispersed and compositionally tunable. Controlling pyrolysis conditions, atmosphere, and the presence of salt or confinement templates can result in ultrathin carbonaceous nanosheets, N‐doped porous carbons with atomically dispersed metal sites, metal or metal‐oxide nanosheets, and bimetallic alloy sheets that retain the original MOF morphology.

#### 2.4.1 MOFs Derived Nanosheets via Pyrolysis/Etching Strategy

Pyrolysis and etching are the most widely utilized techniques for converting MOFs into 2D materials. Pyrolysis involves the thermal decomposition of MOFs under inert or reactive atmospheres, leading to the formation of carbonaceous materials, often doped with heteroatoms and embedded with metal or metal oxide nanoparticles. This process preserves the high surface area, hierarchical porosity, and sometimes the layered morphology of the parent MOF, while imparting electrical conductivity and catalytic activity. For example, the pyrolysis of ZIF‐67/GO hybrids produces core‐shell nanoparticles of Co@CoO encased in thin layers of nitrogen‐doped graphitic carbon (N‐GC), which exhibit exceptional bifunctional electrocatalytic activity for both OER and ORR [56].

The etching strategy typically involves selectively removing certain components of a MOF composite, often after partial carbonization, to generate ultrathin sheets with exposed active sites and tunable porosity. Acid or base etching can be used to remove metal nanoparticles or to open up the carbon matrix, enhancing accessibility for reactants and improving performance in applications such as supercapacitors or catalysis [57]. For instance, MOF‐derived carbon nanosheets etched with acid have demonstrated increased surface area and improved ion transport, making them suitable for electrode materials.

In addition to MOF‐derived 2D materials obtained via pyrolysis or etching, it is important to note that MOFs themselves can exist as intrinsically 2D architectures, commonly referred to as MOF nanosheets, MOF layers, or metal‐organic nanosheets (MONs) [58, 59]. These materials are composed of single‐ or few‐layer coordination networks formed through anisotropic crystal growth or post‐synthetic exfoliation of layered MOFs. MONs offer atomically thin thicknesses, high density of exposed metal nodes and organic linkers, and well‐defined porosity without requiring high‐temperature treatment or compositional transformation. Owing to their structural integrity and tunable chemistry, MONs have demonstrated promising performance in catalysis, sensing, membrane separation, and electrochemical energy storage [60, 61, 62]. Although MONs are not hybrid or derivative materials, their existence underscores the broader versatility of MOFs as a platform for 2D material design and provides an important conceptual bridge between pristine MOF nanosheets and MOF‐derived 2D carbonaceous or inorganic materials.

A notable advantage of these methods is the ability to tailor the chemical composition and structure of the resulting 2D materials by varying the MOF precursor, pyrolysis temperature, atmosphere, and etching conditions. For example, the use of bimetallic or multi‐metallic MOFs as precursors allows for the development of bimetallic or alloyed nanosheets, which can exhibit synergistic effects in electrocatalysis and energy storage applications [63]. In a broader context, this tunability is inherent not only to MOF‐derived systems but also to intrinsically MONs, in which compositional control, coordination environment, and layer thickness can be modulated at the molecular level without structural transformation. Together, pristine MONs and MOF‐derived 2D materials highlight the versatility of MOFs as a unified platform for engineering functional 2D architectures across a wide spectrum of electrochemical and catalytic applications.

Despite their effectiveness, both pyrolysis‐, etching‐derived 2D materials and pristine MONs face inherent limitations. Pyrolysis often leads to partial collapse of the MOF framework, agglomeration of metal nanoparticles, and loss of precise structural definition, while high temperatures can obscure active‐site uniformity and reduce compositional control [64]. Etching strategies may introduce structural defects, pore blockage, or chemical instability due to harsh acidic or basic conditions [65]. Similarly, MONs can suffer from limited chemical stability, restacking of nanosheets, and challenges in large‐scale, defect‐free synthesis. These drawbacks can be mitigated through rational precursor design, such as using robust or layered MOFs, controlled pyrolysis under optimized atmospheres, soft or selective etching protocols, and post‐synthetic surface functionalization. In addition, interlayer spacing engineering, heterostructure formation, and scalable bottom‐up synthesis approaches can help suppress restacking, enhance stability, and improve the practical applicability of MOF‐based 2D materials.

#### 2.4.2 MOF‐Templated 2D Carbon, M‐N‐C, or Bimetallic Sheets

The templating capability of MOFs is leveraged to direct the synthesis of 2D carbon materials, metal‐nitrogen‐carbon (M‐N‐C) catalysts, and bimetallic sheets with atomic precision. MOF‐templated synthesis involves using the highly ordered and tunable framework of a MOF as a scaffold for the formation of 2D materials during pyrolysis or chemical transformation. By incorporating different metals or heteroatoms into the MOF structure, researchers can engineer the distribution and nature of active sites in the final 2D product.

For example, the pyrolysis of ZIF‐8 or ZIF‐67(which contain Zn or Co ions, respectively) leads to the formation of nitrogen‐doped carbon nanosheets with atomically dispersed metal sites (Zn‐N‐C or Co‐N‐C) (Figure 4b) [66]. These materials have shown exceptional electrocatalytic activity for OER, HER, and CO_2_ reduction reactions, attributed to the high density of accessible active sites and the conductive carbon matrix. Similarly, bimetallic or multi‐metallic MOFs can be transformed into homogeneous bimetallic alloys or mixed oxide nanosheets that benefit from electronic modulation and synergistic binding to reaction intermediates; alloying often tunes the d‐band center and adsorption energetics, improving catalytic selectivity and activity (Figure 4c) [67]. Etching and displacement reactions expand this portfolio beyond carbonization. Selective chemical removal of ligands or sacrificial metals can transform MOF architectures into ultrathin oxide or chalcogenide sheets with retained porosity and high surface exposure of active sites. These MOF‐derived 2D materials therefore bridge the molecular precision of coordination chemistry with the robust electronic properties of inorganic 2D materials, creating highly active, stable materials.

MOF‐templated methods are effective but encounter several limitations that restrict their full potential. High‐temperature pyrolysis can cause framework collapse and metal aggregation, while volatile metals (e.g., Zn) may evaporate, reducing metal utilization [68]. Furthermore, defects, nonuniform thickness, and precise control over sheet size and scalable synthesis remain challenging. These issues can be mitigated by controlled MOF precursor design, confined pyrolysis to suppress metal sintering, and mild, selective etching strategies. Post‐synthetic treatments and low‐temperature transformation routes further help preserve structural integrity and active‐site accessibility.

Across all four architectural categories, several recurring design principles guide successful materials development. First, interfacial chemistry matters: whether one grows a MOF on a 2D substrate, encapsulates a 2D sheet, stacks layers in a layer‐by‐layer sequence, or converts a MOF into a 2D carbon, the nature and strength of chemical bonds at interfaces and the local electronic coupling influence the charge transfer kinetics and mechanical stability. Second, balancing porosity and transport is crucial. Low porosity or overly thick MOF shells limit mass transport and reaction rates, whereas excessive porosity can compromise selectivity or stability; hierarchical porosity and thin shell designs are common solutions. Third, morphology control at the nanoscale is essential for exposing active sites and minimizing transport lengths. Notably, ultrathin sheets, oriented nanosheet arrays, and spatially separated active centers (e.g., single atoms in M‐N‐C) are repeatedly associated with high performance. Fourth, process compatibility and scalability remain practical constraints. Many high‐performance hybrid architectures are synthesized under laboratory conditions that are difficult to scale (mechanical stamping of exfoliated layers, delicate layer‐by‐layer cycles, or precise pyrolysis under non‐standard atmospheres), and translating these methods to large‐area membranes or commercially relevant electrodes will require process simplification or new manufacturing strategies. Finally, stability under operating conditions (chemical, thermal and electrochemical) is a persistent concern for many MOF‐containing systems. Strategies like encapsulation, conductive supports and MOF‐to‐carbon conversion are common approaches used to enhance lifetime and durability. Collectively, these design principles emphasize a systems perspective. Hybrid materials must be optimized not only at the molecular level but also in terms of mesoscale architecture and device integration to realize practical performance gains.

In summary, the four architectural categories discussed (MOF growth over 2D substrates, 2D materials confined within MOFs, layer‐by‐layer assembly, and MOF‐derived 2D materials) collectively provide a powerful and versatile toolbox for constructing hybrid systems. To facilitate a direct comparison, the typical processing steps, interfacial coupling nature, and key advantages and limitations of these strategies are systematically summarized in Table 1. However, a critical evaluation reveals a distinct dichotomy between structural precision and practical scalability. While layer‐by‐layer assembly serves as an ideal platform for elucidating fundamental structure‐property relationships due to its atomic‐level control, its application is often constrained by processing complexity. Conversely, in situ growth and MOF‐derived strategies are emerging as the most viable pathways for robust, high‐performance energy devices, offering seamless interfacial integration essential for long‐term durability. The choice of architecture is not arbitrary; it is a deliberate decision made to precisely control the fundamental physics and chemistry at the dimensional interface. Having established this architectural framework, we will now delve into the core structure‐property relationships that govern the performance of these hybrids, linking nanoscale design to macroscopic function.

**TABLE 1:** Summary of fabrication strategies for MOF‐2D hybrid architectures.

Strategy	Typical process	Dominant interfacial interaction	Coupling nature	Advantages	Limitations	Typical architectures	Representative examples (References)
In situ growth	Surface activation → seeding & nucleation on 2D nanosheets in precursor solution	Coordination bonding; hydrogen bonding	Strong chemical coupling (direct nucleation)	Intimate contact; uniform coverage; tunable orientation	Thickness sensitivity; synthesis condition dependence; potential pore blockage	Core‐shell / MOF‐on‐sheet; oriented arrays	[37, 39, 40, 41, 42]
Ex situ growth	Synthesis of MOF NPs → mixing & assembly → electrostatic binding → hybrid consolidation	Electrostatic interactions; van der waals forces	Weak physical coupling (surface adsorption)	Scalable process; universal applicability; preserves intrinsic crystallinity	High interfacial resistance; weaker coupling; nonuniform dispersion (aggregation)	Randomly mixed composites	[45, 46, 47]
Interlayer encapsulation and confinement	Dispersion → directed intercalation of ions/linkers → interlayer confinement or pore encapsulation	Host‐guest interaction; spatial confinement	Spatial / host‐guest coupling (confinement)	Anti‐restacking stability; molecular sieving; enhanced chemical stability	Diffusion limitations; restricted pore accessibility; loading control difficulty	Sandwich / lamellar; core‐shell	[48, 49]
Layer‐by‐Layer assembly and heterojunction stacking	Alternating deposition of precursors/Sheets → interfacial assembly → controlled stacking	Ionic / covalent bonding; *π*–*π* stacking	Tunable strong coupling (Electrostatic/Covalent)	High precision (Atomic‐level); ordered architecture; defect minimization	Processing complexity (Time‐consuming); scalability issues; potential alignment defects	Multilayer films; ordered superlattices	[50, 51, 52, 53]
MOFs derived 2D materials	MOF‐2D hybrid precursors → pyrolysis / carbonization → etching / transformation	Inherited template structure (transformation)	Intrinsic atomic coupling (structural inheritance)	High conductivity; robust active phases (e.g., M‐N‐C); hierarchical porosity	Complex phases; risk of framework collapse; metal agglomeration	Carbon nanosheets / M‐N‐C; nanomeshes	[56, 66, 67]

## 3 Structure‐Property Correlation in MOF‐2D Hybrids

**FIGURE 5:**
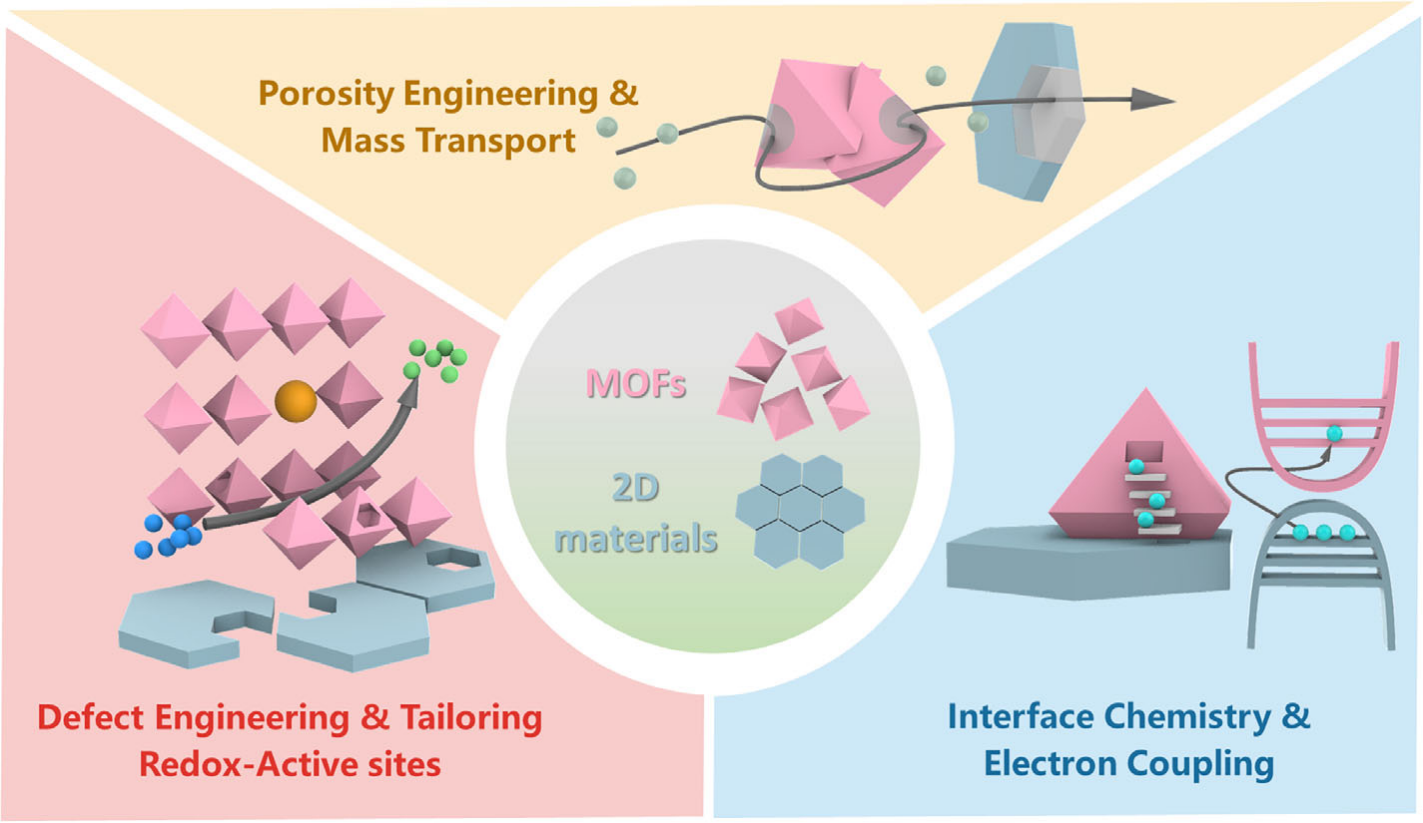
Schematic illustration of different types of structure–property correlation in MOF–2D Hybrids.

Establishing rigorous structure‐property correlations is central to the rational design of MOF‐2D material hybrids. In these architectures, pore size distribution and connectivity from the MOF framework couple with the anisotropic, layer‐controlled transport pathways and interfacial chemistry of 2D hosts (e.g., MXene, graphene/GO, and MoS_2_). Key descriptors‐accessible porosity and percolation, interlayer spacing and stacking registry, defect density and interfacial binding, and platelet orientation‐govern mass and charge transport, selectivity, and mechanical/thermal stability at the device level. Throughout this section, we emphasize experimentally tractable descriptors and qualitative trends to illustrate how dimensional coupling creates transport channels and selectivity unattainable in the individual components (Figure 5). These same structural levers underpin the performance gains discussed in Section 4.1, where interfacial chemistry and accessible channels steer reactant delivery and product release in electrocatalysis, and in Section 4.2, where continuous electron pathways and wettable, hierarchical pores enable high‐loading, high‐rate batteries and supercapacitors.

### 3.1 Porosity Engineering and Mass Transport

Porosity engineering in MOF‐2D hybrids governs mass transport by co‐designing pore hierarchy and tortuosity at the MOF/2D interface. MOF micropores (≤2 nm) impart size/chemical selectivity and surface diffusion, while interlayer slits, wrinkles, and voids in the 2D scaffold provide meso/macroporous highways that shorten path lengths and relieve concentration gradients [12, 20]. Effective diffusivity is determined less by total pore volume than by accessibility, connectivity, and orientation, which are set by interfacial packing and stacking order: well‐registered, uniformly spaced laminates yield straight, low‐τ channels, whereas turbostratic disorder and restacking introduce lateral detours and dead‐end pores. Practically, hierarchy and tortuosity are tuned via MOF crystallite size and integration mode (intergrowth vs particulate deposition), regulation of sheet spacing with intercalants or covalent/ionic cross‐links, and the use of steric pillars or controlled wrinkling to stabilize anisotropic, percolating pathways. Such control sustains high‐rate function‐mitigating concentration polarization and enables gas/product egress in electrocatalysis, and ensures electrolyte wetting and rapid ion access in thick supercapacitor electrodes, delivering predictable, scalable transport in MOF‐2D architectures.

In the interdiffusion‐grown Ti_3_C_2_T_x_‐CoBDC laminate, porosity and transport are co‐designed at the MOF/MXene interface [69]. Surface ‐OH/‐F terminations on Ti_3_C_2_T_x_ electrostatically preconcentrate Co^2+^, which then coordinates with BDC while the nanosheets traverse a solvent gradient, yielding a conformal CoBDC coating on both basal planes. This prevents MOF restacking, preserves MOF‐intrinsic microporosity for selective adsorption/surface diffusion, and introduces hydrophilic, slit‐like pathways within the MXene galleries for rapid electrolyte infiltration. The seamless junction converts discrete particles into a continuous, low‐tortuosity network. MOF domains supply short through‐thickness diffusion paths, while the MXene scaffold provides percolating ion/electron corridors and minimizes dead‐end pores relative to physical mixtures. Thus, hierarchical micro‐meso connectivity at a chemically welded interface is translated directly into predictable mass flux across the hybrid film.

Mahato et al. construct a heteronanoarchitecture that exemplifies interface‐directed porosity and transport [70]. By growing a nanoporous amorphous MOF within the interlayer slits of Ti_3_C_2_T_x_, the laminate increases the d‐spacing and establishes a micro‐mesopore spectrum (∼1.4–7.7 nm), converting restacking‐prone galleries into continuous, hydrophilic ion highways. At the same time, hydrogen‐bonding, coordination, and ester linkages at the interface electronically stabilize the MXene terminations, preserving metallic pathways while enabling rapid, reliable mass transport. The hybrid boosts accessible area (∼260 m^2^ · g^−1^) yet preserves metallic pathways, delivering EDLC‐type kinetics with high capacitance (∼236 F · g^−1^ at 100 mV · s^−1^) and exceptional open‐air durability (∼99% retention after 50 000 cycles). This interfacial design, which combines hierarchical porosity with low tortuosity, directly links pore architecture to reliable, high‐rate mass transport in MOF‐2D systems.

In MOF‐derived graphene nanomesh, porosity is programmed by a two‐step dimensional‐reduction of 2D ZIF‐L via molten‐salt exfoliation, which unravels the layered precursor into ∼1.3 nm sheets riddled with in‐plane nanopores [71]. Concomitantly, the (002) spacing expands, edge defects and N dopants proliferate, and a hierarchical micro–mesoporous network forms. This architecture shortens intra‐/cross‐plane diffusion paths, creates continuous low‐tortuosity ion/gas corridors, and suppresses restacking, translating precursor geometry into predictable mass flux across ultrathin carbon laminates.

### 3.2 Interface Chemistry and Electron Coupling

#### 3.2.1 Role of Interfacial Orbital Hybridization

The substrate plays a critical role in determining the properties of Fe‐TCNQ 2D MOFs by controlling both the coordination geometry and the d‐level alignment at the interface [72]. This, in turn, tunes the electronic coupling. On graphene, the network adopts an intrinsically nonplanar structure, which stabilizes quasi‐tetrahedral Fe coordination sites. In contrast, on Au(111), stronger van der Waals interactions planarize the molecular layer. This pulls the Fe atoms approximately 1 Å closer to the metal surface, fundamentally altering the orbital overlap at the junction. These distinct structural and electrostatic environments lead to significant shifts in the Fe orbital occupancy and energy. Structural effects alone account for an energy shift of 0.2‐0.9 eV. Work‐function alignment, modified by the surface dipole push‐back effect, contributes an additional 0.4–0.5 eV. Combined, these factors result in a total difference of ∼1.4 eV in the center (and ∼0.8 eV in the d‐band center) between the tilted graphene‐supported structure and the planarized Au‐supported one. The consequences for interfacial reactivity are stark. On graphene, TCNQ exhibits a chemical‐bonding adsorption mode and induces a large work‐function increase of over 0.5 eV, consistent with its high thermal stability. On Au (111), however, the interaction is characterized by weak physisorption and a minimal work‐function change of only ∼0.1 eV.

#### 3.2.2 Built‐In Electric Fields/Heterojunction Effects

**FIGURE 6:**
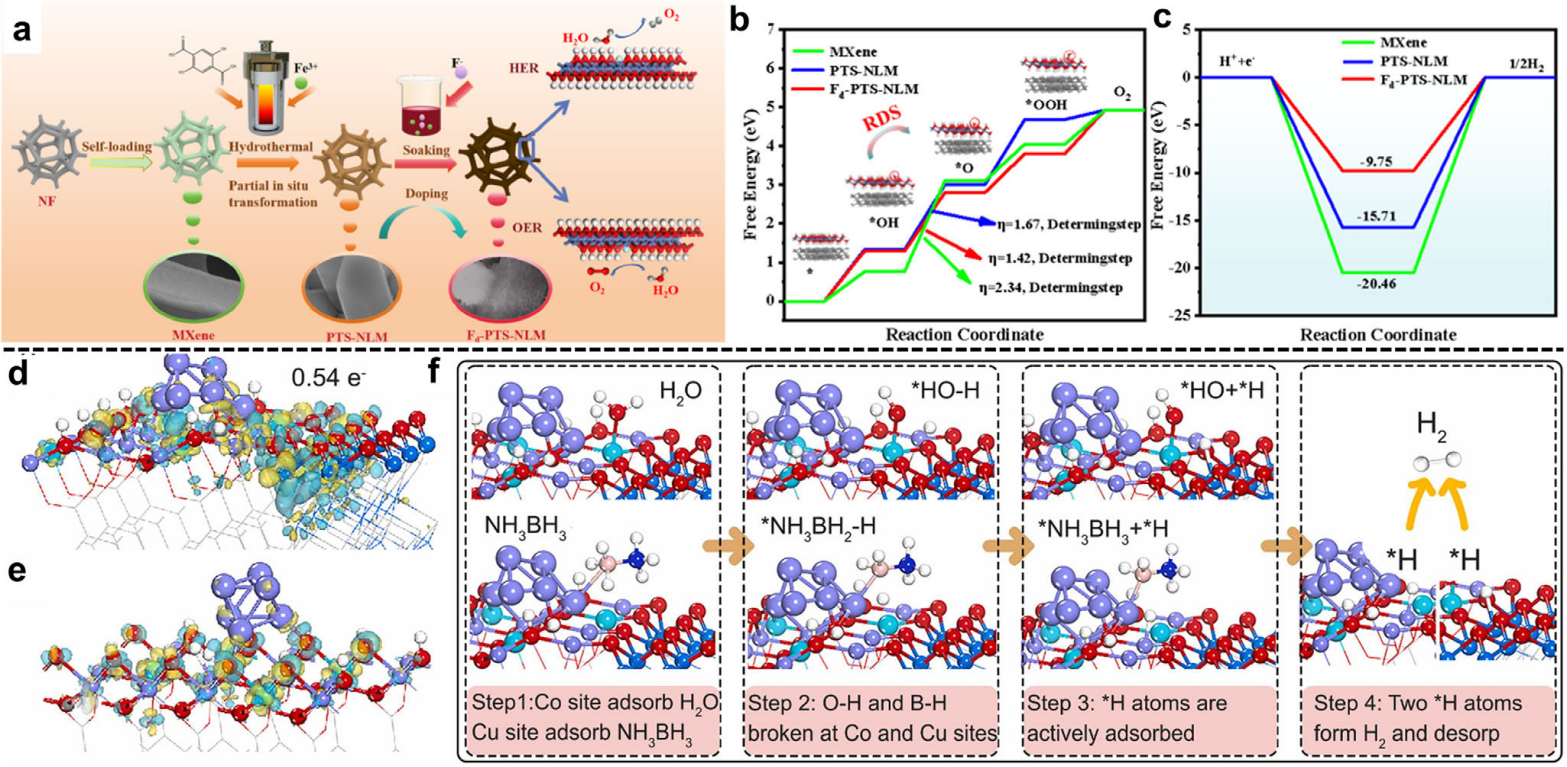
(a) Schematic illustration of Fd‐PTS‐NLM. (b) Computational free energy diagrams of Fd‐PTS‐NLM and comparative samples for OER intermediates. (c) Calculated free energy diagram of H adsorption of Fd‐PTS‐NLM and comparative samples. Reproduced with permission [73]. Copyright 2025, American Chemical Society. (d) CuCo‐MOF/MX and (e) CuCo‐MOF (yellow: electron accumulation, cyan: electron depletion). (f) Simulation of NH_3_BH_3_ hydrolysis pathways on CuCo‐MOF/MX catalyst. Cu, Co, Ti, B, N, O, and H atoms are shown in royal blue, cyan, navy, pink, blue, red, and white, respectively. Reproduced with permission [74]. Copyright 2024, Elsevier.

In a MXene/NiFe‐layered double hydroxides (LDHs)@MOF heterostructure, a partial in situ transformation of NiFe‐MOF‐74 to NiFe‐LDH on Ti_3_C_2_T_x_ creates an intimate LDH/MOF shell on a conductive MXene core; subsequent F‐doping introduces anion‐vacancy‐rich sites and drives interfacial charge redistribution (Figure 6a) [73]. XPS (X‐ray photoelectron spectroscopy) shifts and charge‐difference maps reveal electron accumulation/depletion across the junction, establishing a built‐in electric field that lowers charge‐transfer resistance and tunes the d‐band center. DFT (Density functional theory) attributes the kinetic gains to a reduced ∗O→OOH barrier (OER) and near‐optimal ΔG_H_ (HER), while in situ Raman identifies NiOOH/FeOOH as the true OER species, as illustrated in Figure 6b,c. Performance mirrors the mechanism: η_10_ = 150 mV (HER), 171 mV (OER), and 1.50 V for overall splitting with ∼100% Faradaic efficiency.

In a sandwich MOF‐2D catalyst, coupling Cu/CuCo‐MOF with Ti_3_C_2_T*_x_* MXene creates double heterojunctions in which an interfacial Cu‐O‐Ti bridge drives charge transfer from Ti^δ^^+^ to Cu^2+^ and establishes a built‐in electrostatic bias across the junction [74]. XPS shows a ∼0.3 eV negative shift of Cu^2+^ 2p and positive shifts of Ti‐O/F, while differential charge‐density maps resolve electron accumulation/depletion at the interface, direct signatures of interfacial polarization. Density‐of‐states analysis reveals d‐band center tuning (−1.619→−1.491eV), correlating with lower barriers for H_2_O and NH_3_BH_3_ activation (down to 0.375 and 0.684 eV) and elongated O‐H/B‐H bonds on the optimized sites, as exhibited in Figure 6d–f. These field‐assisted, polarity‐matched junctions enhance electron/adsorbate coupling and deliver high hydrogen‐release kinetics (TOF up to 82.3 min^−1^) with robust cycling stability.

Beyond simple physical contact, the electronic coupling at the MOF‐2D interface can be fundamentally understood through Fermi level alignment and orbital hybridization models. When a MOF is integrated with a conductive 2D material, the difference in their work functions drives electron flow until equilibrium is reached, establishing a built‐in electric field at the interface. This field not only facilitates directional charge transfer but also induces band bending, thereby optimizing the adsorption energetics of reaction intermediates. Furthermore, d‐band center theory provides a critical descriptor for understanding these interactions. The coupling with 2D substrates can shift the d‐band center of the metal nodes in MOFs relative to the Fermi level. An upward shift typically strengthens the binding with adsorbates (favorable for activating inert molecules), while a downward shift weakens it (facilitating product desorption). Thus, interface engineering is essentially the precise modulation of these electronic parameters to break the scaling relations of catalysis.

### 3.3 Defect Engineering and Tailoring Redox‐Active Sites

#### 3.3.1 Defect Sites in MOFs (Missing‐Linker/Cluster) and 2D Materials (Vacancies, Edges)

Defect engineering and redox‐site tailoring can be achieved by converting shape‐controlled MOF precursors into ultrathin, N‐doped graphene nanomesh. Building on Zn–ZIF‐L nanoleaves, Wei et al. thermally exfoliate the precursor at 800°C to yield an ∼1.3 nm‐thick N‐doped graphene nanomesh (NGM‐800) with hierarchical micro–meso/macroporosity and very high surface area (∼1329.5 m^2^ g^−1^) [71]. This architecture concentrates edge defects and heteroatom‐doped functionalities that act as ORR redox‐active centers, while expanded interlayer spacing suppresses restacking and lowers tortuosity, ensuring rapid ion/gas access to active sites. The synergistic coupling of abundant, accessible defect/N sites with fast mass transport relieves concentration gradients and delivers markedly enhanced ORR kinetics, sustained even in acidic electrolytes.

#### 3.3.2 Interfacial Defect Propagation or Passivation

In 2014, Huang et al. prepared the bicontinuous ZIF‐8@GO membrane through layer‐by‐layer deposition, using GO to selectively seal grain‐boundary gaps between ZIF‐8 crystallites via capillary action and covalent bonding, forcing permeation through the MOF's intrinsic micropores. This interfacial “healing” suppresses non‐selective leak paths and even constrains the ZIF‐8 lattice flexibility, thereby preventing defect propagation across the MOF/GO interface and delivering sharply enhanced H_2_ selectivity in hydrogen separation [75].

In 2021, Covalent amide linkages between carboxylated graphene (graphene acid) and UiO‐66‐NH_2_ create a chemically welded MOF/graphene interface that stabilizes stacking, reduces interfacial voids, and builds an interconnected conductive/porous network (micropores from UiO‐66‐NH_2_; mesoporosity from GA interlayers) [76]. This passivates interfacial defects and yields low‐tortuosity transport with improved rate capability and cycling stability, an explicit demonstration that strong interfacial bonding arrests defect transmission and translates structural integrity into predictable mass/charge flux. A similar strategy can be found in UiO‐66‐NH_2_/reduced graphene oxide (rGO) heterostructures [77], covalent bonding (amide formation) between MOF amino groups and rGO is verified by XPS/FTIR (Fourier‐transform infrared spectroscopy) and produces intimate contact that reduces interfacial carrier‐transfer resistance, establishes an internal electric field for directional charge separation, and strengthens CO_2_ adsorption/activation. Compared with physically mixed or non‐covalent counterparts, the chemically bonded interface suppresses interfacial trap states (a form of defect passivation), accelerating charge and reactant/product transport during CO_2_ photoreduction.

#### 3.3.3 Use of Heteroatoms Introduced via 2D Substrates to Modulate MOF Electronic Environment

At MOF‐2D interfaces, heteroatom functionalities native to the 2D substrate act as “soft ligands” and electronic modulators that reshape node coordination, band alignment, and transport [78]. For N‐doped graphene oxide (NGO) scaffolds, pyridinic/pyrrolic N and O groups coordinate Ni centers during MOF‐74 growth (Raman Ni‐N band≈ 283 cm^−1^; XPS corroboration), yielding an intimately bonded NGO/Ni‐MOF hybrid with hierarchical micro–mesoporosity (type‐IV isotherm) that stabilizes accessible metal sites and lowers tortuosity. Upon sulfurization, this interfacial design is retained in NGO/Ni_7_S_6_, which exhibits smaller OER overpotential than RuO_2_ at 10 mA cm^−2^ (Tafel ≈ 45 mV dec^−1^) and competent alkaline HER (η_10_ ≈ 0.37 V), evidencing that 2D‐N coordination pre‐configures electronic structure while furnishing fast, percolating pathways.

Complementarily, P‐containing black phosphorus nanosheets donate electron density to Co^2+^/Fe^3+^ nodes when a 2D MOF‐Fe/Co layer is grown in situ on black phosphorus, as shown by binding‐energy shifts in Co 2p/Fe 2p and a ∼0.3 eV blue shift of P 2p, together with Mott–Schottky analysis indicating electron transfer from black phosphorus to MOF [79]. The chemically coupled black phosphorus @MOF heterojunction delivers an HER overpotential of 180 mV and OER overpotential ≈ 246 mV in 1 M KOH with a Tafel slope ∼56 mV dec^−1^ and a greatly enlarged double‐layer capacitance (C_dl_) (≈ 73 mF cm^−2^) relative to either component, linking substrate‐derived P chemistry to tuned MOF electronic environment and enlarged electrochemically accessible area.

#### 3.3.4 Generation of Redox‐Active Sites at the Interface of MOF‐2D Hybrids

**FIGURE 7:**
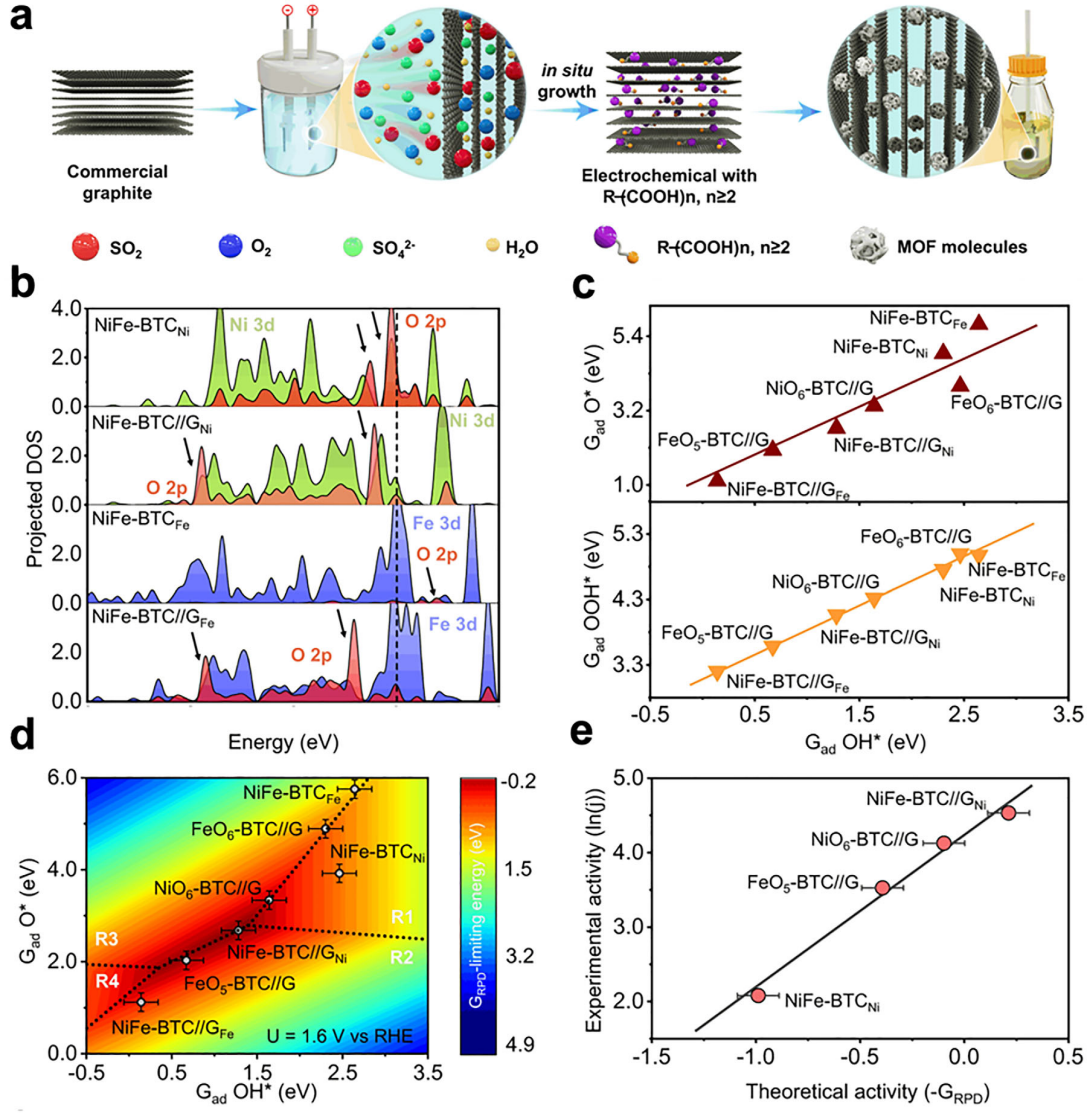
(a) Schematic illustration of the electrochemical synthesis process of NiFe‐MOF//G. (b) PDOS of Ni/Fe 3d and O 2p orbitals for NiFe‐BTC and Confined NiFe‐BTC. (c) Linear scaling relationship between adsorption energies of O*/OOH* vs. OH*. (d) Two‐dimensional activity map with O* and OH* as two independent descriptors. (e) The correlation between experimental activities (ln(*j*)) and theoretical ones (‐G_RPD_) derived from the G_RPD_‐limiting energies. Reproduced with permission [80]. Copyright 2022, Springer Nature.

At MOF‐2D interfaces, confinement, covalent anchoring, and electronic coupling can generate new redox‐active sites or reconfigure existing ones, while concurrently establishing low‐tortuosity pathways that synchronize ion and electron transport during catalysis. Lyu et al., confining poorly conductive MOFs (NiFe‐BTC) within graphene multilayers (NiFe‐BTC//G), create a MOF‐2D heterointerface that generates redox‐active sites and accelerates transport (Figure 7a) [80]. Electrochemical intercalation expands the graphitic galleries (d ≈ 0.668 nm) and yields a high surface area laminate (specific surface area = 762.7 m^2^ g^−1^). XAS/EXAFS resolve distorted NiO_6_‐FeO_5_ motifs and interfacial electron transfer with binding‐energy shifts. Complementary DFT analysis elucidates the confinement mechanism: the restricted space enhances electronic resonance between Ni/Fe 3d and O 2p orbitals (Figure 7b), thereby optimizing intermediate binding. Governed by linear scaling relationships (Figure 7c), these thermodynamic descriptors map the confined NiFe‐BTC//G active sites to the theoretical optimum region of the 2D activity landscape (Figure 7d), showing excellent consistency with the experimental activity trends (Figure 7e). The confined electrode delivers 106 mV at 10 mA cm^−2^ with a ∼55 mV dec^−1^ Tafel slope, C_dl_  ≈ 81.6 mF cm^−2^, R_ct_  ≈  0.46 Ω, and ≥150 h stability, providing direct evidence that nanoconfinement activates interfacial redox centers while enabling low‐tortuosity ion/electron pathways.

A powerful strategy for creating highly accessible redox‐active centers while accelerating charge transport is demonstrated by Lim et al. in their hybrid LIG@Cu_3_HHTP_2_ structure [81]. This material is prepared by the layer‐by‐layer growth of a 2D semiconducting MOF, Cu_3_HHTP_2_, onto laser‐induced graphene (LIG). The resulting architecture features a lung‐like macro/microporous network that provides a percolating conductive backbone from the LIG while exposing the MOF's open Cu nodes and catecholate/semiquinonate ligands. Evidence from XPS confirms a strong interfacial interaction, revealing mixed Cu(I)/Cu(II) states and enhanced O‐Cu components. This indicates efficient coordination and electron transfer at the junction, which increases the density and accessibility of the interfacial redox sites. Functionally, this coupled architecture shortens charge diffusion paths and enables rapid transduction. These synergistic benefits translate to exceptional room‐temperature gas sensing performance, with a rapid response‐recovery time of 16 s/15 s and a low detection limit of 0.168 ppb. This work exemplifies how engineered MOF‐graphene junctions can effectively generate and mobilize active redox centers for advanced applications.

Collectively, these structure‐property correlations governing porosity, interfacial chemistry, and defect sites are not merely academic concepts; they are the fundamental design principles that underpin performance. Crucially, we contend that while hierarchical porosity secures mass transport, it is the precise electronic coupling at the interface that dictates the intrinsic activity limits. Consequently, future design iterations must prioritize atomic‐level interfacial fidelity over mere surface area maximization. The ability to engineer porosity for mass transport, tune electronic coupling for charge transfer, and tailor defect sites for reactivity forms the scientific bedrock for tangible technological advancements. In the following section, we will demonstrate how these principles are applied to solve critical challenges in electrocatalysis and energy storage, showcasing state‐of‐the‐art examples where meticulous interface engineering has led to breakthrough performance.

## 4 Application

### 4.1 Electrocatalytic Application

Building on the structure‐property relationships outlined above, electrocatalysis provides a particularly stringent testing ground for MOF‐2D hybrid architectures, as catalytic performance is simultaneously dictated by mass transport, charge transfer, and interfacial reaction kinetics [82, 83, 84]. In electrochemical environments, the interplay between hierarchical porosity, interfacial electronic coupling, and defect chemistry directly governs reactant delivery, intermediate stabilization, and product removal at the active sites [85, 86, 87]. Consequently, the same structural descriptors like pore accessibility and connectivity, interlayer spacing and orientation, interfacial bonding, and defect distribution that define transport and stability at the materials level become decisive factors in determining electrocatalytic activity, selectivity, and durability.

In MOF‐2D hybrids, dimensional coupling enables reaction microenvironments that are difficult to realize in either component alone. Ordered MOF pores provide chemically selective channels and well‐defined coordination sites, while 2D hosts establish continuous electron pathways and anisotropic transport directions. Their integration creates coupled ionic‐electronic networks in which reactants are efficiently supplied through accessible channels, electrons are rapidly delivered to catalytic centers, and products are promptly evacuated, mitigating concentration polarization and kinetic bottlenecks. At the same time, interfacial orbital interactions, built‐in electric fields, and defect‐mediated charge redistribution further modulate the energetics of elementary reaction steps.

In this section, we discuss how these structure–property correlations translate into enhanced performance across key electrocatalytic reactions relevant to energy conversion, including HER, OER, ORR, and CO_2_RR (Table 2). Emphasis is placed on how rational control of porosity, interfacial chemistry, and defect sites enables MOF‐2D hybrids to overcome intrinsic kinetic and transport limitations, thereby establishing clear design principles for next‐generation electrocatalysts.

**TABLE 2:** Electrochemical catalysis performance comparison of different MOF‐2D materials hybrid for different reactions.

Materials	Synthesis method	Overpotential (vs. RHE)	Tafel slope	Key innovation	Limitation	Refs.
**Hydrogen evolution reaction**						
MFN	Ex situ method	52 mV	101 mV dec^−1^	Synergistic hybrid design	Poor stability	[93]
Mn‐MOF@rGO/Ti_3_C_2_T_x_	Ex situ hydrothermal method	121 mV	62 mV dec^−1^	Excellent stability	Insufficient mechanism study	[94]
MOF(Ni)‐GR(4%)	In situ solvothermal method	268 mV	108 mV dec^−1^	Convenient synthesis	Poor conductivity	[95]
Ni@N‐HCGHF	Ex situ method	95 mV	57 mV dec^−1^	More defect sites	Structure‐driven enhancement	[96]
Ni‐Ti_3_C_2_ MXene	Ex situ method	181 mV	56 mV dec^−1^	Low resistance	Unclear active phase	[97]
SGNC‐900	Thermal exfoliation method	32 mV	39 mV dec^−1^	Direct thermal exfoliation	Unclear single‐atom evidence	[98]
**Oxygen evolution reaction**						
Ti_3_C_2_T_x_‐CoBDC	In situ solvothermal method	410 mV	48.2 mV dec^−1^	Seamless interfacial coating	Limited long‐term stability	[69]
Co_2_Ni‐MOF/Ti_3_C_2_T_x_	Ex situ method	265 mV	51.7 mV dec^−1^	Conductive 2D/2D heterostructure	Hydroxide reconstruction	[47]
NiCoS/Ti_3_C_2_T_x_	In situ solvothermal method	365 mV	58.2 mV dec^−1^	Hierarchical porous hybrid	Irreversible structure transformation	[102]
CoFe MLDH/Ti_3_C_2_	In situ hydrothermal method	170 mV	31.5 mV dec^−1^	Ultralow overpotential	Substrate‐dependent metrics	[103]
TiNbC/MOF@SA‐H	In situ solvothermal method	185 mV	84 mV dec^−1^	3D conductive network	Limited mechanistic evidence	[104]
MGM_7_	In situ hydrothermal method	224 mV	123.4 mV dec^−1^	Kg‐scale nanosheets	High Tafel slope	[105]
Ni@N‐HCGHF	Ex situ method	260 mV	63 mV dec^−1^	More defect sites	Structure‐driven enhancement	[96]
ZIFCNDA	In situ method	510 mV	—	Suppressed nanoparticle aggregation	High overpotential	[106]

Materials	Synthesis method	Half‐wave potential	Onset potential	Key innovation	Limitation	Refs.
**Oxygen reduction reaction**						
B‐MNC	Ex situ method	—	0.7 V	hierarchical porosity	Low onset potential	[110]
Co‐SAs/N‐C/rGO	In situ solvothermal method	0.84 V	1.01 V	pH‐universal ORR activity	Low Co loading	[111]
NGM	Thermal exfoliation method	0.781 V	0.86 V	Defect‐enriched graphene edges	Inferior performance to Pt/C	[71]
PEI/PEDOT: PSS	Layer‐by‐layer method	—	—	Layer‐by‐layer assembly	Limited electrochemical metrics	[112]
PM‐900	Ex situ method	0.86 V	1.03 V	High graphitization	Moderate BET surface area	[113]
TR‐Z8‐2‐C	Ice‐template method	0.77 V	0.90 V	Ice‐templated self‐assembly	High resistance	[114]

Materials	Synthesis method	Faradaic efficiency	Relevant current density	Key innovation	Limitation	Refs.
**Carbon dioxide reduction reaction**						
In‐TCPP@MXene	In situ solvothermal method	94.0% (formate)	102.5 mA cm^−2^	High selectivity	Structure transformation	[115]
Cu GNC‐VL	In situ hydrothermal method	70.52% (ethanol)	10.4 mA cm^−2^	3D conductive network	Low current density	[116]
Cu‐THQ‐EFG	Ex situ method	31.7% (formate)	3 mA cm^−2^	Conductivity‐enhanced MOF	Low Faradaic efficiency	[117]

#### 4.1.1 Hydrogen Evolution Reaction (HER)

Hydrogen energy has been widely acknowledged as a highly promising candidate for sustainable energy systems owing to its high gravimetric energy density and the absence of carbon emissions during utilization [88, 89, 90]. Among the various hydrogen production strategies, electrocatalytic HER via water electrolysis has attracted significant attention due to its environmental benignity and potential for large‐scale deployment [84, 91, 92]. MOFs offer a versatile platform for HER by providing atomically defined metal coordination environments and accessible channels that facilitate reactant transport. When coupled with conductive 2D materials, these architectures overcome the intrinsic conductivity limitations of pristine MOFs, enabling efficient charge transport to catalytically active sites. Such MOF‐2D hybrids allow the electronic structure and hydrogen adsorption energetics to be jointly tuned, resulting in enhanced HER activity and stability.

**FIGURE 8:**
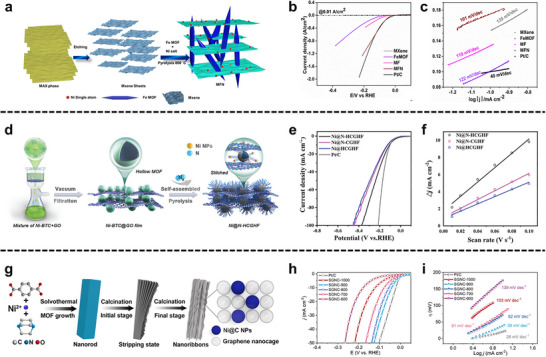
(a) Schematic illustration of the synthesis of MFN. (b) LSV curves of MXene, FeMOF, MF, MFN, and Pt/C. (c) Tafel slopes calculated from LSV curves in (b). Reproduced with permission [93]. Copyright 2024, The Royal Society of Chemistry. (d) Schematic illustration of the synthesis of Ni@N‐HCGHF. (e) LSV curves of Ni@N‐HCGHF, Ni@N‐CGHF, Ni@HCGHF, and Pt/C. (f) Double‐layer capacitance plots of Ni@N‐HCGHF, Ni@N‐CGHF, and Ni@HCGHF. Reproduced with permission [96]. Copyright 2020, Wiley‐VCH. (g) Schematic illustration of the synthesis of SGNCs. (h) LSV curves of SGNC‐1000, SGNC‐900, SGNC‐800, SGNC‐700, SGNC‐600, and Pt/C. (i) Tafel plots calculated from LSV curves in (h). Reproduced with permission [98]. Copyright 2020, American Chemical Society.

A sonochemical‐pyrolysis strategy was employed to construct a hybrid catalyst integrating Ni single atoms on Ti_3_C_2_T_x_ MXene sheets with Fe‐MOF, forming an ordered MXene‐MOF framework with atomically dispersed Ni sites coordinated to carbon vacancies (Figure 8a). The optimized hybrid delivered outstanding HER performance, requiring only 52 mV overpotential to reach 10 mA cm^−2^, with nearly 100% Faradaic efficiency and excellent durability over 27 h (Figure 8b,c). Electrochemical analyses revealed a low charge‐transfer resistance, while DFT calculations confirmed that Ni‐C coordination modulates the density of states and optimizes hydrogen adsorption, thereby accelerating proton reduction. These results highlight MXene‐MOF‐single atom hybrid systems as highly efficient and stable HER electrocatalysts. Despite the high initial HER performance, MXene‐MOF‐SAC systems may suffer from limited long‐term stability, as MXene supports are prone to oxidation/corrosion in acidic media and single‐atom sites can undergo coordination changes or aggregation under prolonged operation, leading to gradual activity decay [93].

One key strategy is to leverage the 2D substrate as a conductive support and electronic modulator. Hassan et al. synthesized Mn‐MOF@rGO/Ti_3_C_2_T_x_ composites via a hydrothermal strategy, effectively combining the porosity of Mn‐MOF, the conductivity of rGO, and the high surface area of MXene. In alkaline media, the optimized composite exhibited excellent HER performance, requiring only 121 mV overpotential at 10 mA cm^−2^ with a Tafel slope of 62 mV dec^−1^. The enhanced activity was attributed to the synergistic effects of rGO doping and MXene integration, which improved electron transfer and active‐site accessibility. These results highlight Mn‐MOF@rGO/Ti_3_C_2_T_x_ as a promising 2D composite electrocatalyst for efficient and sustainable hydrogen production. However, the mechanistic understanding remains insufficient, as the study provides limited insights into the true active phase, reaction pathway, and electronic interactions among Mn‐MOF, rGO, and MXene, leaving the origin of activity enhancement only partially resolved [94].

To further highlight the role of graphene in conductivity enhancement, Dao et al. prepared Ni‐MOF and graphene‐doped Ni‐MOF composites [MOF(Ni)‐GR(w%)] via a solvothermal route to boost HER activity. Structural analyses confirmed that graphene incorporation enhanced crystallinity, conductivity, and surface area. Among the composites, MOF(Ni)‐GR(4%) exhibited the best performance in alkaline media, requiring 268 mV overpotential at −10 mA cm^−2^ with a Tafel slope of 108 mV dec^−1^, and showing superior durability compared with Pt/C. Electrochemical active surface area (ECSA) and electrochemical impedance spectroscopy (EIS) analyses further revealed that graphene effectively reduced charge‐transfer resistance and increased the number of accessible active sites, thereby accelerating electron transport and improving HER kinetics. These findings highlight the vital role of graphene in enhancing the conductivity and catalytic efficiency of MOF‐based HER electrocatalysts. However, the intrinsically poor electrical conductivity of pristine Ni‐MOFs remains the key bottleneck, leading to large charge‐transfer resistance and sluggish HER kinetics when the framework is used alone. In this work, graphene mainly serves as a conductive additive to alleviate this limitation, but the catalyst still relies on external carbon networks rather than an intrinsically conductive MOF architecture [95].

Pushing beyond powder‐based composites, Yan et al. developed a freestanding 3D heterostructure film (Ni@N‐HCGHF) through a MOF‐assisted strategy, where Ni‐MOF‐derived carbon nanotubes (CNTs) interconnected rGO sheets to form a conductive and porous framework (Figure 8d). The synergistic combination of Ni nanoparticles encapsulated in N‐doped carbon shells with the CNT@rGO network provided abundant defect sites, hierarchical porosity, and efficient pathways for electron/mass transport. As a result, the optimized film required only 95 mV overpotential to deliver 10 mA cm^−2^ for HER, while maintaining excellent long‐term stability. This work highlights the promise of MOF‐derived freestanding 3D films as scalable and multifunctional electrocatalysts for sustainable energy conversion (Figure 8e,f). Overall, the observed performance enhancement is likely dominated by structural effects, such as increased specific surface area and improved mass/charge transport enabled by the 3D porous architecture, rather than solely by changes in intrinsic active‐site activity [96].

Gothandapani et al. developed Ni‐Ti_3_C_2_ MXene composites by calcining Ni‐MOF precursors, yielding highly porous structures with enlarged surface area and abundant exposed sites. Structural analyses confirmed the successful transformation, while electrochemical tests in alkaline media showed excellent HER performance with an overpotential of 181 mV at 10 mA cm^−2^, a Tafel slope of 56 mV dec^−1^, and low charge‐transfer resistance (1.98 Ω). The improved activity was attributed to the synergistic interaction between Ni species and the conductive Ti_3_C_2_ framework, which enhanced charge transport and site accessibility. The composites also exhibited better kinetics and stability in alkaline than in acidic conditions, underscoring their potential as MOF‐derived electrocatalysts for sustainable hydrogen production. However, the nature of the real active phase remains unclear, as the calcination of Ni‐MOF in the presence of Ti_3_C_2_T_x_ likely generates multiple Ni species, whose individual contributions to HER activity are not clearly distinguished [97].

Taking advantage of structural reconstruction, Wei et al. reported a novel 2D belt‐like superstructure of graphene nanocages (SGNCs) fabricated by direct thermal exfoliation of Ni‐MOF nanorods (Figure 8g). The resulting material consisted of interconnected N‐doped graphitic carbon layers embedding both Ni nanoparticles and single atoms. The optimized SGNC‐900 catalyst exhibited remarkable HER performance in alkaline media, achieving an ultralow overpotential of 32 mV at 10 mA cm^−2^, a small Tafel slope of 39 mV dec^−1^, and an exchange current density of 1.58 mA cm^−2^, comparable to commercial Pt/C. Mechanistic investigations demonstrated that the synergy between Ni single atoms and Ni@C nanoparticles reduced the energy barrier for water dissociation and enhanced H* adsorption, accounting for the superior activity. Additionally, SGNC‐900 provided a large ECSA (468 cm^2^), nearly 100% Faradaic efficiency, and exceptional durability over 2000 cycles without structural degradation (Figure 8h,i). These findings highlight MOF‐derived SGNCs as highly active and robust HER electrocatalysts operable across acidic, neutral, and alkaline environments. Nevertheless, the evidence for the presence and stability of Ni single atoms remains insufficient, as direct atomic‐level characterization is limited [98].

#### 4.1.2 Oxygen Evolution Reaction (OER)

OER is a critical anodic process in various renewable energy conversion and storage systems, including water electrolysis for hydrogen production, metal‐air batteries, and artificial photosynthesis [99, 100, 101]. However, OER involves a sluggish four‐electron transfer pathway with multiple proton‐coupled steps, resulting in high overpotentials and slow kinetics, which significantly limit the overall energy efficiency of these technologies. While noble metal oxides (RuO_2_ and IrO_2_) remain the benchmark OER catalysts due to their excellent activity, their scarcity, high cost, and insufficient durability severely restrict their large‐scale application. Therefore, the development of earth‐abundant, low‐cost, and highly efficient OER electrocatalysts is indispensable to enable the practical deployment of sustainable energy systems. In MOF‐2D hybrid systems, the porous MOF framework supplies abundant redox‐active metal centers, while the 2D component establishes continuous electron‐conduction pathways and mechanical robustness. This synergistic integration promotes efficient electron/ion transport and stabilizes high‐valence intermediates, effectively reducing kinetic barriers and improving durability under oxidative conditions, rendering MOF‐2D hybrids promising candidates for OER catalysis.

**FIGURE 9:**
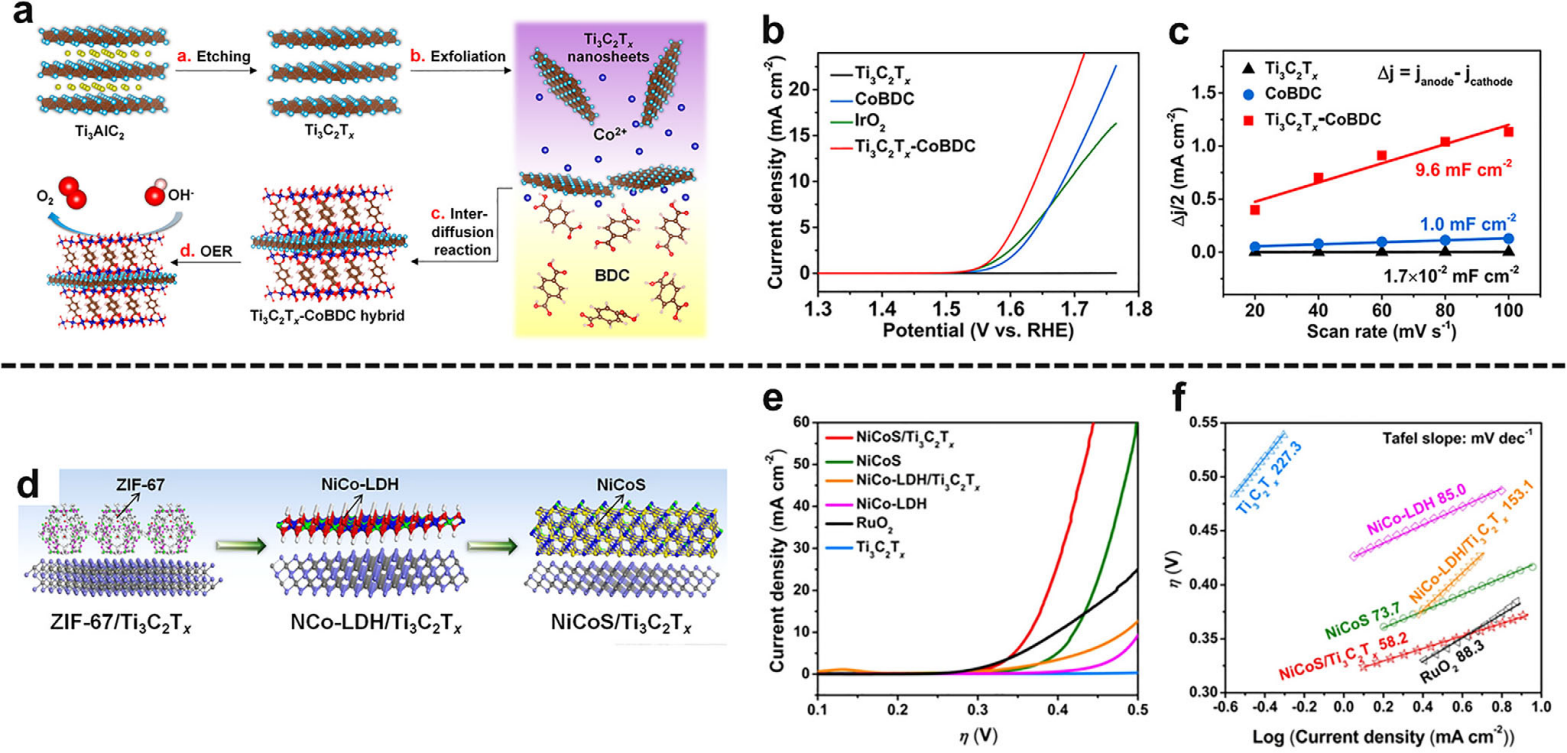
(a) Schematic illustration of the synthesis of Ti_3_C_2_T_x_‐CoBDC. (b) LSV curves of Ti_3_C_2_T_x_, CoBDC, IrO_2_, and Ti_3_C_2_T_x_‐CoBDC. (c) Double‐layer capacitance plots of Ti_3_C_2_T_x_, CoBDC, and Ti_3_C_2_T_x_‐CoBDC. Reproduced with permission [69]. Copyright 2017, American Chemical Society. (d) Schematic illustration of the synthesis of NiCoS/Ti_3_C_2_T_x_. (e) LSV curves of NiCoS/Ti_3_C_2_T_x_, NiCoS, NiCo‐LDH/Ti_3_C_2_T_x_, NiCo‐LDH, RuO_2_, and Ti_3_C_2_T_x._ (f) Tafel plots calculated from LSV curves in (e). (d–f) Reproduced with permission [102]. Copyright 2018, American Chemical Society.

To demonstrate the potential of interfacial engineering, Zhao et al. synthesized a MXene‐MOF hybrid catalyst (Ti_3_C_2_T_x_‐CoBDC) via an interdiffusion reaction, achieving seamless coating of 2D CoBDC layers on Ti_3_C_2_T_x_ nanosheets (Figure 9a). The resulting hybrid displayed excellent OER activity, requiring only 1.64 V vs. RHE to deliver 10 mA cm^−2^ in 0.1 m KOH with a low Tafel slope of 48.2 mV dec^−1^, outperforming commercial IrO_2_ and comparable to state‐of‐the‐art transition metal catalysts (Figure 9b,c). The superior performance was attributed to the synergistic interface between porous CoBDC nanosheets and conductive, hydrophilic Ti_3_C_2_T_x_, which enhanced charge transfer, accelerated ion transport, and facilitated electrolyte penetration. Moreover, when applied as an air cathode in a rechargeable Zn‐air battery, Ti_3_C_2_T_x_‐CoBDC enabled stable cycling sufficient to power an LED, highlighting its practical potential. This work demonstrates the versatility of MXene/MOF interfacial hybridization for the development of next‐generation OER electrocatalysts. The stability evaluation is limited to 10 000 s at 1.64 V vs RHE, with only 84% current retention, which is inadequate to assess prolonged operation or industrially relevant conditions [69].

Building on this concept of interface control, Tan et al. constructed 2D Co_2_Ni‐MOF/Ti_3_C_2_T_x_ composites through an electrostatically directed assembly, where positively charged Co_2_Ni‐MOF nanosheets were tightly anchored onto negatively charged Ti_3_C_2_T_x_ layers. This 2D/2D heterostructure maximized the exposure of active sites and enhanced charge‐transfer kinetics due to the high conductivity of MXene. Among the catalysts, Co_2_Ni‐MOF@MX‐1 exhibited the best OER activity, with an overpotential of 265 mV at 10 mA cm^−2^ and a Tafel slope of 51.7 mV dec^−1^. Compared to pristine MOFs, the hybrid facilitated faster electron/ion transport, suppressed nanosheet stacking, and converted the OER from a charge‐transfer‐limited to a reaction‐limited process. This work highlights electrostatically guided assembly as a versatile strategy to design ultrathin MOF/MXene heterostructures with superior OER activity and durability, offering insights for scalable water‐splitting technologies. Although the enhanced OER activity is attributed to the designed Co_2_Ni‐MOF/MXene interface, the catalyst undergoes pronounced hydroxide reconstruction under OER conditions, with in situ formation of CoOOH/NiOOH phases evidenced by post‐OER Raman and XPS. As a result, the pristine MOF structure is no longer the true active phase during operation, making it difficult to decouple the intrinsic contribution of the MOF/MXene heterostructure from that of the reconstructed metal (oxy)hydroxides, and introducing uncertainty in structure–activity correlations [47].

Furthermore, extending the strategy toward MOF‐derived transformations, Zou and coworkers reported a hierarchical porous Ni‐Co sulfide (NiCoS) coupled with Ti_3_C_2_T_x_ MXene via a MOF‐derived strategy, where ZIF‐67 precursors were in situ converted on exfoliated MXene sheets (Figure 9d). The resulting NiCoS/Ti_3_C_2_T_x_ hybrid exhibited strong interfacial coupling, high conductivity, and a large electrochemically active surface area, collectively enabling superior OER activity. Electrochemical evaluation in alkaline electrolyte showed that the optimized catalyst required only 365 mV overpotential at 10 mA cm^−2^, with a Tafel slope of 58.2 mV dec^−1^, both outperforming commercial RuO_2_ (Figure 9e,f). Turnover frequency calculations further confirmed its high intrinsic activity. Post‐catalytic analysis revealed an in situ transformation of NiCoS into a NiCoOOH‐NiCoS assembly, with NiCoOOH identified as the intrinsic active species responsible for water oxidation. Moreover, the hybrid maintained stable operation over long‐term testing and demonstrated excellent charge–discharge performance when applied as a Zn‐air battery cathode. This study highlights the synergistic role of MOF‐derived bimetallic sulfides and conductive MXenes in achieving high‐performance, noble‐metal‐free OER electrocatalysts, while also providing mechanistic insight into surface phase evolution under operating conditions. However, the irreversible structure transformation also introduces limitations, as the active phase differs from the as‐synthesized material, complicating mechanistic attribution [102].

From the perspective of LDHs, Hu et al. developed a hierarchically structured CoFe layered double hydroxide (CoFe MLDH) anchored on Ti_3_C_2_ MXene via an etching‐coprecipitation method using ZIF‐67 as precursor. The conductive and hydrophilic Ti_3_C_2_ support provided abundant accessible active sites and enabled strong interfacial electronic coupling, effectively tuning the adsorption strength of OER intermediates and enhancing intrinsic catalytic activity. Consequently, CoFe MLDH/Ti_3_C_2_ exhibited excellent OER performance with ultralow overpotentials of 170 and 238 mV at 10 and 100 mA cm^−2^, respectively, together with robust durability. Both experimental evidence and DFT calculations confirmed that the synergistic interaction between MOF‐derived LDHs and MXene facilitated rapid charge transfer and optimized intermediate binding. This work demonstrates the rational integration of MOF‐derived LDHs with MXene as a powerful strategy to construct high‐performance OER electrocatalysts. Nevertheless, the reported metrics are strongly substrate‐dependent, as tests conducted on nickel foam significantly benefit from its intrinsic conductivity, surface roughness, and redox activity, making it difficult to disentangle the catalyst's intrinsic performance from contributions of the Ni‐based support [103].

Beyond rigid frameworks, multifunctional hydrogel systems have also been explored. For instance, Raza et al. reported a multifunctional TiNbC/MOF@SA‐H hydrogel electrocatalyst, constructed by integrating TiNbCT_x_ MXene, Ni/Co‐based MOFs, and sodium alginate into a 3D hybrid network. The hydrogel architecture provided abundant exposed active sites, a large surface area, and enhanced charge‐transfer capability. Electrochemical testing in alkaline media revealed excellent OER performance, with low overpotentials of 185, 188, and 204 mV at 10, 20, and 50 mA cm^−2^, respectively, and a Tafel slope of 84 mV dec^−1^, surpassing TiNbC/MOF and approaching IrO_2_ benchmarks. The composite also exhibited a high double‐layer capacitance (9.43 mF cm^−2^) and superior durability, retaining activity over 1000 cycles and 24 h continuous operation. These results indicate that the synergistic combination of MXene conductivity, MOF porosity, and hydrogel stability effectively accelerates OER kinetics and ensures long‐term robustness, highlighting TiNbC/MOF@SA‐H as a scalable strategy for efficient water oxidation. However, mechanistic evidence remains limited, as the study provides minimal identification of the true active phase, lacks operando analysis of hydrogel–MXene–MOF interactions, and does not clarify how the hydrogel environment modulates adsorption energetics or reaction pathways [104].

Integrating GO into MOF‐based hybrids for OER provides a highly conductive, defect‐rich, and chemically interactive support that prevents MOF aggregation, enhances electron transport, and maximizes active site exposure. Taking these advantages, Li et al. developed a bottom‐up, GO‐assisted strategy for the large‐scale synthesis of ultrathin sandwich‐like MOF/GO/MOF (MGM) nanosheets, where Ni/Co‐BDC MOFs were uniformly anchored on both sides of GO. The ordered crystalline architecture enabled full exposure of unsaturated metal sites and significantly shortened charge‐transfer pathways, thereby enhancing reaction kinetics. The optimized MGM_7_ nanosheets exhibited remarkable OER activity, requiring only 224 mV overpotential at 10 mA cm^−2^, which outperformed most reported MOF‐based electrocatalysts. The superior catalytic behavior was attributed to the synergistic contributions of the ultrathin 2D structure, conductive GO substrate, and optimized bimetallic composition. This work underscores the scalability and high efficiency of MGM nanosheets for oxygen evolution catalysis. Nevertheless, the catalyst displays a relatively high Tafel slope (123.4 mV dec^−1^), indicating sluggish reaction kinetics and suggesting that the rate‐determining step remains insufficiently optimized despite the improved geometric and electronic structure [105].

Yan et al. designed a freestanding 3D heterostructure film (Ni@N‐HCGHF) using a MOF‐assisted strategy, in which Ni‐MOF‐derived carbon nanotubes interlinked reduced GO sheets to construct a highly conductive and porous framework. The combination of N‐doped carbon shells and embedded Ni nanoparticles generated abundant defect sites, hierarchical porosity, and efficient electron transport channels. Benefiting from this architecture, the electrode delivered outstanding OER activity, requiring only 260 mV overpotential at 10 mA cm^−2^ with a Tafel slope of 63 mV dec^−1^, surpassing benchmark RuO_2_ and ranking among the best non‐noble‐metal catalysts. Moreover, the robust, freestanding configuration ensured excellent durability and scalability, highlighting the potential of MOF‐derived 3D films for efficient water oxidation and overall water splitting. However, the performance improvement appears largely structure‐driven, arising from enhanced porosity, CNT‐rGO connectivity, and increased site accessibility, making it difficult to evaluate the intrinsic activity of the Ni‐based active centers independent of the engineered macro‐/mesostructure [96].

Finally, highlighting the role of heteroatom‐doped carbons, Ferraz et al. reported a bifunctional Co‐N‐doped carbon catalyst synthesized by introducing Co ions during dopamine polymerization, enabling direct doping into the carbon framework and localized growth of ZIF‐67 polyhedra. After pyrolysis, the material contained well‐dispersed Co nanoparticles encapsulated in graphitic carbon shells on thin nanosheets, stabilized by a C_3_N_4_ template. This architecture promoted a high density of active Co‐N sites, improved conductivity, and prevented particle aggregation. As a result, the catalyst exhibited excellent OER activity with substantially lower overpotentials (∼510 mV) compared to Ir/C. This work highlights cobalt insertion during dopamine polymerization as an effective strategy to construct stable and highly active OER electrocatalysts. Nevertheless, the required overpotential remains relatively high, indicating that the intrinsic Co─N─C active centers are still not sufficiently optimized for low‐potential OER despite the improved structural engineering [106].

#### 4.1.3 Oxygen Reduction Reaction (ORR)

ORR is a critical process in fuel cells and metal‐air batteries, directly determining the energy conversion efficiency [107, 108, 109]. However, the intrinsic sluggish kinetics of the multi‐electron transfer process usually demand precious‐metal‐based catalysts such as Pt, whose high cost, scarcity, and poor durability severely limit large‐scale applications. Therefore, developing efficient, low‐cost, and durable non‐precious electrocatalysts is of great importance for practical ORR technologies. MOFs enable precise modulation of metal nodes and heteroatom coordination, thereby tailoring electronic structures that are favorable for ORR pathways. Incorporation of 2D materials further enhances electrical conductivity and structural integrity, ensuring rapid electron delivery and sustained catalytic performance. The resulting MOF–2D hybrids thus provide an attractive platform for developing non‐precious ORR catalysts with improved activity and operational stability.

**FIGURE 10:**
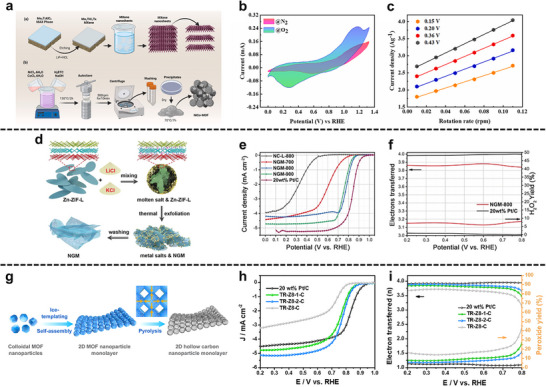
(a) Schematic illustration of the synthesis of MXene and NiCo‐MOF. (b) CV curves of B‐MNC with and without O_2_ saturation. (c) Koutecky‐Levich (K–L) graphs of B‐MNC under different potentials. Reproduced with permission [110]. Copyright 2025, Elsevier. (d) Schematic illustration of the synthesis of NGM. (e) LSV curves of NC‐L‐800, NGM‐700, NGM‐800, NGM‐900, and 20 wt.% Pt/C. (f) Transferred electron number and H_2_O_2_ yield of NGM‐800 and 20 wt.% Pt/C. Reproduced with permission [71]. Copyright 2019, Wiley‐VCH. (g) Schematic illustration of the synthesis of the 2D hollow carbon nanoparticle monolayer. (h) LSV curves of TR‐Z8‐C, TR‐Z8‐1‐C, TR‐Z8‐2‐C, and 20 wt.% Pt/C. (i) Transferred electron number and H_2_O_2_ yield of TR‐Z8‐C, TR‐Z8‐1‐C, TR‐Z8‐2‐C, and 20 wt.% Pt/C. Reproduced with permission [114]. Copyright 2022, American Chemical Society.

Jalalah et al. developed a bimetallic MXene@NiCo‐MOF composite (B‐MNC) via a green hydrothermal method and investigated its performance as an oxygen reduction electrocatalyst (Figure 10a). In an O_2_‐saturated alkaline electrolyte, B‐MNC displayed a cathodic reduction peak with an onset potential of 0.7 V vs. RHE and a cathodic peak at 0.27 V. Rotating disk electrode (RDE) measurements and Koutecky‐Levich (K‐L) analysis confirmed first‐order kinetics with an effective electron transfer number of 4.5, indicative of a favorable four‐electron reduction pathway (Figure 10b,c). Furthermore, chronoamperometry demonstrated that the composite retained 80% of its initial ORR activity after 8000 s of continuous operation, highlighting its durability. The improved activity and stability were attributed to the synergistic effect between the high conductivity of MXene and the abundant active sites of NiCo‐MOF, establishing B‐MNC as a promising non‐noble‐metal catalyst for oxygen reduction. However, the relatively low onset potential reflects limited intrinsic ORR activity, indicating room for improvement in active‐site optimization and electronic structure tuning. [110]

Li et al. reported a spatial‐isolation strategy to synthesize atomically dispersed cobalt sites anchored on hierarchical porous N‐doped carbon/rGO (Co‐SAs/N‐C/rGO). By tuning Zn/Co ratios in ZnCo‐ZIF precursors, isolated CoN_4_ moieties were stabilized within a conductive carbon matrix. The optimized catalyst demonstrated pH‐universal ORR activity with half‐wave potentials of 0.84, 0.77, and 0.65 V vs RHE in alkaline, acidic, and neutral electrolytes, respectively, comparable to commercial Pt/C. It also exhibited an onset potential up to 1.01 V (in alkaline medium), high kinetic current densities, outstanding methanol tolerance, and long‐term durability. When applied in Zn‐air batteries, Co‐SAs/N‐C/rGO achieved an open‐circuit voltage of 1.52 V and a discharge capacity of 671.94 mAh g^−1^, outperforming Pt/C + RuO_2_. The excellent performance was ascribed to the intrinsic activity of atomic CoN_4_ sites, along with the high surface area and hierarchical porosity that facilitated efficient electron and mass transport. Nevertheless, the intrinsically low Co loading typical of single‐atom catalysts limits the total density of accessible active sites, constraining achievable current outputs at high rates and posing challenges for practical large‐scale applications [111].

To further advance non‐precious carbon catalysts, Xia et al. developed a nitrogen‐doped graphene nanomesh (NGM) through a two‐step dimensional‐reduction strategy, in which Zn‐ZIF nanoleaves were thermally exfoliated using mixed metal chlorides (Figure 10d). The resulting ultrathin NGM (∼1.3 nm) exhibited a high surface area (1329.5 m^2^ g^−1^), significant nitrogen doping (4.68 at%), and abundant hierarchical porosity with defect‐rich graphene edges. These structural merits provided an exceptionally high density of accessible active sites and efficient charge/mass transport, leading to outstanding ORR activity (half‐wave potential of 0.781 V and onset potential of 0.86 V), especially in acidic electrolytes, where it outperformed most metal‐free carbon catalysts (Figure 10e,f). The superior performance was attributed to the synergistic contribution of nitrogen dopants, exposed defective edges, and hierarchical porosity. This study highlights the dimensional reduction of MOFs as an effective pathway for scalable synthesis of advanced low‐dimensional carbon catalysts for ORR across different pH conditions. Nonetheless, despite its strong performance among metal‐free systems, the NGM catalyst still shows inferior activity to Pt/C benchmarks, especially in terms of onset/half‐wave potentials and kinetic current, highlighting the remaining gap between advanced carbon architectures and state‐of‐the‐art noble‐metal catalysts [71].

Meanwhile, Fenoy et al. explored polymer‐MOF interfaces by assembling multilayers of PEI/PEDOT: PSS and incorporating ZIF‐8 nanocrystals to boost ORR activity under neutral conditions. Optimized multilayer assemblies of PEI/PEDOT: PSS (four bilayers) ensured efficient conductivity and catalytic balance, while the subsequent incorporation of ZIF‐8 nanocrystallites introduced hierarchical porosity and acted as an O_2_ reservoir. This combination enabled selective O_2_ preconcentration without impeding ion transport, thereby significantly improving ORR activity compared to pristine polymer films. The work demonstrates a modular and scalable strategy for fabricating functional polymer‐MOF interfaces with enhanced electrocatalytic performance in neutral electrolytes. However, the study reports limited electrochemical metrics, providing only partial performance benchmarks and lacking key kinetic descriptors such as Tafel slope, electron‐transfer number, or long‐term stability, which restricts a full evaluation of catalytic competitiveness [112].

Extending to hybrid polymer‐MOF systems, Guo et al. designed a conjugated microporous polymer‐on‐MOF (CMP‐on‐MOF) hybrid precursor to construct N‐decorated sheet‐on‐rod heterostructures with hierarchical porosity and uniformly dispersed Co_5.47_N, Co_0.7_Fe_0.3_, and Fe/Co‐N_x_ species after pyrolysis [113]. The optimized catalyst (PM‐900) provided abundant active centers and a conductive nanosheet‐nanorod framework, which synergistically boosted ORR activity in alkaline media, achieving a half‐wave potential of 0.86 V and an onset potential of 1.03 V. Benefiting from this architecture, PM‐900 exhibited a narrow potential gap (Δ*E* ≈ 0.702 V) between ORR and OER, outperforming materials derived from either CMP or MOF alone. When applied in Zn‐air batteries, the catalyst achieved a high specific capacity of 782 mAh g^−1^, a power density of 184.5 mW cm^−2^, and excellent durability over 1000 cycles, surpassing Pt/C + IrO_2_ benchmarks. This work underscores the effectiveness of combining CMPs with MOFs to simultaneously tune structural geometry and composition for developing advanced non‐precious ORR electrocatalysts. Nevertheless, the BET surface area remains moderate, limiting maximal active‐site exposure and constraining further improvements in high‐rate ORR performance.

Finally, Song et al. employed an ice‐templating strategy to synthesize 2D layered MOF nanoparticle superstructures, which could be tuned into mono‐ and bilayer assemblies (Figure 10g) [114]. Upon pyrolysis, these assemblies were transformed into carbon nanoparticle superstructures that preserved the quasi‐ordered arrangement and developed hollow interiors due to the outward contraction of MOF precursors. The resulting 2D carbon frameworks combined hierarchical porosity, abundant exposed active sites, and interconnected conductive pathways, which synergistically enhanced ORR performance, as evidenced by a half‐wave potential of 0.77 V and an onset potential of 0.90 V. In alkaline media, the catalyst delivered up to a tenfold increase in current density at 0.8 V compared with isolated carbon nanoparticles (Figure 10h,i). This work demonstrates the effectiveness of ice‐templating as a scalable strategy to design MOF‐derived 2D porous carbons with superior ORR catalytic activity. However, the material still exhibits relatively high resistance, indicating suboptimal charge‐transport efficiency and suggesting that electrical conductivity remains a limiting factor for further performance enhancement.

#### 4.1.4 Carbon Dioxide Reduction Reaction (CO_2_RR)

The electrochemical reduction of CO_2_ (CO_2_RR) offers a sustainable pathway to mitigate carbon emissions while generating value‐added chemicals and fuels such as CO, formate, methanol, and hydrocarbons. However, CO_2_RR suffers from sluggish kinetics, multiple competing pathways, and high energy barriers, making the development of efficient and selective electrocatalysts a critical challenge. MOFs, owing to their tunable porosity, high surface area, and versatile coordination chemistry, have emerged as promising candidates. In particular, MOF‐2D hybrid materials further enhance performance by exposing abundant accessible active sites, shortening charge/mass transport paths, and allowing precise electronic modulation of catalytic centers. These features collectively enable improved activity, selectivity, and stability, highlighting MOF‐2D hybrid materials as a compelling platform for next‐generation CO_2_ reduction electrocatalysts.

**FIGURE 11:**
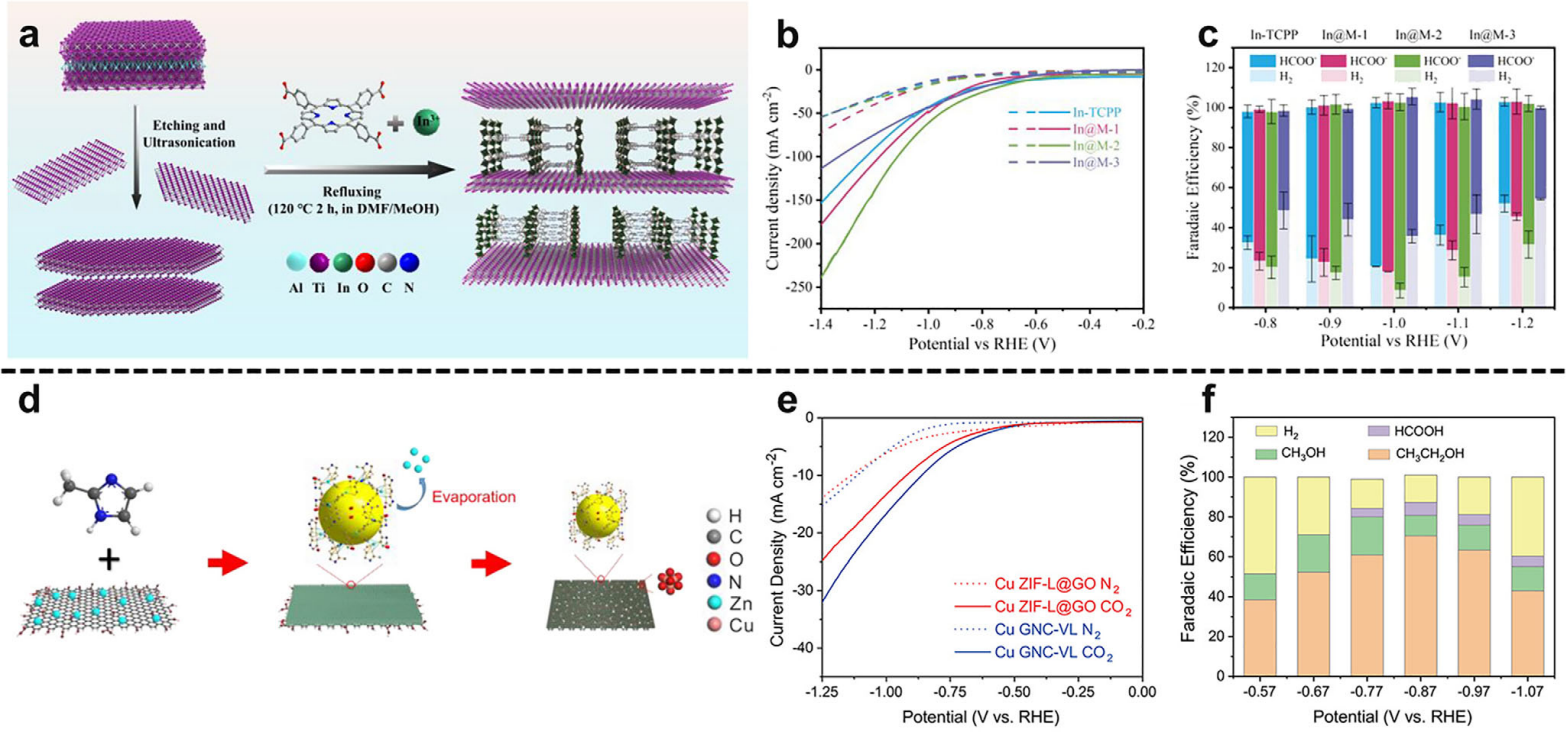
(a) Schematic illustration of the synthesis of In‐TCPP@MXene. (b) LSV curves of In‐TCPP, In@M‐1, In@M‐2, and In@M‐3 under CO_2_ saturation (solid) and Ar saturation (dashed). (c) Faradaic efficiency of In‐TCPP, In@M‐1, In@M‐2, and In@M‐3 under different potentials. Reproduced with permission [115]. Copyright 2025, American Chemical Society. (d) Schematic illustration of the synthesis of Cu GNC‐VL. (e) LSV curves of Cu GNC‐VL and Cu ZIF‐L@GO in 0.5 m KHCO_3_ electrolyte with CO_2_ saturation (solid) and N_2_ saturation (dashed). (f) Faradaic efficiency of Cu GNC‐VL under different potentials. Reproduced with permission [116]. Copyright 2019, American Chemical Society.

Zhu et al. integrated an In‐based porphyrinic MOF (In‐TCPP) with conductive Ti_3_C_2_T_x_ MXene nanosheets to address the poor conductivity of pristine MOFs in CO_2_ electroreduction (Figure 11a). The resulting hybrid catalyst (In‐TCPP@MXene) achieved a remarkable Faradaic efficiency of 94.0% toward formate and a 2.2‐fold increase in current density (102.5 mA cm^−2^) compared to In‐TCPP alone (Figure 11b,c). Importantly, it enabled continuous production of concentrated formic acid (∼0.22 M) in a solid‐state MEA device, demonstrating strong practical potential. Mechanistic studies combining in situ ATR‐FTIR and DFT confirmed that MXene not only served as a conductive scaffold but also tuned the electronic structure of In sites to optimize *OCHO adsorption and accelerate charge transfer. This work highlights the critical role of MOF‐MXene interfacial synergy in achieving efficient and selective CO_2_ reduction, offering guidance for designing MOF‐based hybrids for scalable formate production. However, possible structure transformation of MOF components under electrochemical conditions, such as ligand degradation, partial demetalation, or restructuring of In coordination sites, remains insufficiently examined, leaving uncertainties regarding the true active phase and long‐term stability [115].

Moving from formate production to multi‐carbon fuels, Zhang et al. designed a Cu/Cu_2_O nanocomposite (Cu GNC‐VL) supported on MOF‐derived carbon by vertically growing ZIF‐L on GO, followed by high‐temperature carbonization [116]. The resulting cross‐like, highly ordered architecture ensured abundant exposed active sites and efficient charge transport (Figure 11d). Owing to the synergistic contribution of Cu (111) facets for asymmetric CO_2_ adsorption and Cu_2_O (111) facets for favorable C─C coupling, the catalyst exhibited excellent CO_2_RR performance, achieving a Faradaic efficiency of 70.52% toward ethanol at a current density of 10.4 mA cm^−2^ (−0.87 V *vs*. RHE) (Figure 11e,f). This study demonstrates how geometry‐modulated MOF‐derived architectures can effectively steer product selectivity toward multi‐carbon (C_2_) fuels. However, the achievable current density remains relatively low, limiting overall productivity and indicating that mass‐transport efficiency and active‐site density require further optimization for practical CO_2_‐to‐ethanol conversion.

To tackle conductivity and site accessibility issues in bulk MOFs, Jia et al. developed a Cu‐THQ MOF anchored on edge‐functionalized graphene (Cu‐THQ‐EFG) to overcome the intrinsic drawbacks of bulk MOFs, including poor conductivity and inaccessible active sites [117]. The EFG substrate not only offered high conductivity and aqueous stability but also abundant edge functionalities that effectively suppressed MOF aggregation and enhanced charge transfer. Benefiting from these structural advantages, Cu‐THQ‐EFG exhibited outstanding CO_2_RR performance with an onset potential of −0.22 V *vs*. RHE, close to the thermodynamic limit, and delivered a maximum Faradaic efficiency of 31.7% toward formate (yield: 16.8 µmol h^−1^ cm^−2^) at −0.25 V *vs*. RHE with a current density of 3 mA cm^−2^. Notably, formate was the exclusive liquid product, simplifying product separation. The catalyst also demonstrated excellent stability over 15 h of continuous operation, outperforming Cu‐THQ‐GO and other reported Cu‐MOF‐based systems. This study underscores the effectiveness of EFG as a conductive substrate to boost the activity and accessibility of MOF‐derived CO_2_RR catalysts. However, the Faradaic efficiency remains relatively low, indicating limited selectivity toward formate and suggesting that further tuning of electronic structure and active‐site geometry is necessary to achieve competitive product yields.

Synthesizing the progress in electrocatalysis, while MOF‐2D hybrids have achieved remarkable intrinsic activities in half‐cell configurations, the next frontier lies in microenvironment engineering and device integration. The current focus on active site turnover must expand to address mass transport limitations at industrial current densities (>1 A cm^−^^2^). Therefore, future research should prioritize the integration of these hybrids into membrane electrode assemblies (MEAs), where the optimization of local pH, gas diffusion layers, and triple‐phase boundaries becomes as critical as the catalyst itself for realizing practical energy conversion technologies.

### 4.2 Energy Storage

The relentless pursuit of advanced energy storage solutions has propelled the exploration of novel material architectures. Hybrids integrating MOFs and 2D materials represent a paradigm shift, leveraging the unique strengths of each component to overcome fundamental limitations in batteries and supercapacitors. MOFs offer intriguing structural and chemical tunability, exceptionally high surface area, well‐defined porosity for ion transport, and abundant active sites [118, 119, 120, 121]. However, they often suffer from inherent drawbacks like limited electrical conductivity, insufficient mechanical stability during cycling, and susceptibility to degradation in harsh electrochemical environments. Conversely, 2D materials provide exceptional electrical and thermal conductivity, robust mechanical strength, fast in‐plane charge transport, and versatile surface chemistry; however, they suffer from restacking issues [17, 122, 123, 124]. Dimensional coupling engineering aims to synergistically combine these attributes, creating hybrids where the MOF acts as a functional porous scaffold or active material reservoir, while the 2D material provides structural integrity, conductive pathways, and interfacial modulation [125, 126].

Before exploring specific applications, it is crucial to understand the core synergistic mechanisms enabling superior performance of MOF‐2D hybrids in energy storage applications: (1) Enhanced electrical conductivity: the intrinsically low conductivity of most MOFs is a major bottleneck for charge transfer in electrodes. 2D materials, particularly graphene and MXenes, act as highly efficient conductive highways, facilitating rapid electron transport to and from the MOF active sites. (2) Improved diffusion kinetics: the hierarchical porosity arising from the combination of MOF micropores/mesopores and the interlayer spacing/defects in 2D materials creates multidimensional pathways for ion diffusion (Li^+^, Na^+^, H^+^, etc.). (3) Structural reinforcement; the robust mechanical properties of 2D materials (graphene, MXenes, h‐BN) provide structural support to the MOF, enhancing mechanical integrity and mitigating pulverization during lithiation/delithiation. (4) Increased accessible surface area and active site exposure; while MOFs have high theoretical surface areas, accessibility can be limited. Integration with 2D materials can prevent restacking of nanosheets and aggregation of MOF particles, maximizing the exposure of active sites within the MOF pores and on the 2D surfaces to the electrolyte. (5) Tailored Interfaces & Porosity; an effective interaction at the MOF/2D interface creates unique synergistic effects. MOF pores offer confined spaces for Li‐ion storage and transport, while the 2D surface facilitates electron transfer. Furthermore, the composite often exhibits hierarchical porosity (micropores from MOF and meso/macropores from the 2D scaffold), optimizing ion diffusion. (6) Synergistic Electrochemical Activity; both components can contribute in synergy to charge storage. MOFs offer pseudocapacitance or conversion/alloying reactions, while 2D materials provide electric double‐layer capacitance (EDLC) or intercalation capacity.

#### 4.2.1 Conventional Li‐Ion Batteries (LIBs)

Conventional Li‐ion batteries (LIBs) often face limitations such as low rate performance, sluggish lithium‐ion diffusion, and limited cycle life, primarily due to poor structural stability and low electrical conductivity of traditional intercalation‐type electrodes [127, 128, 129]. The strategic hybridization of MOFs with 2D materials emerges as a powerful paradigm to synergistically overcome these individual drawbacks, creating a hybrid with superior electrochemical performance for LIB electrodes (anodes, cathodes, separators, and interlayers). Pristine MOFs themselves exhibit Li^+^ storage capability through redox‐active metal centers or organic linkers. However, coupling them with 2D materials enhances their conductivity. One notable example is an aluminum‐based MOF (MIL‐53), uniformly wrapped within graphene sheets synthesized via an ex situ method [130]. Upon battery cycling, the Al‐MOF underwent a lithiation‐induced structural “disordering” that opened up its channels. The graphene nanosheets provided a conductive network and mechanical confinement. As a result, the Al‐MOF/graphene composite's reversible capacity grew from only 60 mAh g^−1^ for pristine Al‐MOF to 400 mAh g^−1^ at 0.1 A g^−1^. This improvement was ascribed to the synergistic effect. Lithium‐ion insertion expands the MOF pores, enabling them to store more Li^+^ ions, while graphene facilitates electron transport and preserves the structure's stability. However, the ex situ approach suffers from weaker interfacial bonding between the MOF and graphene, leading to irreversible side reactions and a relatively low initial coulombic efficiency, as observed in this system.

Furthermore, the inclusion of GO and rGO significantly improves MOF‐based electrode performance by enhancing conductivity and buffering strain. For example, Zhu et al. synthesized a Ni‐MOF/GO hybrid by first anchoring Ni^2+^ ions to GO sheets and then forming Ni‐MOF crystals in situ on the GO surface [131]. The GO substrate binds Ni^2^^+^ via carboxyl groups and prevents MOF agglomeration during synthesis. The resulting Ni‐MOF/GO anode achieved a reversible capacity of 740.8 mAh g^−1^ at 50 mA g^−1^ with nearly 100% retention over 100 cycles. The study attributed this excellent stability to the GO matrix and its *π*–*π* interactions, which enhance electron mobility and delocalize charge, while GO physically holds the Ni‐MOF framework intact during lithiation. However, a potential limitation of in situ growth is its strong dependence on surface functional groups and synthesis conditions, which may limit scalability and reproducibility if not carefully controlled. Similarly, GO‐based anode coated with MOF‐199 via a layer‐by‐layer method [53], where alternate deposition of cupric acetate and organic linker created a porous “pore‐cage‐pore” structure. The layer‐by‐layer (LbL) approach offers several advantages, including precise thickness control and the formation of uniform, conformal MOF coatings with enhanced interfacial contact between GO and the MOF framework, leading to reduced crystallite size, increased surface area, and improved Li^+^ accessibility. Electrochemical tests showed a high initial discharge capacity of 1420 mAh g^−1^ at 0.1 A g^−1^, retaining 91% capacity and 98.9% efficiency after 100 cycles. The composite also exhibited excellent rate capability and significantly lower charge transfer resistance, highlighting the layer‐by‐layer coating's role in enhancing conductivity, ion transport, and cycling stability for LIB anodes. Nevertheless, it suffers from agglomeration (a key limitation of layer‐by‐layer method), which hinders the movement of electrolyte ions and thereby degrades performance.

Overall, these studies clearly demonstrate that integrating MOFs with graphene, GO, or rGO can dramatically enhance Li^+^ storage by improving electronic conductivity, mitigating structural degradation, and promoting synergistic ion‐electron transport. Nevertheless, challenges such as low initial coulombic efficiency, irreversible side reactions, and agglomeration‐induced ion transport limitations remain critical bottlenecks that hinder practical deployment. Addressing these issues through controlled interfacial engineering, optimized composite architectures, and scalable synthesis strategies will be essential to fully realize the potential of MOF‐2D material hybrids as high‐performance LIB anodes. Hybridizing MOFs with conductive MXenes has emerged as a highly promising route for advanced LIB anodes. A notable example is the 2D/2D Ti_3_C_2_T_x_/NiCo‐MOF heterostructure developed via in situ approach, which demonstrates a significant synergistic effect [39]. In this architecture, the NiCo‐MOF component provides an abundance of exposed redox‐active sites for Li^+^ storage, while the underlying Ti_3_C_2_T_x_ network acts as a highly conductive scaffold, ensuring rapid electron and ion transport throughout the electrode. This unique combination results in outstanding performance, delivering a high reversible capacity of 637 mAh g^−1^ at 0.2 A g^−1^ and superior cycling stability compared to the pristine MOF material. The enhanced stability originates from the robust interface formed via in situ synthesis, ensuring strong interfacial contact and electronic coupling. Gong et al. in situ developed a Co‐MOF on both the surface and between the layers of V_2_CT_x_ MXene, expanding its interlayer spacing [40]. The MOF not only contributed extra specific capacity but also inhibited MXene restacking, thereby exposing more Li^+^ active sites. In addition, strong interfacial bonding to the highly conductive and electrochemically stable MXene improved the structural integrity of the Co‐MOF within the composite. Consequently, Co‐MOF/V_2_CT_x_ anodes achieved a high specific capacity of 484.3 mAh g^−1^ after 120 cycles. Notably, in situ approach may induce MOF structural fragmentation and the formation of gel‐like byproducts during prolonged cycling, which restrict Li^+^ ion accessibility and contribute to gradual capacity decay. A third striking example involves the in situ growth of Sn‐based MOF on Ti_3_C_2_T_x_ MXene nanosheets [132]. This in situ strategy ensures effective interfacial contact and homogeneous MOF anchoring, allowing the hybrid to effectively leverage the high theoretical Li‐storage capacity of the Sn‐MOF and the excellent electrical conductivity of MXene. As a result, the composite achieved 1009 mAh g^−1^ at 0.1 A g^−1^, a capacity comparable to pristine Si or Sn anodes. Importantly, impedance spectroscopy showed that adding MXene greatly reduced the electrode's charge‐transfer resistance and enhanced Li^+^ diffusion. In particular, the MXene accelerated electron flow to/from the Sn‐MOF, preventing polarization and sustaining high capacities. Collectively, these examples highlight that MOF/MXene hybrids can effectively combine abundant redox‐active sites with ultrafast electron transport, leading to high capacity and improved cycling stability compared to pristine MOFs. However, issues such as MOF structural fragmentation, interfacial instability, and ion‐accessibility loss during prolonged cycling still constrain long‐term performance. Hence, future efforts must focus on stabilizing MOF frameworks, suppressing restacking and degradation, and precisely engineering MOF/MXene interfaces to fully exploit their synergistic potential in practical LIB anodes.

**FIGURE 12:**
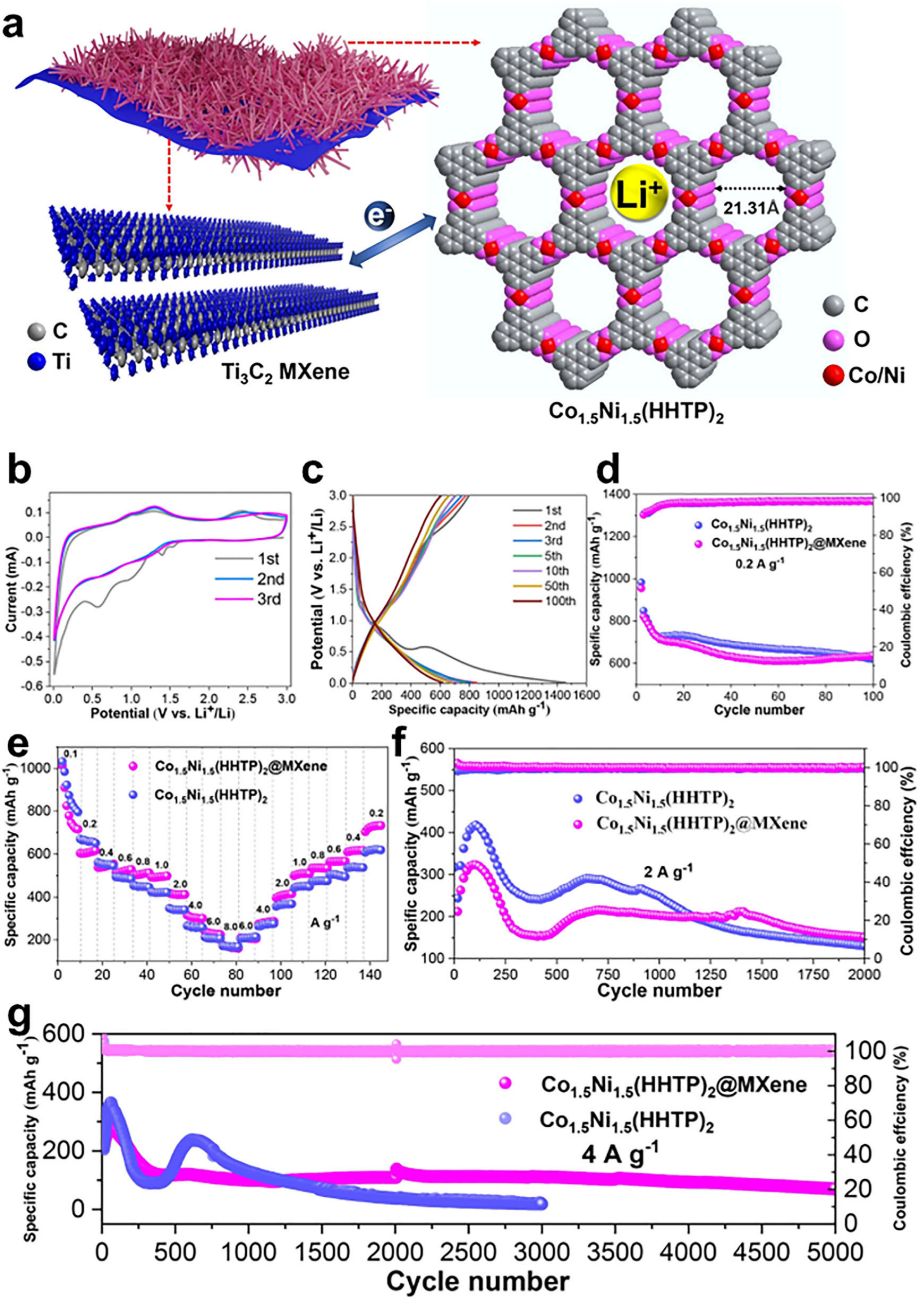
(a) Schematic illustration of bimetallic conductive CoNi‐MOF and MXene hybrid demonstrating Li^+^ ion diffusion, Electrochemical performance of CoNi‐MOF: (b) CV profile at 0.1 mV s^−1^, (c) GCD curves at 0.2 A g^−1^, Electrochemical performance comparison of CoNi‐MOF and CoNi‐MOF@MXene: (d) Cyclic stability at 0.2 A g^−1^, (e) Rate capability demonstration at different current densities, (f,g) Cyclic stability at 2 A g^−1^ and 4 A g^−1^, respectively. Reproduced with permission [133]. Copyright 2025, Elsevier.

Cathodes in LIBs require high‐voltage redox centers, good electronic conductivity, and stability. Conventional cathode materials are layered oxides (LiCoO_2_, lithium nickel manganese cobalt oxides, etc.) or polyanion compounds (LiFePO_4_). MOFs, by themselves, have rarely met these requirements because many MOFs lack sufficient redox potential or conductivity. While less common than anodes, MOF‐2D material hybrids show promise for cathodes, particularly for materials that suffer from low conductivity or structural instability. For example, a conductive 2D MOF (Co_1.5_Ni_1.5_ (HHTP)_2_, where HHTP = hexahydroxytriphenylene) was combined with Ti_3_C_2_ MXene via in situ hydrothermal approach to form a sandwich‐like heterostructure with uniform stacking between the MOF and MXene layers [133]. The CoNi‐HHTP MOF features built‐in redox‐active metal centers and 2D channels that can accommodate Li^+^, while the Ti_3_C_2_ MXene provides excellent conductivity (Figure 12a). This composite (CoNi‐HHTP MOF) delivered a very high reversible capacity of 615 mAh g^−1^ at 0.2 A g^−1^ and an energy density of 826 Wh kg^−1^, far exceeding the typical LIB cathodes. Remarkably, the heterostructure CoNi‐MOF@MXene retained ∼74% of capacity even after 2000 cycles, demonstrating outstanding cyclability (Figure 12b–g). Ex situ spectroscopy indicated that lithiation involves both the quinone oxygens and the metal centers, while the MXene layers greatly facilitate electron transport. The incorporation of MXene enhances the cyclic stability and rate performance; however, it does not improve the specific capacity because excessive MXene coverage due to in situ approach may partially block the Li^+^ active site and impede Li^+^ ion transport. Rational interface and thickness control will therefore be critical to balance conductivity with ion accessibility and to translate such high‐performance laboratory cathodes into practical LIB systems.

#### 4.2.2 Li–S Batteries

**FIGURE 13:**
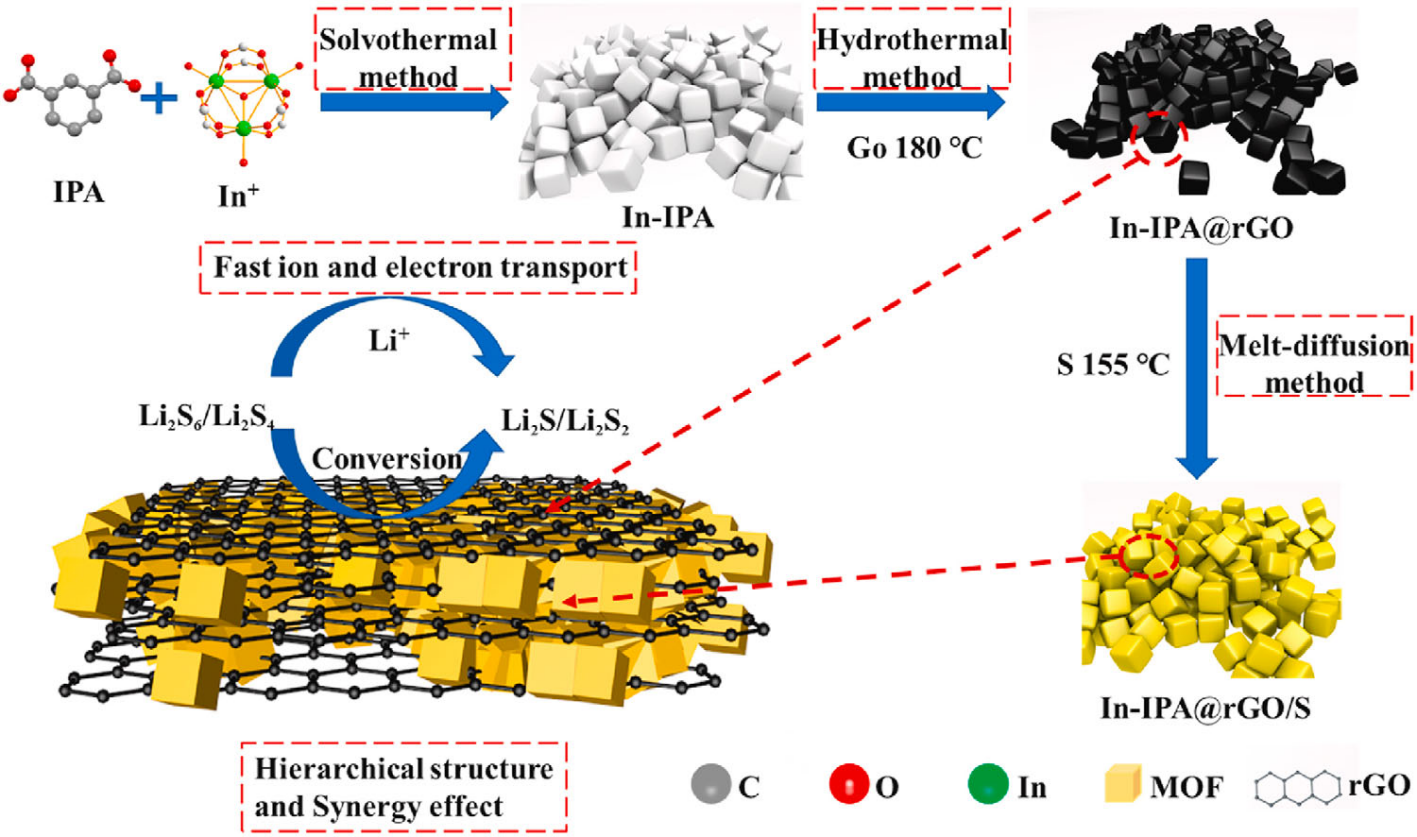
Schematic illustration of the synthesis of the In‐IPA@rGO/S composite. Reproduced with permission [137]. Copyright 2022, Elsevier.

Li–S batteries promise ultra‐high theoretical energy density but are plagued by the “shuttle effect” of soluble lithium polysulfides (LiPS), insulating nature of S/Li_2_S, and large volume changes. MOF‐2D hybrids are extensively researched as sulfur hosts and functional interlayers [134, 135, 136]. In this line, Jiao et al. designed 3D In‐IPA (IPA‐isophthalic acid), coated with rGO via ex situ hydrothermal synthesis, which forms an interconnected network that markedly improves conductivity, accelerates reaction kinetics, and facilitates electron transport (Figure 13) [137]. The ex situ approach allows independent optimization of the MOF framework and rGO conductive phase, enabling flexible structural design and effective composite integration. As a sulfur host, In‐IPA@rGO exhibits exceptional electrochemical performance, delivering a reversible capacity of 898.7 mAh g^−1^ at 0.2C. These findings underscore the synergistic advantages of combining non‐transition MOFs with conductive rGO for Li–S battery applications. In another report, Deng et al. designed 3D hierarchical MOF on rGO as an advanced sulfur host for Li–S batteries. Using a hydrothermal process, MOFs (MIL‐101 and MIL‐53) were grown in close contact with rGO, combining MOFs’ high porosity and polarity with rGO's excellent electrical conductivity and structural flexibility [138]. The rGO layers tightly coat MOF crystals, confining sulfur within pores and suppressing polysulfide dissolution while buffering volume changes during cycling. Enhanced rate performance and reduced charge‐transfer resistance highlight the synergistic effect between MOFs and rGO, demonstrating this architecture as a promising strategy to overcome Li–S battery limitations. However, a key limitation of the ex situ configuration is the weak interfacial integration between rGO and the MOF, which leaves rGO insufficiently decorated with MOF active sites and thus unable to provide effective chemical anchoring for polysulfides. This is evidenced by the control S‐in‐rGO electrode, which exhibited rapid capacity decay after the initial cycles (58% capacity retention with an average decay of 0.11% per cycle). This comparison highlights that strong polysulfide trapping cannot be achieved by rGO alone and underscores the critical role of MOFs, which is inadequately realized in the ex situ approach. These studies suggest that MOF/rGO hybrids effectively mitigate Li–S battery challenges by combining chemical polysulfide anchoring with high porosity and improved conductivity. The rapid fading of rGO‐only hosts highlights that conductivity and physical confinement alone are insufficient without MOF‐assisted chemical trapping. Hence, further optimization is needed to ensure long‐term stability under practical operating conditions.

Interestingly, 3D hierarchical nMOF‐867/Ti_3_C_2_T_x_ heterostructure was synthesized via electrostatic self‐assembly and in situ solvothermal method [139]. Porous nMOF‐867 chemically traps polysulfides through Li─N and Zr─S bonds, while conductive Ti_3_C_2_T_x_ enhances polysulfide redox kinetics and buffers volume expansion. As a sulfur host in Li–S batteries, it delivers high reversible capacities (1302 mAh g^−1^ at 0.2 C) with excellent cycling stability (0.054% decay per cycle over 1000 cycles), demonstrating promising performance. However, the in situ solvothermal process may involve harsh reaction conditions, which could limit scalability and risk partial surface oxidation or degradation of MXene layers.

**FIGURE 14:**
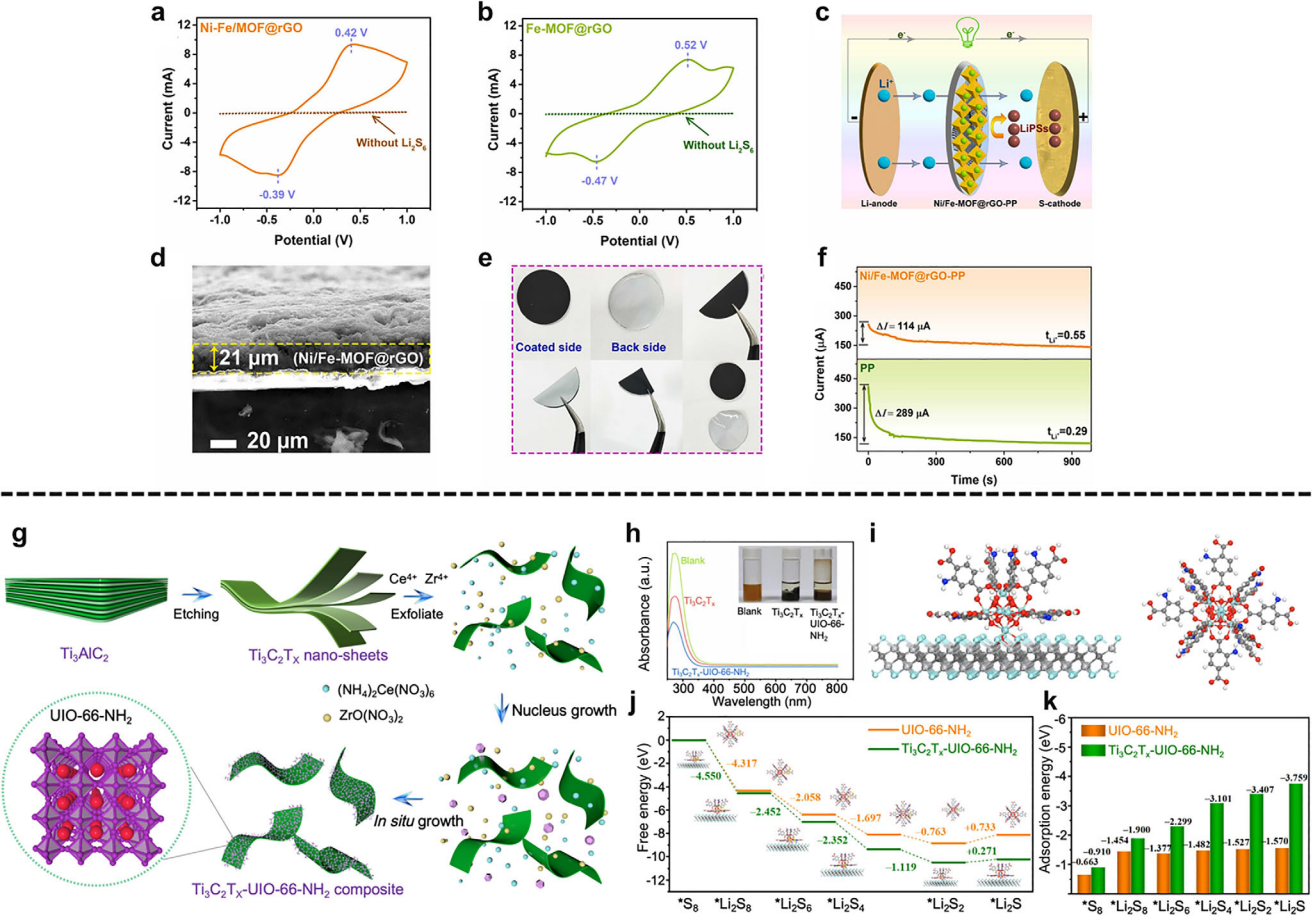
(a,b) CV curves of symmetric cell with Ni,Fe‐MOF @rGO and Fe‐MOF@rGO respectively, (c) Schematic of Li–S cell assembly, (d) Cross sectional scanning electron microscopy (SEM) micrograph of Ni,Fe‐MOF@rGO‐PP separator, (e) Demonstration of the flexibility of Ni,Fe‐MOF @rGO‐PP separator, (f) Transference number analysis (Li ions) of Ni,Fe‐MOF@rGO‐PP separator and PP separator. Reproduced with permission [143]. Copyright 2024, Elsevier. (g) Schematic demonstration of etching, delamination process of MXene and nucleation‐assisted in situ growth of Ti_3_C_2_T_x_‐UiO‐66‐NH_2_ hybrid, (h) UV adsorption in Li_2_S_6_ for Ti_3_C_2_T_x_ and Ti_3_C_2_T_x_‐UiO‐66‐NH_2_, (i) Unit structure diagram of pristine UiO‐66‐NH_2_ and Ti_3_C_2_T_x_‐UiO‐66‐NH_2_ hybrid, and (j,k) DFT calculations illustrating variation of free energy and adsorption energy respectively for UiO‐66‐NH_2_ and Ti_3_C_2_T_x_‐UiO‐66‐NH_2_ hybrid interacting with polysulfides of different valence states. Reproduced with permission [144]. Copyright 2025, Elsevier.

The separator in a Li‐ion cell is a porous membrane that prevents electrical shorts while allowing Li^+^ through. Conventional separators are polyolefin films (polyethylene, polypropylene). Recent research has explored functional separators coated or made from advanced materials to suppress dendrite growth, regulate ion transport, and improve safety [140, 141, 142]. MOF/2D hybrids are an emerging option, and such hybrids could help suppress metallic Li dendrites on high‐capacity anodes or block impurities. In Li–S batteries, a layered MOF@GO separator acted as a molecular sieve: the MOF's ∼0.9 nm pores allowed Li^+^ through but trapped larger polysulfide species. In a similar way, a MOF@GO separator in Li‐ion cells could enable size‐selective transport (e.g., permitting Li^+^ but blocking solvated ions or pulverized particles). For example, Qi et al. illustrated that a MOF@GO film has 9 Å pores that exclude large polysulfide anions [142]. By analogy, one could filter out larger unwanted species or regulate the electrolyte composition in Li‐ion cells. In another report, a functional separator based on defect‐rich (Ni/Fe‐MOF@rGO) hybrid is developed via an in situ approach to enhance Li–S battery performance (Figure 14a–f) [143]. Ni doping in Fe‐MOF increases the number of metallic active sites, boosts electrical conductivity, and enhances metal synergy, collectively improving redox kinetics. The combination of strong chemical interactions from defect‐rich MOFs and physical adsorption by rGO, enabled by in situ method, effectively suppresses the polysulfide shuttle effect. The Ni/Fe‐MOF@rGO‐PP separator enables remarkable cycling stability, delivering 403 mAh g^−1^ over 1250 cycles at 1.0 C. However, precise control over defect density and metal distribution during in situ synthesis remains challenging and may affect reproducibility and large‐scale fabrication.

Furthermore, Wang et al. developed 2D Ti_3_C_2_T_x_/UiO‐66‐NH_2_ hybrid separator via a room temperature in situ growth strategy [144], integrating the high conductivity of Ti_3_C_2_T_x_ with the porosity and microporous channels of UiO‐66‐NH_2_ (Figure 14g–k). Conducting the in situ growth at room temperature avoids harsh solvothermal conditions, thereby preserving the structural integrity and surface terminations of MXene while enabling scalable and energy‐efficient fabrication. This synergy effectively suppresses polysulfide shuttling while catalyzing the conversion of polysulfides. It delivered 1247 mAh g^−1^ at 0.1 C and maintained 0.04093% capacity decay per cycle over 1500 cycles. Notably, the physical blocking mechanism relies on the UiO‐66‐NH_2_ micropores (pore diameter∼1.35 nm) being larger than the Li^+^ ion but smaller than polysulfides (around 1.2–1.7 nm). However, the smallest polysulfides (Li_2_S_2_, Li_2_S_4_) can still be within or smaller than this range, indicating the imperfect physical blocking. In another report, Liu et al. demonstrate a thin Janus separator for Li–S batteries, featuring a conductive Ti_3_C_2_T_x_ MXene layer on the cathode side and a Cu‐TCPP MOF (TCPP: tetra(4‐carboxyphenyl) porphine) layer on the anode side [145]. The MXene layer, rich in polar groups, anchors polysulfides and enhances sulfur redox kinetics, while the MOF layer acts as an ionic sieve, blocks polysulfide migration, and promotes uniform lithium deposition to suppress dendrites. This dual‐function design simultaneously addresses the shuttle effect and anode instability, delivering high capacities (1275 mAh g^−1^ at 0.1 C), good cycling stability, low self‐discharge, and strong rate performance, even under high sulfur loading. These studies collectively demonstrate that integrating MOF/MXene hybrid sulfur hosts with functional separators synergistically enables chemical polysulfide confinement, catalytic conversion, and enhanced electronic conductivity, leading to markedly improved Li–S battery performance. Nevertheless, size‐selective physical blocking of small polysulfides remains incomplete, and long‐term stability under high sulfur loading and lean electrolyte conditions is yet to be established. Future designs must therefore emphasize on multifunctional interfaces that integrate strong chemical adsorption, catalytic activity, and robust ion selectivity to ensure durable, practical Li–S batteries.

#### 4.2.3 Sodium‐Ion Batteries (SIBs)

**FIGURE 15:**
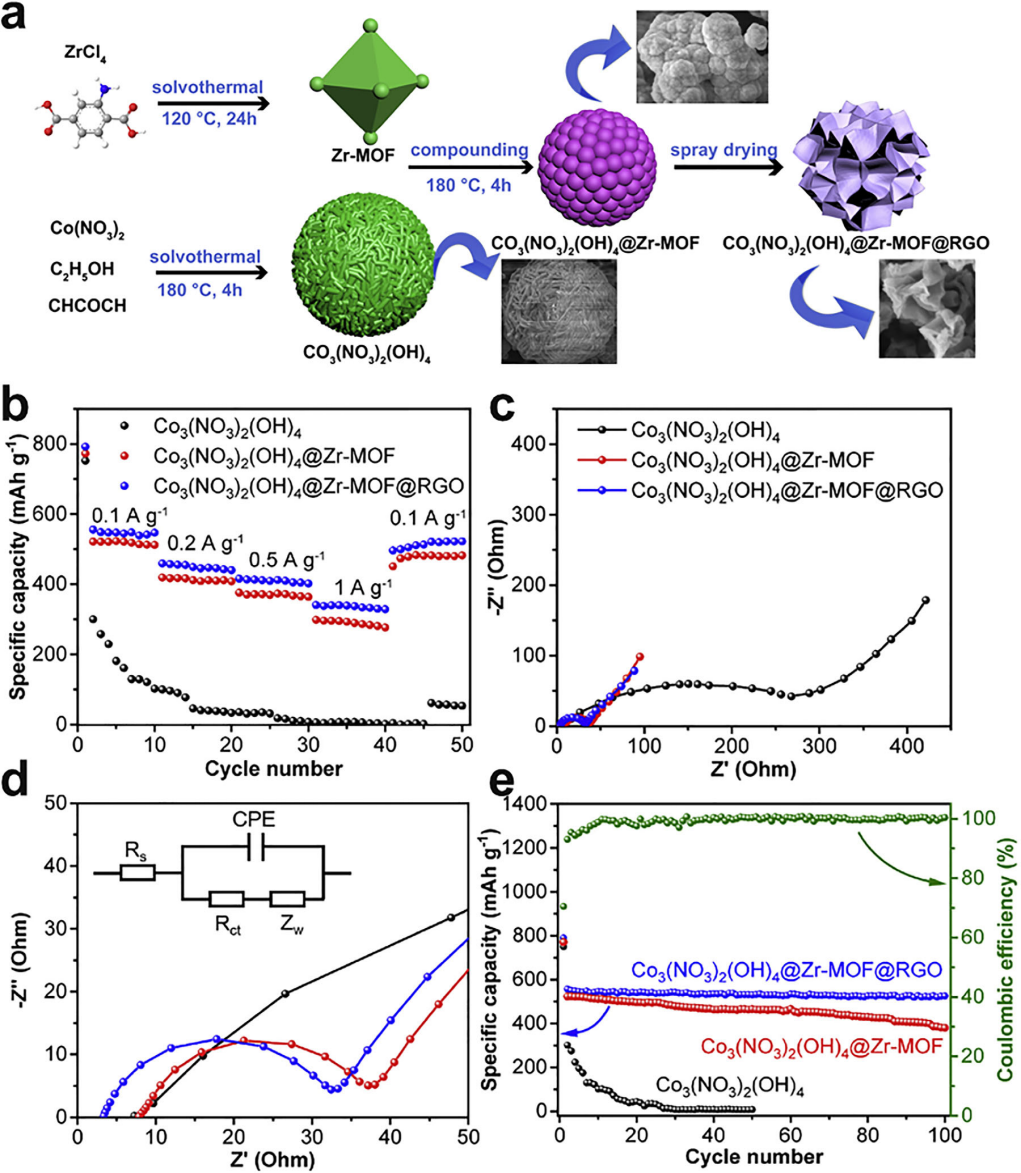
(a) Schematic illustration of synthesis design of Co_3_(NO_3_)_2_(OH)_4_‐Zr‐MOF@rGO, Electrochemical performance comparison of Co_3_(NO_3_)_2_(OH)_4_, Co_3_(NO_3_)_2_(OH)_4_‐Zr‐MOF, Co_3_(NO_3_)_2_(OH)_4_‐Zr‐MOF@rGO: (b) Rate capability, (c,d) Nyquist plot, and (e) Cycle life at 0.1 A g^−1^. Reproduced with permission [148]. Copyright 2020, Elsevier.

Sodium‐ion batteries (SIBs) are convincing alternatives to LIBs due to abundance of sodium and lower cost. However, the larger ionic radius of Na^+^ (1.02 Å) vs Li^+^ (0.76 Å) poses significant challenges for intercalation hosts, leading to sluggish kinetics and severe volume expansion [146, 147]. MOF‐2D materials hybrids offer a highly promising pathway to engineer electrodes and functional components that synergistically address the inherent hurdles of SIBs. For instance, Zhang et al. synthesized a “spitball‐like” composite in which Co_3_(NO_3_)_2_(OH)_4_ nanoclusters were formed on the outer surface of Zr‐based MOF particles that were in turn anchored on rGO sheets using an ex situ ultrasonication‐assisted approach [148]. The ex situ ultrasonication method preserves the intrinsic crystallinity of each component, while promoting uniform dispersion through physical anchoring. This hybrid architecture is employed as an anode, where rGO provided a flexible conductive network and the Co‐Zr MOF offered abundant redox‐active sites (Figure 15a). The resulting anode rendered a high capacity of 340 mAh g^−1^ at 1 A g^−1^ and exceptional cycling stability, retaining ∼87% capacity after 1000 cycles (Figure 15b–e). The enhanced performance was ascribed to the rapid Na^+^ transport through the MOF pores and the high electronic conductivity of the graphene matrix. However, because the interaction between MOF particles and rGO is primarily noncovalent, interfacial adhesion may be weaker than that achieved via in situ growth, potentially limiting long‐term structural robustness under prolonged cycling.

However, the CV curves of the final composite are remarkably similar to those of pure Zr‐MOF, with the characteristic peaks of Co_3_(NO_3_)_2_(OH)_4_ almost fully suppressed. This indicates that Zr‐MOF and rGO form a continuous coating, which may restrict access to the active Co_3_(NO_3_)_2_(OH)_4_ material, and cause the electrochemical behavior to rely heavily on the surface and pseudocapacitive contributions of the composite layers. In another example, a fluorine‐doped cobalt‐based MOF (F‐Co‐MOF) with hollow urchin‐like nanostructure was in situ self‐assembled on rGO via a facile solvothermal method to enhance electrochemical performance in LIBs and SIBs [149]. Benefiting from the synergistic effects of F‐Co‐MOF and rGO, the composite exhibited a high reversible capacity of 1202 mAh g^−1^ at 0.1 A g^−1^ for LIBs, with 97.6% capacity retention over 550 cycles. In SIBs, the material achieved 181.6 mAh g^−1^ at 0.1 A g^−1^. The enhanced performance is attributed to improved conductivity, structural stability, and pseudocapacitive contribution from F doping. However, the solvothermal in situ process may suffer from limited control over nucleation density and crystal size, which can lead to nonuniform MOF growth or excessive loading if not carefully regulated. The relatively lower performance in SIBs may be attributed to the larger ionic radius of Na^+^, leading to greater resistance and slower diffusion kinetics during cycling.

Overall, these examples highlight that MOF/rGO hybrids can effectively improve Na^+^ storage by enhancing electronic conductivity, providing accessible redox sites, and mitigating structural degradation. Nevertheless, incomplete utilization of active components and intrinsically sluggish Na^+^ diffusion, especially compared to Li^+^ systems, still limit achievable capacity and rate performance. Future efforts must be devoted to designing an open framework with improved interfacial exposure and tailored pore/chemistry engineering to fully accommodate the larger Na^+^ ion in practical SIBs.

**FIGURE 16:**
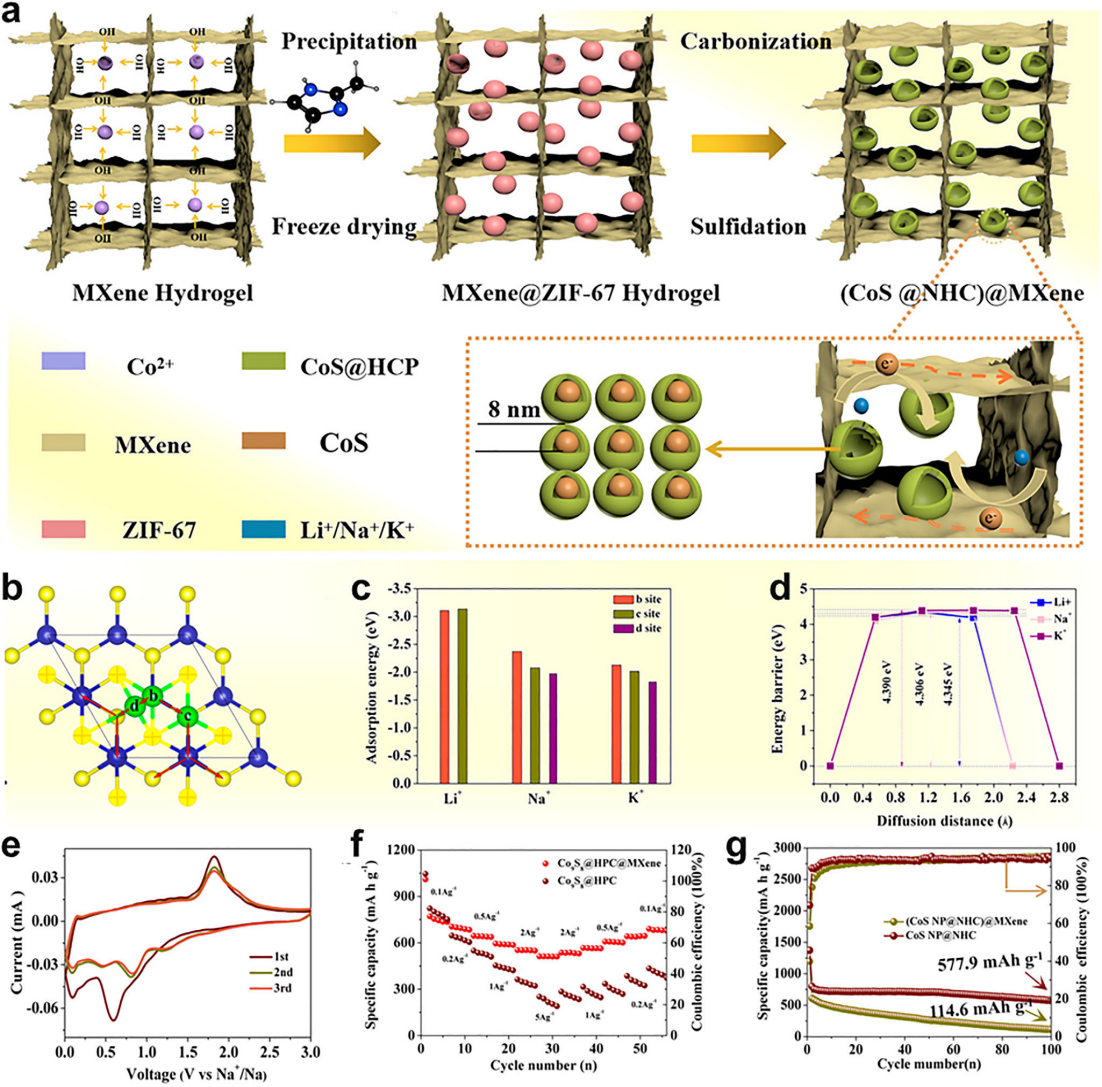
(a) Graphical illustration depicting carbonization and sulfidation process involved in the synthesis of CoS@NHC@MXene, (b–d) Adsorption sites for Li^+^, Na^+^, K^+^ ions on CoS material as determined by CINEB calculations, along with the corresponding adsorption energies and energy barrier variations, (e) CV curves of CoS@NHC@MXene at 0.1 mV s^−1^, Electrochemical performance comparison of CoS@NHC@MXene and CoS@NHC for SIBs: (f) Rate performance, and (g) Cyclic stability at 0.1 A g^−1^. Reproduced with permission [150]. Copyright 2021, American Chemical Society.

Furthermore, 3D ZIF‐67@MXene aerogel was developed through in situ growth of ZIF‐67 on a self‐assembled MXene hydrogel [150]. Co^2+^ ions coordinate with oxygen‐containing groups on Ti_3_C_2_T_x_ MXene, inducing gelation; subsequent MOF crystallization yields a hierarchically porous, interconnected conductive network that effectively prevents MXene sheet restacking. To demonstrate its potential, the MOF@MXene aerogel is converted via carbonization‐sulfidation into a porous CoS nanoparticle‐embedded, N‐doped carbon/MXene composite. Structural coupling between CoS, carbon, and the MXene network enhances electrochemical conductivity, buffers volume changes, and facilitates stable charge storage. This architecture ensures fast electron pathways, abundant active sites, and improved ion accessibility (Figure 16a). As anodes for Li‐, Na‐, and K‐ion batteries, the materials exhibit outstanding performance corroborated via CINEB calculations. (Figure 16b–d). For SIBs, it rendered high capacity of 420 mAh g^−1^ at 2 A g^−1^ after 650 cycles and excellent rate performance, even at high mass loadings, with high volumetric energy densities (Figure 16e–g). Notably, the first‐principle calculations indicate that the diffusion energy barriers for Na^+^, Li^+^, and K^+^ ions on the CoS surface (∼4.3–4.5 eV) are about twice the corresponding adsorption energy, indicating a relatively low ionic surface diffusivity. Therefore, future research should aim to engineer MOF‐derived chemistry and interfaces to lower diffusion barriers while preserving structural integrity under long‐term cycling.

In another report, in situ assembly of MOF‐derived Zn, Co, N, and C dopants into a Ti_3_C_2_T_x_ MXene framework were designed, where MXene serves as a highly conductive host, while the MOF‐derived elements act as sodiophilic sites that uniformly guide sodium ion deposition and suppress volume expansion [151]. This synergistic integration achieves exceptional performance, displaying 99.99% coulombic efficiency after 3000 cycles, and stability beyond 5000 h in symmetric cells. The blend of conductive structure and sodiophilic chemistry establishes a robust and efficient Na‐metal anode.

Interestingly, MOF‐derived 2D material can also be a potential anode material for SIBs. For instance, MoTe_2_ nanosheets are uniformly coated on MoO_2_/N‐doped carbon (NC) rods (denoted MoTe_2_@MoO_2_/NC) using a Mo‐MOF sacrificial template via high‐temperature solid‐phase synthesis [152]. This MOF‐derived strategy enables precise compositional control, uniform heterostructure formation, and effective interfacial contact between the metal chalcogenide, oxide, and carbon phases. In this architecture, the MoO_2_ core provides a highly conductive backbone and mitigates the volume expansion issue of MoTe_2_, while the MoTe_2_ shell ensures short ion diffusion paths. Meanwhile, the N‐doped carbon matrix enhances overall electrical conductivity and maintains structural integrity. This synergy yields impressive SIB performance with a specific capacity of 463.9 mAh g^−1^ at 5 A g^−1^ and decent cyclic stability. The material's base component, MOTe_2_, undergoes intense π–π interactions and high surface energy, resulting in restacking and agglomeration of 2D structures during cycling, which severely impede Na^+^ diffusion and degrade SIB performance. Hence, advanced interface and structural engineering strategies are essential to suppress agglomeration and fully exploit the high‐rate potential of MOF‐derived 2D anodes.

By contrast, examples of MOF‐2D hybrids used as SIB cathodes are still emerging. Conventional SIB cathodes rely on layered oxides, polyanionic phosphates, or organic insertion hosts. Pure MOFs as cathode materials are relatively rare, because few MOFs have sufficiently high operating potentials and redox stability. Some related work comes from other battery chemistries: for instance, Prussian blue analogs (PBAs), which are open‐framework metal cyanides often classified as MOFs, have been coated or composited with graphene to enhance rate performance in SIBs. While PBAs like Na_2_FeFe(CN)_6_ are inherently 3D frameworks, adding graphene can alleviate conductivity issues. However, true MOF/graphene cathodes in SIBs (beyond PBAs) are not widely reported. One study used a layer of ZIF‐8 to coat a Prussian blue cathode and observed that it protected the cathode structure, but this did not directly involve 2D materials [153]. MOF‐2D hybrids can show a promising application as a cathode material for SIBs.

#### 4.2.4 Zinc‐Based Batteries

Zinc‐ion batteries provide safe, low‐cost aqueous energy storage with high theoretical capacity, yet cathode kinetics and stability limit performance [154, 155]. An alternately stacked Cu‐HHTP/V_2_CT_x_ hybrid with a 2D sandwich‐like architecture was synthesized using self‐assembly method, producing molecular‐scale hybridization between Cu‐HHTP nanosheets and V_2_CT_x_ MXene [156]. This self‐assembly strategy allows controlled layer‐by‐layer stacking, strong interfacial contact, and efficient charge transport across the hybrid interface. This architecture furnishes abundant electroactive sites, enhanced electrical conductivity, and superior structural stability, enabling reversible Zn^2+^ intercalation as confirmed by ex situ characterization and theoretical calculations. These results underscore MOF/MXene hybrids as promising, scalable cathodes for high‐performance zinc‐ion batteries. The necessity to modify the MXene (PDAA‐MX) to achieve the required electrostatic self‐assembly with the Cu‐HHTP introduces an additional processing step, underscoring the inefficiency of direct mixing and the requirement for complex processing to fully harness the synergistic potential. This highlights that while self‐assembly offers precise interfacial control, it often requires more elaborate processing to fully exploit the synergistic potential of MOF/MXene hybrids.

Metal‐air batteries (e.g., Zn‐air, Li‐air) demand bifunctional electrocatalysts for ORR and OER [157, 158, 159]. MOF/2D hybrids can serve as low‐cost catalysts where MOFs offer abundant active metal sites or templates for metal oxides, while 2D carbons provide conductivity and surface area. Zheng et al. report a surfactant‐assisted synthesis of ultrathin bimetallic CoNi‐MOF nanosheets grown in situ on rGO, producing CoNi‐MOF/rGO hybrids with abundant accessible active sites and improved electronic conductivity [160]. MOF‐rGO hybrid improves charge transfer and stability, yielding superior bifunctional oxygen electrocatalysis (OER/ORR) in alkaline media. In the same line, a bimetallic Zn/Co ZIF microcrystal was grown on GO sheets via in situ growth [161]. Compared to pristine ZnCo‐ZIF, the hybrid showed significantly improved ORR and OER activity in alkaline media. Utilizing ZnCo‐ZIF@GO as the air cathode in ZABs, the device exhibited high charge/discharge efficiency, energy density, and cycling stability. The enhanced activity was attributed to (i) synergistic effects between ZnCo‐ZIF and GO (improved conductivity and dispersion), (ii) hierarchical porosity facilitating O_2_ transport, and (iii) possibly electronic interactions at the MOF‐graphene interface. Notably, the specific surface area and pore volume of the ZnCo‐ZIF/GO hybrid are decreased compared to the pure ZnCo‐ZIF, attributed to the tight connection of GO with ZIF and the encapsulation of ZIF particles by the GO sheets. Future catalyst designs must therefore balance electronic synergy with preserved porosity to fully exploit MOF‐based active sites in practical metal‐air batteries.

In addition, MXene can also be incorporated into MOFs to get a hybrid structure, which can show promising results in ZABs. For instance, the bi‐ligand ZIF‐67/MXene hybrid was designed using in situ method followed by pyrolysis to yield carbonized catalyst (b‐CZIF‐67/MXene)  for high‐performance Zinc‐based batteries [162]. The in situ growth ensures strong interfacial contact between ZIF‐67 and MXene, while the subsequent pyrolysis converts the MOF into an active conductive framework rich in catalytic sites. The MXene backbone enhances conductivity, while the bi‐ligand ZIF‐67 (using 2‐methylimidazole and 2‐aminobenzimidazole) prevents structural collapse during pyrolysis, exposing abundant Co‐N_x_ active sites. The catalyst exhibited a specific capacity of 550.6 mAh g^−1^ and a high power density of 117.5 W kg^−1^, outperforming Pt/C. The hierarchical structure ensures efficient mass/charge transfer, making it a durable, cost‐effective cathode material for rechargeable ZABs. However, the calculated conductivity of the b‐CZIF‐67/MXene composite is only 1.5 S cm^−1^, which is significantly lower than the conductivity of the pristine MXene (10.1 S cm^−1^), indicating the insulating pyrolyzed ZIF component still attenuates the overall conductivity. Consequently, future optimization should therefore focus on preserving electronic pathways while maintaining high active‐site density to fully exploit the advantages of the MOF/MXene hybrid. Furthermore, Table 3 provides a comprehensive summary of the electrochemical performance of MOF‐2D material hybrids across various battery systems, highlighting the corresponding key innovations and the limitations identified in each study.

**TABLE 3:** Electrochemical performance comparison of different MOF‐2D materials hybrid for different battery applications.

Material	Synthesis method	Electrode	Capacity	Cycle life (RC/R/CN)	Key innovation	Limitation	Refs.
Li‐ion batteries							
Al‐MOF/Graphene	Ex situ method	Anode	400 mAh g^−1^ (0.1 A g^−1^)	484/500/1000	Synergistic structure	Side irreversible reactions	[130]
Ni‐MOF/GO	In situ method	Anode	858.2 mAh g^−1^ (0.05 A g^−1^)	740/50/100	Rapid electron transfer	Complex synthesis	[131]
Cu_3_(BTC)_2_@GO	Layer‐by‐Layer method	Anode	1420 mAh g^−1^ (0.1 A g^−1^)	1296/100/100	“Pore‐cage‐pore” structure	Agglomeration issue	[53]
Ti_3_C_2_T_x_/NiCo‐MOF	In situ method	Anode	1704 mAh g^−1^ (0.1 A g^−1^)	637/200/200	Stable Interface	Synthesis Complexity	[39]
Co‐MOF/V_2_CT_x_	In situ method	Anode	1029.7 mAh g^−1^ (0.1 A g^−1^)	484.3/100/120	In situ growth	Poor stability	[40]
MXene@Sn‐MOF	In situ method	Anode	1002 mAh g^−1^ (0.1 A g^−1^)	1009/100/100	3D conductive network	Low charge storage contribution of MXene	[132]
CoNi‐MOF@MXene	In situ hydrothermal method	Cathode	820 mAh g^−1^ (0.1 A g^−1^)	615/200/100	Bimetallic 2D MOF heterostructure	Poor ion transport	[133]
**Li–sulfur batteries**							
In‐IPA@rGO	Ex situ hydrothermal method	Cathode	1672 mAh g^−1^ (0.2 C)	898/0.2 C/100	Inhibits shuttle effect	Capacity decay	[137]
MIL‐53/rGO	Ex situ hydrothermal method	Cathode	1250 mAh g^−1^ (0.1 C)	601/1C/400	3D hierarchical structure	Shuttle effect	[138]
nMOF‐867/Ti_3_C_2_T_x_	In situ solvothermal method	Cathode	1302 mAh g^−1^ (0.2 C)	624/0.2 C/500	Polysulfide trapping	Thermal stability issue	[139]
Ti_3_C_2_T_x_/UiO‐66‐NH_2_	In situ method	Separator	1247 mAh g^−1^ (0.1 C)	—	In situ growth	Imperfect physical blocking	[144]
Cu‐TCPP MOF/MXene	—	Separator	1275.5 mAh g^−1^ (0.1 C)	313/0.5 C/350	Anisotropic spatial heterogeneity	Increased energy barrier for sulfur conversion	[145]
**Sodium‐ion batteries**							
Co_3_(NO_3_)_2_(OH)_4_@Zr‐MOF@rGO	Ex situ method	Anode	340 mAh g^−1^ (1 A g^−1^)	523/100/100	Spitball‐type architecture	Limited active material exposure	[148]
F‐Co‐MOF/rGO	In situ solvothermal method	Anode	354.5 mAh g^−1^ (0.1 A g^−1^)	181.6/100/100	Hollow urchin nanostructure	Slow diffusion of Na^+^	[149]
(CoS NP@HC)@MXene	Thermal induced carbonization and sulfidation method	Anode	734.5 mAh g^−1^ (0.1 A g^−1^)	577.9/100/100	Self‐assembly	Low ionic surface diffusivity	[150]
ZC14NC@Ti_3_C_2_T_x_	In situ assembly method	Anode	110 mAh g^−1^ (3 C)	88/3C/500	Uniform Na deposition	Synthesis complexity	[151]
MoTe_2_@MoO_2_/NC	Template assisted synthesis method	Anode	1102.5 mAh g^−1^ (0.1 A g^−1^)	328.3/1000/100	Core‐shell structure	Sluggish Na^+^ ion diffusion	[152]
**Zinc‐based batteries**							
Cu‐HHTP/V_2_CT_x_	Self‐assembly method	Cathode	173.1 mAh g^−1^ (4 A g^−1^)	260.1/100/200	Sandwich‐like structure	Complex assembly	[156]
Co Ni‐MOF/rGO	In situ method	Cathode	711 mAh g^−1^ (5 mA cm^−2^)	—	In situ growth	Low performance	[160]
Zn Co‐ZIF@rGO	In situ method	Cathode	—	—	Bi‐metallic heterostructure	Low surface area	[161]
b‐CZIF‐67/MXene	In situ method	Cathode	550.6 mAh g^−1^ (5 mA cm^−2^)	—	Trifunctional electrocatalyst	Low conductivity	[162]

Abbreviations: RC‐ Reversible capacity (mAh g(1), R‐Rate (mA g(1), CN‐Cycles number.

#### 4.2.5 Supercapacitors

Supercapacitors offer long cycle life and high power density, bridging the gap between batteries and conventional capacitors [163, 164, 165]. MOF‐2D hybrids are ideal candidates, capitalizing on the high porosity and redox activity of MOFs together with the conductivity and surface area of 2D supports. Graphene can be incorporated into MOFs to develop electrode materials with good conductivity and electrochemical performance. For instance, Erçarıkcı et al. infused a flexible 3D graphene sponge (3DG) with a Mn/Co MOF using an ex situ approach to fabricate a quasi‐symmetric device [166]. The ex situ strategy enables straightforward MOF loading onto a preformed conductive scaffold, preserving the intrinsic porosity and mechanical flexibility of the 3DG framework while allowing scalable fabrication. The porous 3D carbon provided a scaffold for MOF deposition, creating a flexible and highly porous hybrid. The MnCo‐MOF/3DG electrode delivered an extremely high specific capacitance of 4086 F g^−1^ at 1 A g^−1^, retaining 83% capacity after 10,000 cycles. The assembled asymmetric device (MnCo‐MOF/3DG //3DG) achieved a high energy density of 198.5 Wh kg^−1^ at 5823 W kg^−1^. However, because the MOF is incorporated through physical infiltration rather than in situ growth, interfacial bonding between the MOF and graphene sponge may be relatively weak, potentially limiting charge‐transfer efficiency and long‐term structural robustness under prolonged cycling conditions.

**FIGURE 17:**
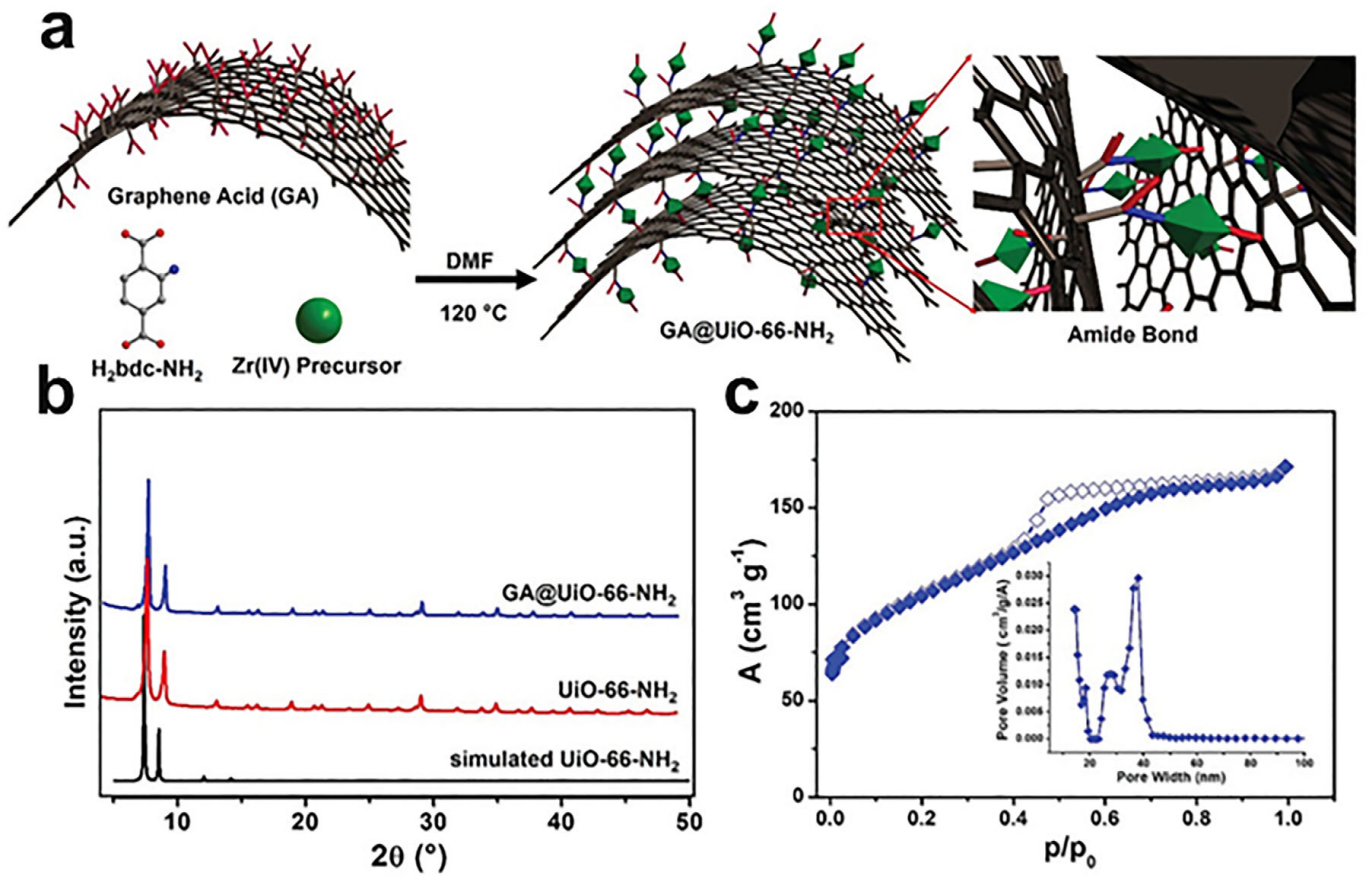
(a) Graphical depiction of covalent bonding formation (amide bond) in GA@UiO‐66‐NH_2_ hybrid structure, (b) PXRD of GA@UiO‐66‐NH_2_, UiO‐66‐NH_2_, and simulated UiO‐66‐NH_2_, (c) N_2_ adsorption‐desorption isotherm of GA@UiO‐66‐NH_2_ with inset of NLDFT pore size distribution, Reproduced with permission [76]. Copyright 2021, Wiley‐VCH.

Graphene‐based derivatives, such as functionalized graphene, promote interfacial interactions between MOF and give rise to a high‐performance electrode material. In this line, covalent linkage of amino‐functionalized UiO‐66 to carboxylated graphene (graphene acid (GA)) was designed via in situ approach facilitated by amide bonds [76]. The formation of robust covalent amide linkages ensures strong interfacial coupling and continuous electron‐transport pathways between the MOF and graphene support, while simultaneously providing abundant electroactive sites and enhanced ion accessibility (Figure 17a–c). The resulting GA@UiO‐66‐NH_2_ hybrid maintained a hierarchical porous structure and a π‐conjugated network between the MOF and GA. Because of the covalent bonding, this composite achieved excellent electrical connectivity. Compared with physically assembled hybrids, this covalent strategy minimizes interfacial resistance and suppresses material detachment during prolonged cycling. Using this composite as the positive electrode against a Ti_3_C_2_T_x_ MXene counter electrode, the assembled asymmetric supercapacitor reached an energy density of 73 Wh kg^−1^ at a power density of 16 kW kg^−1^, with 88% capacitance retention over 10 000 cycles. However, the requirement for precise surface functionalization and covalent coupling reactions increases synthetic complexity, which may limit large‐scale processing. Overall, this study highlights the importance of an interconnected conductive framework and the amide‐linked MOF/graphene hybrid provides both high surface area and fast charge transfer pathways, leading to high performance in an asymmetric supercapacitor. Another recent example is by Mousaabadi et al., who grew a bimetallic (Ni/Co) hemin‐based MOF directly on phosphorus‐doped reduced GO (PrGO) via in situ approach [167]. The P‐doped GO served both as a conductive support and as a dopant‐modified carbon. The Ni/Co‐hemin MOF formed uniformly on the PrGO, forming hybrid and exhibited “battery‐type” behavior and delivering a specific capacity of 963 C g^−1^ at 1 A g^−1^ (in a three‐electrode cell). When used in an asymmetric hybrid supercapacitor, the device operated over a 1.8 V window and achieved an energy density of 70.3 Wh kg^−1^ at 0.9 kW kg^−1^, while powering an LED clock for 42 min. The improved performance was attributed to the dual‐metal MOF, phosphorus doping, and strong interfacial contact. These studies demonstrate that chemically bonded MOF/graphene hybrids, particularly those with covalent or dopant‐assisted interfaces, can significantly enhance charge transport, ion accessibility, and electrochemical stability in supercapacitors. However, the complexity of interfacial chemistry and synthesis may limit scalability and cost‐effectiveness for practical applications. Hence, future research should aim to simplify fabrication while preserving strong interfacial coupling and high active‐site utilization.

**FIGURE 18:**
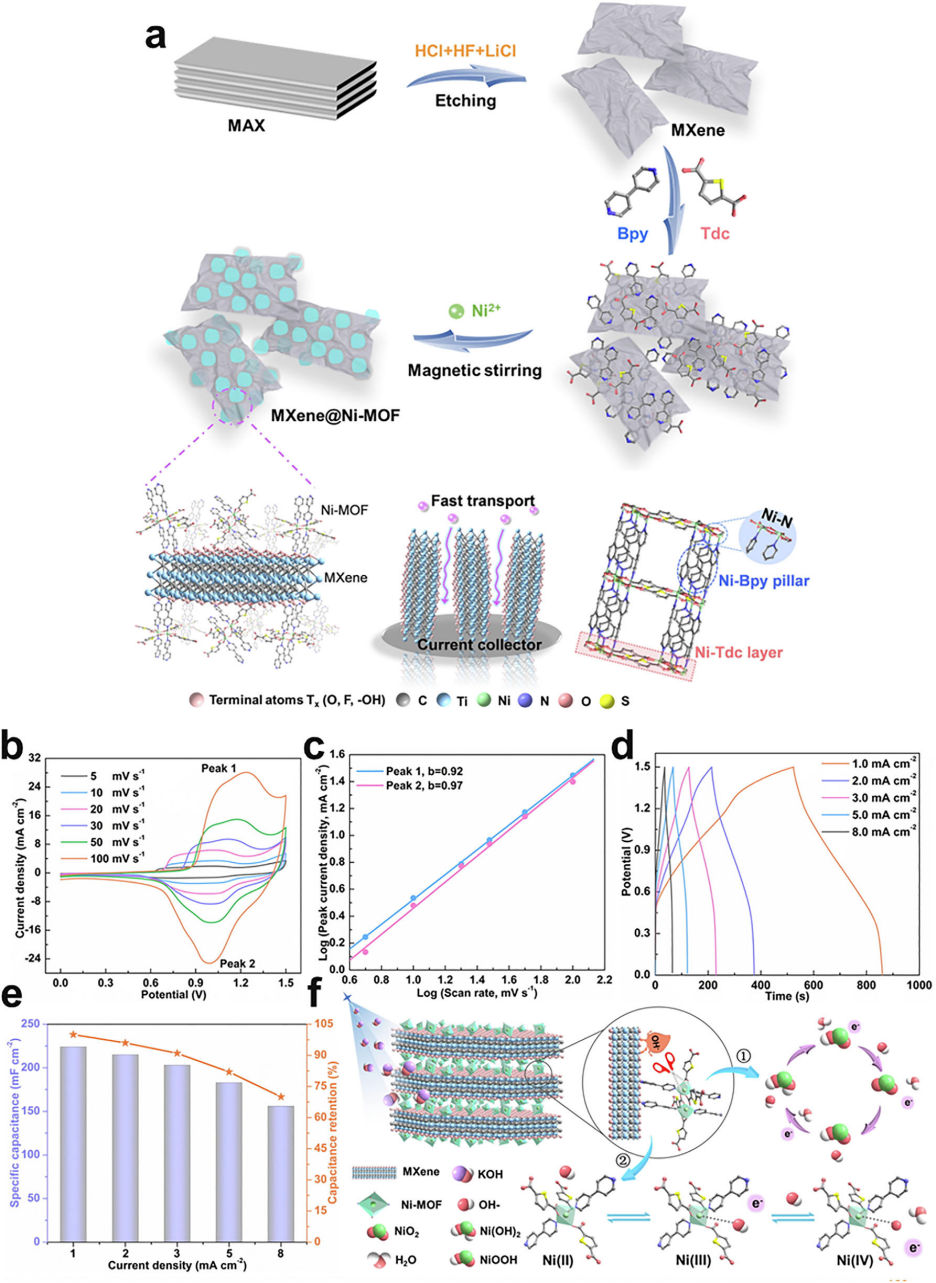
(a) Schematic illustration of the preparation of MXene@Ni‐MOF. Electrochemical performance of MXene@Ni‐MOF//AC device: (b) CV profiles, (c) Log (I) vs Log (b) plots, (d) GCD curves, (e) Specific capacitance, (f) Mechanism of charge/discharge of MXene@Ni‐MOF electrode. Reproduced with permission [168]. Copyright 2022, Elsevier.

Another common synthetic strategy is to mix or grow a MOF on MXene nanosheets. For instance, a pillared‐layer Ni‐MOF ([Ni(thiophene‐2,5‐dicarboxylate)(bipyridine)]_n_) was developed on T_i3_C_2_ MXene [168]. The in situ growth promotes strong chemical interactions between the MOF ligands and the surface terminations of MXene, effectively immobilizing the Ni‐MOF nanosheets and suppressing aggregation. The pillared MOF had an open Ni‐N framework conferring stability, and anchoring on MXene enabled fast electron transfer while preventing MOF aggregation (Figure 18a). This MXene@Ni‐MOF hybrid achieved a specific capacitance of 979 F g^−1^ at 0.5 A g^−1^, which is excellent. MXene acted as a conductive backbone and spacer, while MOF facilitates expanding layer spacing and avoids restacking. This architecture ensures fast electron pathways, abundant active sites, and improved ion accessibility (Figure 18b–f). A corresponding asymmetric supercapacitor exhibited outstanding cycle life. Similarly, a conductive 2D MOF (Ni‐HHTP, based on hexahydroxytriphenylene) with Ti_3_C_2_T_x_ MXene was designed via in situ hydrothermal approach [169]. They prepared a series of MXene@Ni‐HHTP composites by mixing exfoliated MXene with Ni‐HHTP nanosheets, and when used as the positive electrode in an aqueous asymmetric supercapacitor, it delivered a specific capacitance of 416.6 F g^−1^ at 0.5 A g^−1^. This shows that even moderate amounts of MOF loading on MXene can provide significant pseudo‐capacitance. In another striking example, Nb‐MOF/V_2_CT_x_ hybrid incorporated with graphene quantum dots (GQDs) via direct mixing, forming a hierarchical 2D/3D hybrid that couples Nb‐MOF pseudocapacitance with V_2_CT_x_ metallic conductivity and GQD‐facilitated charge transfer [170]. The direct mixing strategy enables a simple and scalable fabrication route while preserving the intrinsic properties of each component. The as‐designed hybrid has improved electron/ion transport, enlarged accessible redox sites, and suppressed MXene restacking, producing markedly boosted energy storage performance. The asymmetric device (Nb‐MOF/V_2_CTx@GQDs//AC) delivered an energy density of 59 Wh kg^−1^ at 1800 W kg^−1^. The device exhibits moderate cycling durability, retaining 81.2% of its capacity after 12 000 cycles. Nevertheless, the reliance on direct physical mixing may lead to suboptimal interfacial integration among the MOF, MXene, and GQDs compared to chemically coupled or in situ‐grown architectures, thereby constraining interfacial stability during prolonged cycling.

In another interesting work, 3D MOF was directly grown on Ti_3_C_2_T_x_ MXene nanosheets via an in situ coprecipitation strategy and subsequently to derive hollow Ti_3_C_2_T_x_/ZIF‐67/CoV_2_O_6_ architectures via an ion‐conversion‐exchange step [171]. This multistep strategy enables intimate interfacial coupling and controlled morphological transformation, yielding hollow architectures with abundant accessible active sites. This hierarchical design prevents MXene restacking, increases accessible surface area, and couples MXene metallic conductivity with MOF‐derived pseudocapacitance to enhance electron/ion transport. Ti_3_C_2_T_x_/ZIF‐67/CoV_2_O_6_ electrode delivered specific capacitance of 285.5 F g^−1^ at 1 A/g (24.3 F g^−1^ for ZIF‐67 and 129.5 F g^−1^ for Ti_3_C_2_T_x_/ZIF‐67). The composite also exhibits robust cycling (94.4% coulombic efficiency after 4000 cycles), underscoring the promise of MXene/MOF‐derived hollow heterostructures for high‐performance supercapacitors. However, the multi‐step synthesis involving coprecipitation and ion‐exchange increases processing complexity and may pose challenges for large‐scale production, highlighting the need for simplified yet controllable fabrication routes.

Notably, MOF/MXene hybrids can markedly enhance supercapacitor performance by suppressing MXene restacking and introducing pseudocapacitive redox sites; the gains are highly architecture‐dependent rather than intrinsic. However, performance strongly depends on MOF loading, interfacial chemistry, and structural hierarchy, with excessive MOF content potentially hindering conductivity and ion transport. Rational control over composition and architecture will therefore be essential to balance charge transport and active‐site utilization for scalable, high‐performance MXene/MOF‐based supercapacitors.

**FIGURE 19:**
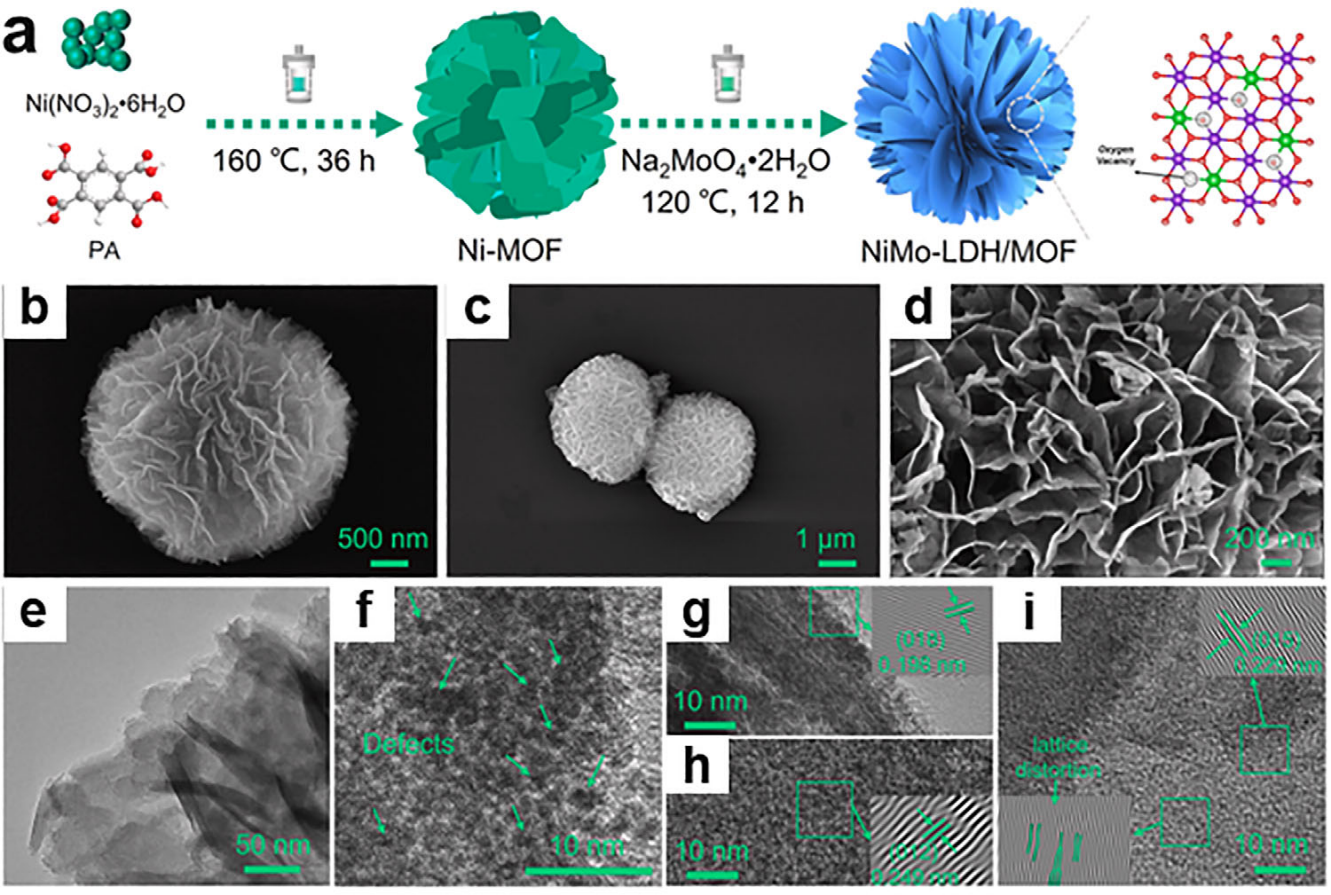
(a) Schematic design of synthesis pathway for the NiMo‐LDH/MOF hybrid; (b–d) SEM micrographs of Ni‐MOF and NiMo‐LDH/MOF hybrid, respectively, (e–i) HRTEM image of NiMo‐LDH/MOF hybrid, Reproduced with permission [175]. Copyright 2025, Elsevier.

LDHs offer high pseudocapacitance, tunable composition, and layered ion pathways but suffer low electrical conductivity, limited surface area, and structural instability (restacking), which reduce rate capability and cycle life [172, 173]. Hybridizing with MOFs enhances conductivity, porosity, exposed redox sites, and structural robustness, improving capacity, rate performance, and durability. For example, facile in situ solvothermal synthesis of NiCo‐MOF@LDH hybrid nanosheets designed for supercapacitor applications [174]. The hybrid material integrates the high surface area and tunable porosity of NiCo‐MOF with the excellent electrochemical activity and layered structure of LDHs. This synergy enhances ion diffusion, electrical conductivity, and structural stability, resulting in improved specific capacitance (1879 F g^−1^ at 0.5 A g^−1^), rate capability, and cycling durability. In another study, NiMo‐LDH/MOF‐1 nanoflowers with oxygen‐vacancy‐rich, defect‐engineered nanosheets were synthesized via a two‐step solvothermal process using Ni‐MOF as a template [175]. This heterostructure facilitates charge transfer kinetics owing to its abundant electrochemically active sites (Figure 19a–i). MOF prevents LDH self‐stacking, forms mesoporous channels, and improves ion transport, while oxygen vacancies enhance conductivity, active site density, and electronic structure. DFT studies confirm reduced bandgap and improved charge carrier mobility. The optimized electrode delivered a decent energy density of 58.6 Wh kg^−1^ and shows a high cycle life with 83.8% retention over 9000 cycles in asymmetric supercapacitors. The resultant defect‐engineered hybrid structures show a slight reduction in specific surface area, attributed to the partial dissolution of the Ni‐MOF crystal surface during the synthesis process. Another striking example demonstrates the “bottle‐around‐ship” type synthesis of a NiV LDH@ZIF‐67 p–n heterojunction using an in situ crystallization method [176]. This approach ensures effective interfacial contact and the formation of a well‐defined p–n heterointerface, which is difficult to achieve through physical mixing. In an asymmetric device with activated carbon, NiV LDH@ZIF‐67//AC achieves an energy density of 42.3 Wh kg^−1^ at 520.6 W kg^−1^, and shows capacity retention with 120% retention over 5000 cycles (compared to 81.4% for NiV LDH//AC). The superior energy and cycling behavior are ascribed to the p‐n heterointerface, which enhances electronic conductivity, exposes additional redox‐active sites, and facilitates ion diffusion and charge transfer, supporting durable high‐energy devices integration. However, ZIF‐67 contributes negligible intrinsic charge storage, resulting in an overall capacitance that remains lower than that of pristine NiV‐LDH, highlighting a trade‐off between interfacial engineering and active material utilization. Overall, these examples show that MOF/LDH hybrids can effectively mitigate the intrinsic conductivity and restacking limitations of LDHs by introducing hierarchical porosity, defect chemistry, and favorable interfacial electronic structures. However, performance gains are often offset by reduced surface area, partial MOF dissolution, or negligible charge contribution from certain MOFs, indicating that not all MOF components are electrochemically active. Therefore, future progress should aim at designing MOF/LDH architectures where both phases contribute meaningfully to charge storage while preserving conductivity and structural integrity under long‐term cycling.

TMDs like MoS_2_, WS_2_, VS_2_ offer layered structures and potential pseudocapacitance (especially at edges/defects), but suffer from poor conductivity and restacking [177, 178]. MOFs help overcome these limitations. This study presents a composite electrode, MoS_2_@Ni‐MOF formed by intercalating accordion‐like MoS_2_ into Ni‐MOF layers using an in situ hydrothermal approach, connected via sulfur bridges [179]. The hybrid combines structural strengths of both components, preserving the integrity of the skeleton. When assembled into an asymmetric supercapacitor (MoS_2_@Ni‐MOF//activated carbon), it achieves an impressive energy density of 72.93 Wh kg^−1^ at 375 W kg^−1^, demonstrating its high promise for an advanced supercapacitor. Similarly, Ali et al. designed a hierarchically structured Co‐MOF@WS_2_ composite supported on nickel foam using ex situ hydrothermal strategy and used it as a high‐performance electrode material for supercapacitors and achieved an energy density of 80.1 Wh kg^−1^ at 1008 W kg^−1^ [180]. Furthermore, it demonstrated cycling stability, retaining 83.6% of its capacitance after 12 000 cycles. This poor cyclic stability highlights the critical role of the MOF‐TMDs interface because, in the hybrid system, it initially facilitates a rapid charge storage process. However, repeated volume changes (expansion and contraction) can weaken the adhesion and disrupt the electrical contact with MOF and TMDs, and consequently isolate redox active sites, resulting in capacity decay. Another study introduces a novel binder‐free electrode NiCo‐MOF@WS_2_@NiVS synthesized, where via hydrothermal intercalation onto nickel vanadium sulfide (NiVS), WS_2_ nanoflakes and NiCo‐MOF were combined using physical mixing, thereby cherishing the synergy of MOFs with TMDs and sulfides [181]. This hybridization strategy enables straightforward fabrication while eliminating inactive binders, which improves electrical connectivity and active material utilization. This hybrid exhibited markedly enhanced electrochemical properties, rendering a high specific capacity of 1235 C g^−1^, outperforming NiCo‐MOF (567 C g^−1^) and WS_2_ (717 C g^−1^). The performance enhancement arises from synergistic redox activity, shortened ion‐diffusion pathways, and improved charge‐transfer kinetics enabled by the heterogeneous interfaces. However, as the hybrid is assembled primarily through physical mixing, the interfacial coupling is likely less robust, which may contribute to gradual performance decay, as evidenced by the retention of 88% of the initial capacity after 5000 cycles.

MOF/TMD hybrids effectively leverage the complementary advantages of both components to enhance pseudocapacitance, conductivity, and energy density in supercapacitors. However, the long‐term stability of these systems remains strongly dependent on the robustness of the MOF‐TMD interface, as repeated volume changes can degrade interfacial adhesion and electrically isolate active sites. Consequently, future efforts should emphasize interfacial engineering and mechanical compliance to sustain charge transport and cycling durability alongside high energy performance.

#### 4.2.6 Hybrid Ion Capacitors

**FIGURE 20:**
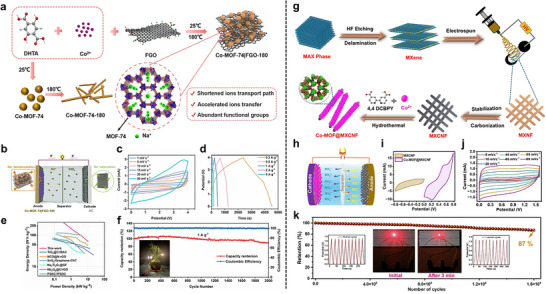
(a) Graphical representation of the design of Co‐MOF‐74/FGO hybrid highlighting the diffusion of Na^+^ ions, (b) Fabrication of Co‐MOF‐74/FGO‐180//AC SIHC device, Electrochemical performance of SIHC device: (c) CV profiles, (d) GCD curves, (e) Ragone plot with previously reported literature, (f) Cyclic stability and coulombic efficiency at 1 Ag^−1^ Reproduced with permission [184]. Copyright 2023, Elsevier. (g) Schematic representation of the synthesis of Co‐MOF/MXCNF hybrid; (h) Graphical illustration of AIHSC device; (i) Cumulative CV curves of cathode and anode; (j) CV curves of AIHSC at varying scan rates, and (k) Cyclic stability test at 0.1 A g^−1^ with the inset of initial and last 10 cycles and demonstration of glowing LED. Reproduced with permission [185]. Copyright 2025, The Royal Society of Chemistry.

Hybrid ion capacitors combine the high power density of supercapacitors with the high energy density of batteries by pairing a faradaic electrode with a capacitor‐type electrode, enabling long cycle life and balanced energy‐power performance [182, 183]. Incorporating MOF/2D‐material hybrids as electrodes can be a promising option to achieve high‐performance energy storage technology. For instance, a honeycomb‐structured Co‐MOF‐74 anchored on functionalized graphene oxide (FGO) was developed via in situ hydrothermal method as an advanced anode for sodium‐ion hybrid capacitors (SHICs) [184]. The in situ growth enables uniform MOF anchoring and strong interfacial coupling with FGO, facilitating efficient electron transport and structural stability. The hybrid material combines the high porosity and active sites of Co‐MOF‐74 with the conductivity of FGO, enabling efficient Na^+^ diffusion and electron transfer (Figure 20a). The assembled SHIC device (Co‐MOF‐74/FGO‐180/AC) achieves 240 Wh kg^−1^ energy density and 10 kW kg^−1^ power density, outperforming many reported SHICs (Figure 20b–f). The design leverages MOFs' structural advantages for high‐performance energy storage, offering a promising route for next‐generation hybrid capacitors. However, low coulombic efficiency due to the initial high irreversible capacity is observed. This is attributed to the decomposition of the electrolyte and the formation of the solid electrolyte interphase (SEI). In another notable example, an advanced heterostructure was developed for ammonium‐ion supercapacitors by integrating an oxygen‐rich Co‐MOF with MXene nanofibers (MXCNF) via in situ hydrothermal approach (Figure 20g) [185]. The Co‐MOF features a unique (O_4_‐CoN_2_) coordination geometry, enhancing stability and electrochemical performance. The resulting Co‐MOF@MXCNF heterostructure exhibited a high specific capacitance of 980 F g^−1^ at 1 A g^−1^ and retained 91.1% of its initial capacitance after 16 000 cycles (Figure 20h–k). However, the Co‐MOF/MXCNF hybrid exhibits poor coulombic efficiency due to the partial entrapment of NH_4_^+^ ions within the MOF structure, which impedes their deintercalation process during the discharging process.

These studies highlight the potential of MOF‐2D material hybrids to deliver high energy and power densities in hybrid ion capacitors through synergistic redox activity and rapid charge transport. Nevertheless, their performance is often constrained by a kinetic imbalance between the faradaic and capacitive electrodes, which can lead to suboptimal charge utilization and voltage mismatch during long‐term cycling. Future designs must therefore emphasize regulating interfacial chemistry and ion‐storage reversibility to balance high energy‐power output with efficient and stable cycling.

Moreover, Table 4 presents a detailed overview of the electrochemical performance of MOF‐2D material hybrids developed for supercapacitor applications, systematically outlining the key innovations employed, as well as the associated performance advantages and inherent limitations reported in each study.

**TABLE 4:** Electrochemical performance comparison of different MOF‐2D materials hybrid for supercapacitor applications.

Material	Synthesis method	Capacitance	Energy density	Power density	Cycle life	Innovation	Limitation	Refs.
**Supercapacitors**								
MnCo‐MOF/3DG	Ex situ method	4086 F g^−1^ (1 A g^−1^)	198.5 Wh kg^−1^	5.8 kW kg^−1^	92.0% (10 000 cycles)	Good Flexibility	Limited ion diffusion	[166]
GA@UiO‐66‐NH_2_	In situ method	651 F g^−1^ (2 A g^−1^)	73 Wh kg^−1^	16 kW kg^−1^	88.0% (10 000 cycles)	Covalent assembly via amide linkage	Complex synthesis	[76]
PrGO/NiCo‐MOF	In situ method	963 C g^−1^ (1 A g^−1^)	70.3 Wh kg^−1^	0.9 kW kg^−1^	86.7% (5000 cycles)	Good conductivity	Cyclic stability issue	[167]
MXene@Ni‐MOF	In situ method	979 F g^−1^ (0.5 A g^−1^)	—	—	98.0% (5000 cycles)	Pillared‐layer structure	Limited cycle life demonstration	[168]
MXene@Ni(HHTP)	In situ method	416 F g^−1^ (0.5 A g^−1^)	53.7 Wh kg^−1^	1.5 kW kg^−1^	95.0% (20 000 cycles)	Synergistic structure	Low capacitance	[169]
Nb‐MOF/V_2_CTx@GQDs	Direct mixing method	2742 C g^−1^ (2 A g^−1^)	59 Wh kg^−1^	1.8 kW kg^−1^	81.2% (12 000 cycles)	Synergistic heterostructure	Coulombic efficiency issue	[170]
Ti_3_C_2_T_x_/ZIF‐67/CoV_2_O_6_	In situ coprecipitation method	285.5 F g^−1^ (1 A g^−1^)	36.27 µWh/cm^2^	0.75 mW/cm^2^	94.4% (4000 cycles)	Ion‐conversion exchange strategy	Complex synthesis	[171]
NiCo‐MOF@LDH	In situ solvothermal method	1873.9 F g^−1^ (0.5 A g^−1^)	49.8 Wh kg^−1^	0.4 kW kg^−1^	83.0% (10 000 cycles)	Interconnected porous structures	Poor cyclic stability	[174]
NiMo‐LDH/MOF‐1	Template‐assisted method	1879 F g^−1^ (0.5 A g^−1^)	58.6 Wh kg^−1^	0.8 kW kg^−1^	83.8% (9000 cycles)	Defect‐rich nanoflower structure	Low surface area due to dissolution of MOF crystal	[175]
NiV LDH@ZIF‐67	In situ crystallization method	830.6 F g^−1^ (1 A g^−1^)	42.3 Wh kg^−1^	0.5 kW kg^−1^	120.0% (5000 cycles)	Unique p‐n heterojunction interface	Negligible charge storage contribution of ZIF‐67	[176]
MoS_2_@Ni‐MOF	In situ hydrothermal method	1590.2 F g^−1^ (1 A g^−1^)	72.9 Wh kg^−1^	0.3 kW kg^−1^	89.0% (10 000 cycles)	Stabilization through ‐S bonds	Large internal resistance	[179]
Co‐MOF@WS_2_	Ex situ hydrothermal method	2901 C g^−1^ (2 A g^−1^)	80 Wh kg^−1^	1.0 kW kg^−1^	83.6% (12 000 cycles)	Hierarchical structure	Low cyclic stability	[180]
NiCo‐MOF@WS_2_@NiVS	Physical Mixing method	1235 C g^−1^ (1.1 A g^−1^)	133 Wh kg^−1^	2.5 kW kg^−1^	88.0% (5000 cycles)	Binder‐free heterostructure	Limited cycle life demonstration	[181]
**Hybrid‐ion capacitors**								
Co‐MOF‐74/FGO	In situ hydrothermal method	416 mAh g^−1^ (0.1 A g^−1^)	240 Wh kg^−^^1^	10 kW kg^−1^	91% (2000 cycles)	Honeycomb structure	Electrolyte decomposition	[184]
Co‐MOF@MXCNF	In situ hydrothermal method	980 F g^−1^ (0.5 A g^−1^)	41.5 mWh kg^−1^	0.8 W kg^−1^	87% (16 000 cycles)	Unique (O_4_‐CoN_2_) coordination geometry	Lower Coulombic efficiency	[185]

In the domain of energy storage, the primary challenge is maximizing volumetric and areal energy densities. While the literature often reports exceptional metrics from ultrathin electrodes, we contend that the true test of MOF‐2D hybrids lies in their ‘device‐level fidelity.’ Bridging the gap to commercial application necessitates demonstrating performance with high mass loadings (>10 mg cm^−^^2^) and under lean‐electrolyte conditions. Consequently, future interface engineering must focus on constructing robust 3D percolating networks that can withstand the mechanical stress of thick electrodes, shifting the paradigm from gravimetric excellence to practical, system‐level viability.

## 5 Conclusion and Outlook

In this review, we have highlighted the remarkable progress in designing hybrid architectures that integrate MOFs and 2D materials. Over the past decade, this field has progressed from early explorations of physical mixtures to a sophisticated discipline centered around dimensional interface engineering. This approach has proven crucial for effectively combining the ultra‐high porosity of MOFs with the robust charge‐transport networks of 2D materials. The resulting hybrids have demonstrated transformative potential across a range of applications, from electrocatalysis to advanced batteries. However, despite the exciting progress, significant challenges remain in moving from laboratory‐scale successes to scalable, real‐world applications. These challenges primarily arise from the need to reconcile atomic‐level precision with the scalability required for macroscopic devices, as well as the incomplete understanding of the dynamic interfacial processes involved.

First, mastering the fundamentals requires a shift from traditional empirical approaches to a more controlled and programmable strategy for interfacial assembly. A major challenge lies in moving beyond the current stochastic synthesis methods to achieve precise control over the MOF‐2D interface. The interface between the MOF and 2D material is central to the performance of these hybrids, influencing key properties such as electronic conductivity, stability, and catalytic activity. Current methods often rely on empirical trial‐and‐error, leading to inconsistencies in the interfacial structure. To overcome this, future research should focus on strategies such as epitaxial templating, which allows for precise alignment of 2D materials on MOF surfaces, ensuring a well‐ordered interface that enhances charge transport while maintaining the structural integrity of the MOF. Additionally, kinetic trapping should be utilized to stabilize high‐performance, metastable phases that are not accessible under thermodynamic equilibrium conditions. These strategies, along with techniques such as molecular layer deposition (MLD) for digital, angstrom‐level control of MOF growth, will be essential in developing materials with uniform, robust interfaces. Moreover, defect engineering will play a crucial role. Research should prioritize modulator‐assisted synthesis to create vacancy arrays or surface pockets on MOF facets, providing specific docking sites for 2D materials and reducing interfacial stress. Achieving single‐atom‐level interface control, where metal nodes on the MOF are exclusively coordinated to specific heteroatoms, will be vital for minimizing contact resistance and optimizing the material's performance.

Second, a rigorous mechanistic understanding is essential to fully realize the potential of MOF‐2D hybrids. The interface between the MOF and 2D material is dynamic and evolves under electrochemical bias, and it remains unclear whether enhanced performance arises from stable MOF‐2D coordination or from in situ generated species, such as amorphous metal (oxy)hydroxides, sulfides, or nanoclusters, which are formed during structural changes. To address this, future research must shift from focusing on pristine materials to investigating the dynamic SEI and evolving catalyst surfaces. Advanced in situ and operando techniques will be critical for real‐time monitoring of these changes. Techniques such as operando X‐ray absorption spectroscopy, which tracks oxidation states and coordination geometry, combined with surface‐enhanced Raman spectroscopy, which detects intermediate adsorbates and lattice vibrations, will provide valuable insights into the catalyst evolution. Differential electrochemical mass spectrometry (DEMS) will also be crucial for correlating structural changes with specific gas evolution pathways, distinguishing between catalytic turnover and lattice oxygen oxidation. On the theoretical side, models must evolve beyond standard gas‐phase DFT to include solvation effects, potential‐dependent grand canonical DFT, and microkinetic modeling, providing a more comprehensive understanding of the electrochemical interface. By linking experimental and theoretical efforts, we will be able to determine the role of the interface and distinguish between supports and active participants in catalysis.

Third, evaluating the performance of these materials need to go beyond traditional laboratory metrics and focus on real‐world conditions. While ultrathin electrodes have demonstrated impressive specific capacities in laboratory experiments, these metrics do not fully account for the practical limitations, such as sluggish ion transport, mechanical disintegration, and high tortuosity in thick electrodes. To address this, evaluations should prioritize areal (mAh cm^−2^) and volumetric (Wh L^−1^) capacities, which better reflect the true performance in practical applications. Engineering efforts should focus on developing 3D hierarchical networks that combine the conductive properties of 2D materials with the mechanical stability of MOFs. Such designs will be critical in mitigating the large volume changes observed in high‐capacity anodes or high‐sulfur cathodes. Additionally, the scalability of these materials must be addressed. Researchers need to optimize ink rheology, wettability, and mechanical flexibility to ensure compatibility with roll‐to‐roll processing. Furthermore, the materials must be validated in realistic form factors, such as pouch cells or membrane electrode assemblies, to better assess their commercial viability.

Finally, expanding the functional horizon will drive the next wave of innovation. The chemical space for both MOFs and 2D materials is far from exhausted. Exploring novel combinations, such as integrating covalent organic frameworks (COFs) or black phosphorus with multimetallic or stimuli‐responsive MOFs, promises to unlock unprecedented catalytic, sensing, or separation capabilities. This exploration should also be guided by a vision for broader societal and environmental impact. The development of sustainable synthesis routes using green solvents and earth‐abundant metals, coupled with early‐stage life‐cycle and techno‐economic analyses, will be crucial. By leveraging their tunable chemistry, MOF‐2D hybrids are well‐positioned to address global challenges not only in energy but also in water purification, environmental remediation, and biomedical sensing.

In conclusion, the next generation of MOF‐2D hybrid materials will be defined by their precision, adaptability, and practicality. By advancing interfacial engineering, gaining a deeper mechanistic understanding, and rigorously evaluating these materials under realistic conditions, this field is poised to make significant contributions to sustainable energy technologies. With the integration of advanced synthesis techniques, mechanistic insights, and sustainability considerations, MOF‐2D hybrids will transition from scientific curiosities to commercially viable solutions, playing a key role in shaping the future of energy storage, catalysis, and other critical applications.

## Funding

This work was supported by the ARC Laureate Fellowship (FL230100095), JST‐ERATO Yamauchi Materials Space‐Tectonics Project (JPMJER2003), National Science and Technology Council of Taiwan (111‐2124‐M‐002‐021, 111‐2628‐E‐002‐008, 112‐2221‐E‐002‐035‐MY3, 113‐2628‐E‐002‐002, and 113‐2926‐I‐006‐502‐G).

## Conflicts of Interest

The authors declare no conflict of interest.

## Data Availability

The authors have nothing to report.
